# Two-Dimensional Zeolite Materials: Structural and Acidity Properties

**DOI:** 10.3390/ma13081822

**Published:** 2020-04-12

**Authors:** Emily Schulman, Wei Wu, Dongxia Liu

**Affiliations:** Department of Chemical and Biomolecular Engineering, University of Maryland, College Park, MD 20742, USA; ebschul@umd.edu (E.S.); weiwuwuhan@gmail.com (W.W.)

**Keywords:** 2D zeolite, layered zeolite, zeolite structure, Brønsted acidity, Lewis acidity

## Abstract

Zeolites are generally defined as three-dimensional (3D) crystalline microporous aluminosilicates in which silicon (Si^4+^) and aluminum (Al^3+^) are coordinated tetrahedrally with oxygen to form large negative lattices and consequent Brønsted acidity. Two-dimensional (2D) zeolite nanosheets with single-unit-cell or near single-unit-cell thickness (~2–3 nm) represent an emerging type of zeolite material. The extremely thin slices of crystals in 2D zeolites produce high external surface areas (up to 50% of total surface area compared to ~2% in micron-sized 3D zeolite) and expose most of their active sites on external surfaces, enabling beneficial effects for the adsorption and reaction performance for processing bulky molecules. This review summarizes the structural properties of 2D layered precursors and 2D zeolite derivatives, as well as the acidity properties of 2D zeolite derivative structures, especially in connection to their 3D conventional zeolite analogues’ structural and compositional properties. The timeline of the synthesis and recognition of 2D zeolites, as well as the structure and composition properties of each 2D zeolite, are discussed initially. The qualitative and quantitative measurements on the acid site type, strength, and accessibility of 2D zeolites are then presented. Future research and development directions to advance understanding of 2D zeolite materials are also discussed.

## 1. Introduction

Zeolites are generally defined as three-dimensional (3D), crystalline, microporous aluminosilicates that have demonstrated enormous framework variety and pore connectivity [1]. The presence of aluminum (Al^3+^) in zeolites imposes net negative charges on the framework that are often counterbalanced by organic/inorganic cations or protons. Therefore, zeolites are endowed with ionic exchange capabilities as well as Brønsted acidity. Over time, these materials have been selected and manipulated to suit specific applications as both catalysts and molecular sieves [2,3]. In addition to the preparation and applications of 3D zeolite materials, the development of quantitative structure/activity relationships for chemistry in zeolites is being pursued. Various experimental methods have been explored to investigate zeolite acidity, including the quantification of acid site concentration, strength, and affinity. The structure/activity model built from acidity understanding was developed to describe the performances of zeolites in their corresponding applications. The reviews published by R. J. Gorte [4] and Y. Román-Leshkov [5] well-documented the results and progress on acidity investigation in 3D zeolite materials.

In comparison to 3D zeolites, two-dimensional (2D) zeolites are an emerging type of nanoporous materials [6,7,8,9,10,11,12]. They are often made from the 2D layered precursors that contain stacked sheets of one-to-two unit cell or smaller thicknesses that are linked by weak van der Waals forces or hydrogen bonds. The weak interlayer interactions in 2D layered precursors determine a variety of structural and chemical modifications that can be potentially applied within the gallery of adjacent layers while preserving the original layer integrity. Therefore, 2D layered zeolite precursors can be post-modified via intercalation, exfoliation, pillaring, etc. to make delaminated [13,14,15,16,17,18,19,20,21,22,23,24] and pillared [14,25,26,27,28,29,30,31,32,33,34,35,36] structures. These materials contain hierarchical micro- and mesoporosity within and between adjacent single-unit-cell or near single-unit-cell thick (~2–3 nm) nanosheet layers. The organic and/or inorganic pillars introduced during post-modifications enable the structural flexibility, compositional flexibility, and multi-functionality of 2D zeolites for various applications. 

In the area of catalysis, the defining characteristic of 3D zeolites is the presence of strong Brønsted acid sites, which are dispersed within regular pores of molecular dimensions. The confined space around the active sites and the restricted access to and from the internal surface give rise to the widespread use of zeolites as shape selective catalysts. In contrast, 2D zeolites contain extremely thin nanosheet slices of crystals that produce high external surface areas (up to 50% of total surface area compared to ~2% in micron-sized 3D zeolite) and expose a large amount of their active sites on external surfaces. Therefore, they enable beneficial effects on adsorption and reaction in processing bulky molecules. There are many reports about enhanced performance that can be achieved in catalysis when using 2D zeolite materials, especially for delaminated and pillared zeolite materials [36,37,38,39,40,41,42]. Overviews of 2D zeolites in catalysis have been published by J. Čejka [6,9,43] and J. Sun [10] in the past few years.

Along with the exploration of 2D zeolites in catalysis, the characterization of their structures and acidity is a necessity to understand their catalytic performances. Furthermore, structure and acidity comparisons between these novel zeolites and their 3D counterparts as facilitators for otherwise challenging reactions have proven to be effective in probing the intricacies of 2D zeolites, providing necessary quantifications for optimizing catalytic properties [10]. In a traditional 3D zeolite, catalytic active sites are present, predominantly in the micropores [44]. A more complex scenario exists in 2D materials, which is a result of the interface existing in the interlayer spacing and the nanometer-sized microporous layer [45,46]. First, the large amount of external surface area and the interface between meso- and micropores in 2D zeolites result in a high fraction of acid sites present in these locations relative to those in micropores. Secondly, the surroundings of acid sites change from the enclosed form to fully open or partially open conditions, which impacts their strength and affinity, as well as their consequent catalytic performances. In the literature, there have been sparse studies on the active site strength, quantity, and distribution in 2D zeolites, but no publication has systematically summarized and compared the acidity information for 2D zeolite materials. 

Here, we intend to deliver an overview of structure and acidity information on all available 2D zeolite materials. The review is presented in three sections. We first introduce the history of 2D zeolite precursor materials and their derivatives to acknowledge and understand the standard synthesis methods for the preparation of these materials. Next, we discuss the topological features of 2D zeolite precursors and their derivatives that influences the accessibility and location of Brønsted acidity in zeolites. Lastly, we focus on the overview of acidity characterization techniques and document results from sparse and isolated studies in individual research laboratories for comparison purposes. This forms a collection of acidity information to direct further research and development in this area. The challenges, strategies to overcome these challenges, and future research and development directions to advance 2D zeolite materials are also presented.

## 2. History of 2D Zeolite Precursors and Their Derivatives

**Figure 1:**
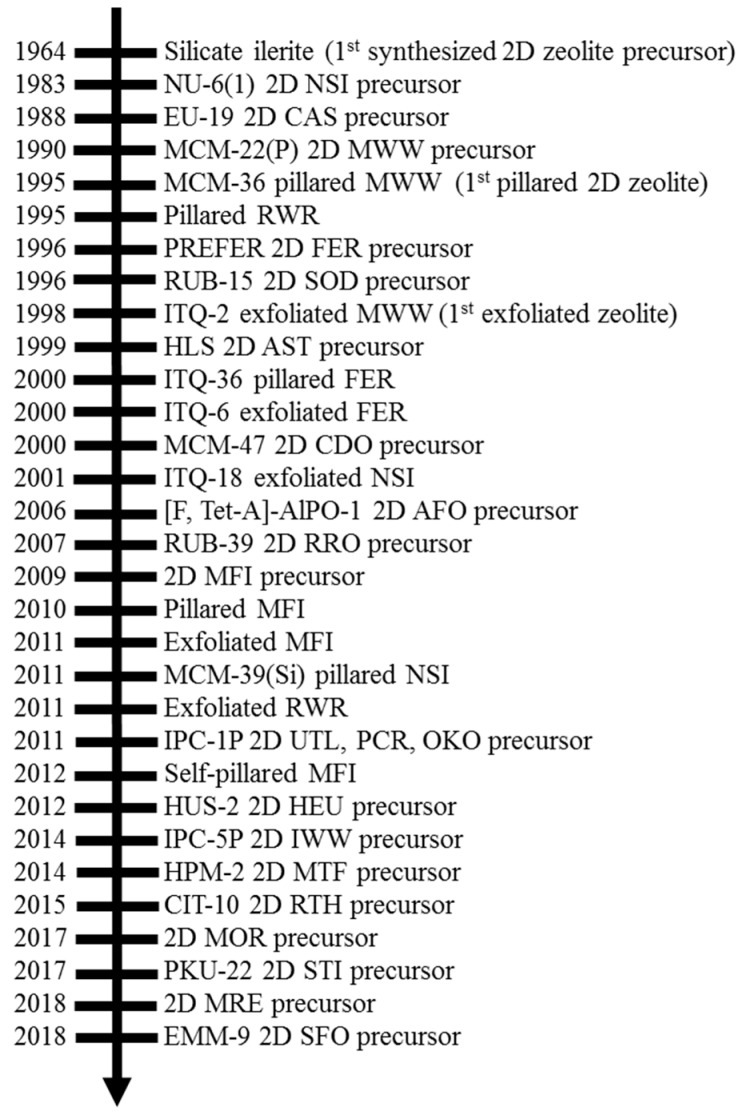
Timeline of the development of 2D layered zeolite precursors and their derivatives.

The history of the development of 2D zeolite materials consists of three stages: accidental occurrence during hydrothermal synthesis [47], the rational design of 2D zeolite nanosheets via templating [48], and the rational preparation of 2D zeolites via assembly–disassembly–organization–reassembly (ADOR) processes [49]. Figure 1 shows a brief review of the history of 2D zeolite development. The appearance of 2D zeolites first occurred by accidental discovery during the hydrothermal synthesis of a 3D zeolite in the 1960s, when researchers developed ilerite to produce an ordered silicate [47]. It was not until 40 years later that this material was discovered to be a 2D zeolite precursor (RUB (Ruhr University Bochum)-18) [50], and it was then used to hydrothermally synthesize RUB-24 [51], a 3D RWR (RUB-24 (twenty-four))-type zeolite. In a similar scenario, the 2D AST (aluminophosphate with sequence number sixteen) zeolite precursor, a β-helix-layered silicate (β-HLS), was first synthesized by Akiyama et al. in 1999 [52], and its structure was identified by Ikeda et al. in 2001 [53]. The successful topotactic conversion of β-HLS into the AST-type zeolite was reported by Asakura et al. in 2014 [54]. An excellent review on topotactic condensation of 2D layered silicate precursors into zeolite was published in 2012 [55]. 

Nu-6(1) (New (ICI, Imperial Chemical Industries) with sequence number six (one)), a precursor of zeolite type NSI (Nu-6(2) (six)), was the first officially reported 2D zeolite material, prepared in 1983 for synthesizing its 3D calcined counterpart, Nu-6(2) [56]. In the early stages of zeolite studies, a complete understanding of the crystallization mechanism was lacking, and, thus, proper control of the crystallization process and resultant zeolite morphology was infeasible. The emergence of 2D zeolite materials did not arouse intensive research interests until MCM (Mobil Composition of Matter)-22 (an MWW (MCM-22 (twenty-two)) topology) was reported in the 1990s [57]. Following this discovery, Edinburgh University (EU)-19 (NSI-CAS (cesium aluminosilicate) intergrowth) [58], RUB-15 [59], RUB-39 [60] and PREFER (precursor of ferrierite) [61] precursors for producing EU-20b, siliceous SOD (sodalite) zeolite, RUB-24, RUB-41, and ferrierite (FER), respectively, were discovered in similar synthesis processes. The implication of the layered MCM-22 zeolite material was to recognize that 2D zeolite precursors such as MCM-22(P) and others mentioned above could be modified into various hierarchical zeolite structures to include properties that differentiated them from conventional 3D zeolite materials with rigid structures. The first successful investigation into the design of a unique 2D layered zeolite used a basic reagent to produce swollen MCM-22(P), followed by the insertion of a silica precursor to stabilize the increased interlayer spacing via pillaring, which resulted in a new material, MCM-36 [25]. The inorganic pillaring process was modified and applied to additional zeolite structures such as ITQ (Instituto de Tecnología Química)-36 (FER) [14] and MCM-39(Si) (NSI) [32].

In the past decade, the construction of 2D zeolite materials from designed synthesis approaches has been an important research milestone in the 2D zeolite history. One remarkable breakthrough in recent years was the discovery of 2D MFI (ZSM (Zeolite Socony Mobil)-5 (five)) nanosheets, which was achieved by adopting gemini-type bifunctional surfactants as organic structure-directing agents (SDA) in hydrothermal synthesis [48]. These nanosheets have been treated with varying techniques to produce exfoliated 2D zeolites, which exist as stable single-sheet zeolite materials [13,15,18,62,63,64]. Another notable development in the application of 2D zeolites was the ADOR mechanism, which was reported in 2010 and used a 2D zeolite as an intermediate between the breakdown of one 3D parent zeolite structure and reconfiguration into new 3D or 2D daughter materials [31,65,66]. With improved synthetic techniques, 2D zeolite materials are not only increasing in number but also expanding in composition and structure. The one-step synthesis of unilamellar and self-pillared zeolite nanosheets with heteroatom compositions other than Al has been recently pursued. For example, MIT (Massachusetts Institute of Technology)-1 [67] and self-pillared pentasil (SPP) [40,68] have been prepared using the templating method in a one-step crystallization processes. Other aluminosilicate zeolites, such as MEL (ZSM-11 (eleven)) [69], FAU (faujasite) [70,71,72], TON (Theta-1) [73], MOR (mordenite) [74] and MRE (ZSM-48 (forty-eight)) [75], with 2D lamellar structures have also been explored, although the obtained layer structures have not been downsized into the unit-cell thickness level. 

According to the International Zeolite Association (IZA), about 5% of the current 200+ documented zeolite structures have been synthesized as 2D structures [9]. Several review articles have been excellently written on the topic of 2D zeolite materials, but their focus has remained on synthesis and structural characterization techniques [6,8,9,76,77]. We acknowledge the variety of 2D layered precursors and the derivative zeolite materials, as well as the scope of physical property alteration performed on these materials over the past few decades in the next section; then, we shift the focus of this article to a summary of acidity characterization on 2D zeolite derivative materials.

## 3. Structural Properties of 2D Zeolite Materials

Variations in properties across different zeolite structures contribute to the altered catalytic activity and selectivity for different chemical reactions. Differences in pore size, crystal structure, 2D layering technique, and chemical composition are some of the strongest contributors to zeolite catalytic performance through the alteration of acid site strength, accessibility, location, and concentration. In this section, we summarize the topological features of 2D layers with a zeolite topology and their derivatives. Zeolites are classified according to their pore openings, which consist of 6-, 8-, 10-, and 12-membered rings (MR) [78]. In most cases, the 2D zeolite layer is identified from its 3D zeolite framework. The corresponding three-letter code of that zeolite framework type is used as an acronym to designate the 2D layer topologies. If the particular type of 2D zeolite layer is already known as a layer-like building unit of a framework structure, we also use that name directly when discussing the corresponding structure and acidity properties.

**Figure 2:**
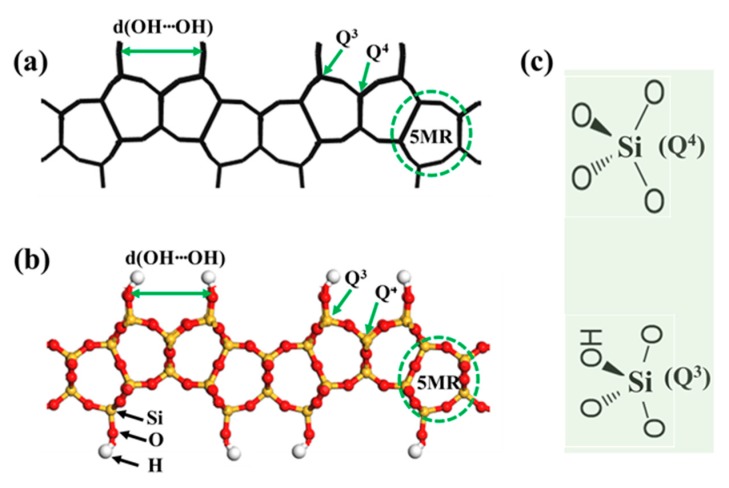
2D layered zeolite precursor (e.g., PREFER (precursor of ferrierite) as an example) with highlights of the -OH group, d(OH⋯OH), Q^3^ and Q^4^ coordination structures, and the definition of member ring (MR) in zeolites. Both line (**a**) and ball–stick (**b**) display styles are included. (**c**) shows the Q^3^ and Q^4^ structures in zeolites or their precursors.

In order to clarify the terminology used in description of layered zeolite structures, we use PREFER structure as an example (Figure 2) to illustrate the meanings of the hydroxy (-OH) group, the intra-layer distance between terminal hydroxyl groups [d(OH⋯OH)], the coordination structures of Q^3^ (i.e., three-connected [SiO_4_] tetrahedra) and Q^4^ (four-connected [SiO_4_] tetrahedra) groups, and the definition of a member ring (MR) (i.e., 5MR) in zeolites. Two atom display styles, line (Figure 2a) and ball–stick (Figure 2b), are shown to gain better understanding of the discussed structures.

### 3.1. 2D Layers with 6-MR Zeolite (AST and SOD) Topology

**Figure 3:**
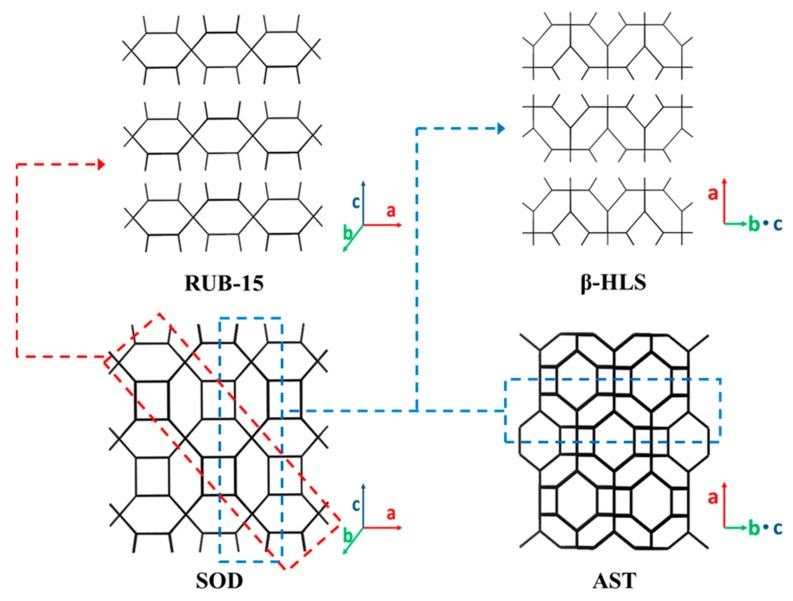
Schematic illustration and relationship between 2D layered silicates (β-HLS (β-helix-layered silicate) and RUB (Ruhr University Bochum)-15) and frameworks of 6-MR SOD (sodalite) and AST (aluminophosphate with sequence number sixteen) zeolites.

The 6-MR AST and SOD zeolites have 2D silicate precursor layers. It has been reported that β-HLS [79], HUS (Hiroshima University Silicate)-1 [80], HUS-5 [81], and RUB-55 [82] are the 2D layered precursors of 3D siliceous AST zeolite. RUB-15 [59], RUB-51 [83], and DLM (Delft Layered Material)-2 [84] have been reported to be 2D precursors of the siliceous SOD zeolite. Figure 3 depicts the layered silicate structures of β-HLS and RUB-15, as well as their structural relationship to the SOD and AST zeolite frameworks. Table 1 summarizes the structural properties of the 2D layered silicate precursors of AST and SOD zeolites in comparison to the related 3D counterparts. 

The framework of the β-HLS layer has a cup-shaped cage topology with 4- and 6-MR pores. Such a cage is comparable to a sodalite cage split into two fractions with trimethylammonium (TMA)^+^ cations incorporated into them as templates. Na^+^ ions and H_2_O molecules are located between two β-HLS layers, with two interlayer distances (4.0 Å and 4.6 Å) varying alternately. The layer thickness is ~7.2 Å, and the inter-layer distance of terminal silanol or siloxy groups (d(OH⋯OH)) is ~3.2 Å. HUS-1 and RUB-55 have similar structural properties to those of β-HLS, except that the inter-layer distance of HUS-1 (~1.5 Å–2.6 Å) is shorter and RUB-55 (~7.7 Å) is longer, respectively, than that of the 2D β-HLS precursor. In addition, the symmetry of these two types of 2D AST layers is slightly distorted compared to β-HLS silicate [82]. HUS-5 is the precursor of HUS-1 that undergoes fewer washings (one-to-three times) than HUS-1 (thorough washing) during preparation. The crystal structure of HUS-5 is the same as that of HUS-1, but the interlayer distance is ~4.0 Å, and TMA^+^, Na^+^, and hydrated H_2_O are present in the interlayer. 

For 2D layered silicate with an SOD topology, RUB-15 is formed by cutting out a section of the SOD framework perpendicular to the [011] direction (Figure 3). RUB-51 has the same 2D silicate layer as RUB-15 but with a different stacking sequence and template intercalation. The stacking sequence of the 2D layers in RUB-51 is AA rather than ABAB as in RUB-15 [83]. The obvious differences between the RUB-15 and DLM-2 layers lie in the space group and the resulting arrangement of the water molecules between the 2D SOD layers [84]. In comparison with those of AST layers, the SOD layers have a low Q^3^:Q^4^ (Q_3_: three-connected [SiO_4_] tetrahedral structure; Q_4_: four-connected [SiO_4_] tetrahedral structure) ratio and a larger inter-layer distance.

Both AST and SOD zeolites have very small micropore sizes (as listed in Table 1) and are not often considered for catalysis applications. In comparison to 3D AST and SOD structures, the 2D layered silicates with AST and SOD topologies have a sheet of cup-shaped voids made of 4- and 6-MR connections, which indicates possible site accessibility. All 2D AST and SOD layers are made of siliceous species. The HUS-1 layered silicate is silylated to form the dimethylsilane (DMS)-HUS material, which has 8-MR micropores in the interlayer space [85]. Except for this attempt, none of the other 2D layered precursors have been successfully delaminated or pillared to form 2D AST or SOD derivatives. One of the main challenges in developing 2D derivatives is the structural instability of these 2D precursor silicate layers. The removal of an organic template by acid washing or thermal treatment often leads to the formation of an amorphous structure. The other main challenge is the high Q^3^:Q^4^ ratio, which indicates significant H-bonding, which easily leads to self-condensation in post-treatment processes to form products with a reduced structural order. Given these considerations, 2D AST and SOD layered silicates have rarely been studied for the formation of any derivative structures, acidity properties, or catalysis applications.

### 3.2. 2D Layers with 8-MR Zeolite (CAS, CDO, MTF, NSI, RTH, RWR) Topology

In the category of 2D layers that have an 8-MR zeolite topology, six of them, CAS, NSI, CDO (CDS-1 (one)), MTF (MCM-35 (thirty-five)), RTH (RUB-13 (thirteen)) and RWR, have been reported. In particular, the CDO zeolite has a number of 2D layered precursors. Table 2 summarizes the structural properties of these 2D layered materials and their related 3D zeolite frameworks. EU-19 [58,88] and MCM-69(P) [89] are the 2D layered silicate precursors of CAS zeolite framework that contain 5- and 6-MR SiO_4_ tetrahedra. The interlayer space of EU-19 is occupied by piperazinium ions. The topotactic condensation of the silicate layers in EU-19 yielded EU-20b, which contains 88% CAS- and 12% NSI-type stacking zeolite [88]. The removal of piperazinium ions from EU-19 by methods other than calcination failed, and, thus, the delamination or pillaring of 2D EU-19 layers has not been reported. MCM-69(P) also contains piperazinium ions between two adjacent 2D zeolite layers but can be swollen, detemplated, and exfoliated in an aqueous solution [89] (third row in Table 3).

**Figure 4:**
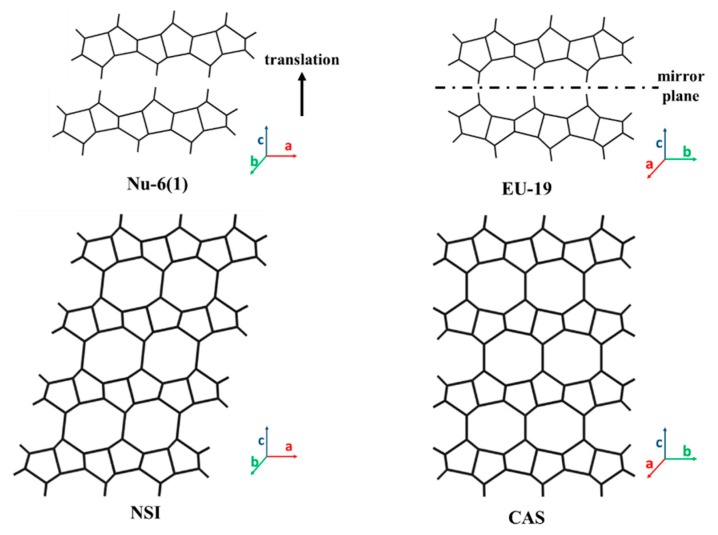
Schematic illustration of structures of 2D layered precursors and the corresponding NSI and CAS zeolites.

Nu-6(1) is another 2D layered zeolite precursor that has the same topology as that in EU-19, but the layers are skewed slightly from one another, as shown in Figure 4. This might be due to the replacement of piperazinium ions with 4,4′-bipyridine in the synthesis process [56,90]. The interlayer space in Nu-6(1) is larger than that of EU-19 because it hosts larger template molecules. The removal of template by the direct calcination of Nu-6(1) leads to the formation of Nu-6(2), a framework with an NSI-type structure. Unlike the siliceous 2D CAS zeolite precursors, the NSI 2D zeolite layers, Nu-6(1), can be synthesized with different Si/Al ratios, enabling the application potential as acid catalysts. With the askew layered structure, Nu-6(1) showed unusual performance during post-treatments to form derivative structures (see third row in Table 3). First of all, the intercalated template between 2D Nu-6(1) layers can be removed with an acid solution to produce organic-free MCM-39 lamellar product [32,104]. MCM-39 can be re-intercalated with various amines to form swollen NSI. The pillaring treatment of the swollen NSI enables the pillared derivative, MCM-39(Si), with permanently expanded interlayer separation and enhanced porosity [32]. Secondly, the 2D layers in Nu-6(1) can be exfoliated to form delaminated products such as ITQ-18 [15,105], Nu-6(2) [17], [V,Al]-ITQ-18 [106], and Del-Nu-6 [107]. Lastly, treatment by silylation forms IEZ (interlayer expanded zeolite)-Nu-6(1) in which adjacent layers are connected to form 10-MR micropores [108].

**Figure 5:**
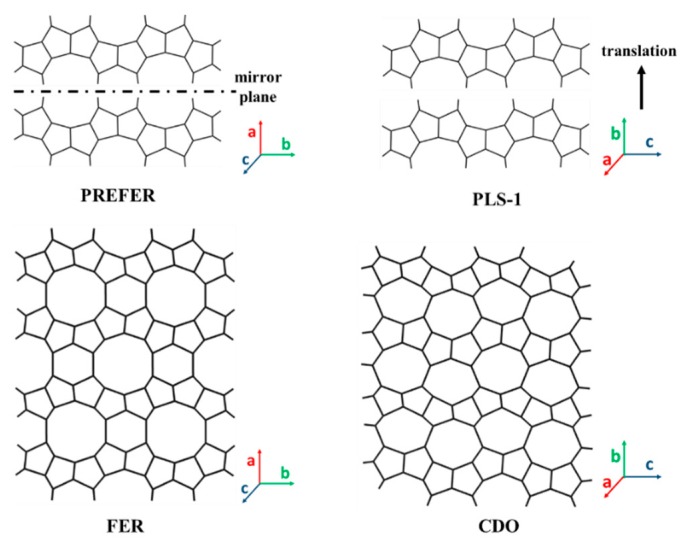
Schematic illustration of structures of 2D layered precursors and the corresponding FER and CDO zeolites.

As noted earlier, the CDO-type zeolite has many versions of 2D layered precursors (Table 2), the most common among which is the PLS (pentagonal-cylinder layered silicate)-1 layered silicate [92]. The PLS-1 framework contains high-density silicate sheets made up of 5-MR, and the pore-like interlayer space is occupied by TMAOH (tetramethylammonium hydroxide) molecules and K^+^ ions. The TMAOH template can be removed and recovered by heating PLS-1 above 673 K under vacuum and trapping the volatile components with liquid nitrogen. The sheets of PLS-1 condense and polymerize along the [100] direction by dehydration to form CDS (cylindrically double saw-edged)-1 zeolite with a CDO-type zeolite framework structure. The 2D layers with the same topology but different stacking sequences yield an FER zeolite (Figure 5). PLS-1 has many iso-structures, including PLS-4 [91], MCM-65 [122], MCM-47 [78], HUS-4 [96], ZSM-55 [97,98], ZSM-52 [99], RUB-series (e.g., RUB-20, 36, 38, 40, and 48) [93], and UZM (Universal Oil Products Zeolitic Material)-series (UZM-13, 17, and 19) [95]. The difference among these iso-structures is in the layer stacking, which results from the varied synthesis conditions, such as using different organic templates. For example, UZM-13, UZM-17, and UZM-19 were formed in the presence of diethyldimethylammonium (DEDMA), ethyltrimethylammonium (ETMA), and [Me_3_N(CH_2_)_4_NMe_3_]_2_^+^ (diquat-4, DQ-4) cations, respectively, in their syntheses [95]. Similar to most 2D layered materials that have siliceous compositions, PLS-1 and its 3D zeolite CDS-1 cannot behave as solid acid catalysts because the framework is constructed solely of tetrahedral SiO_4_ units. Recently, B- [123], Ge- [124], and Al- [95,116] containing PLS-1 and their layered iso-structures were prepared. CDS-1 zeolites with these heteroatoms were also made by direct calcination of these 2D layers. Derivatives of 2D layers with the CDO zeolite topology are currently being explored. As shown in Table 3, interlayer-expansion by the silyation [115,116,118,119,125] and pillaring [33] of PLS-1 has led to COD zeolites with expanded and pillared structures. 

**Figure 6:**
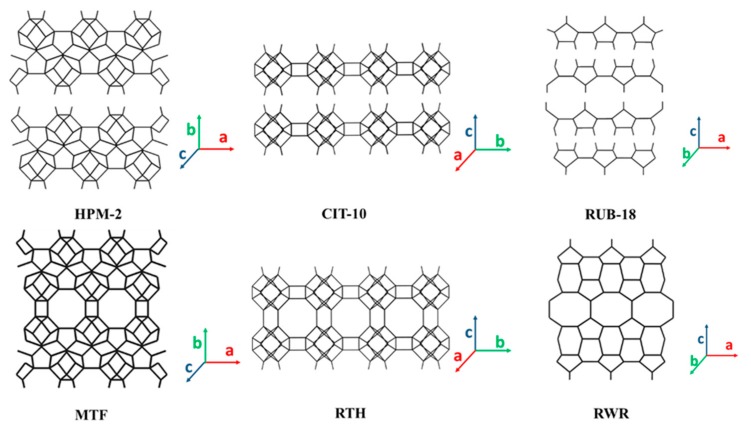
Schematic illustration of structures of 2D layered zeolite precursors, and their corresponding 3D MTF (MCM (Mobil Composition of Matter)-35 (thirty-five)), RTH and RWR zeolites.

HPM (nanostructured hybrid biohybrid and porous materials)-2 is a new layered organosilicate containing 2D layers with an MTF topology, yielding zeolite MTF by calcination [100]. The MTF structure contains 8-MR micropores along the c-axis direction, which separates the 2D layers normal to b-axis direction, as shown in Figure 6. Strong hydrogen bonds and close arrangement of silanols in adjacent layers exist in HPM-2. Therefore, attempts to delaminate or pillarize HPM-2 using previously reported recipes applied to other 2D layered materials failed. The interlayer expansion with dimethyldichlorosilane was successful, but the obtained material was non-microporous [100]. All 2D building layers discussed up until now are dense layers characterized by the fact that they do not contain 8-MR or larger pores perpendicular to the 2D layers. CIT (California Institute of Technology)-10, a layered precursor to the siliceous RTH zeolite framework, is an exception, as it contains 8-MR channels going through the layer (Table 2 and Figure 6). In addition, CIT-10 can be directly calcined to form pure-silica RTH (SSZ (Standard Oil Synthetic Zeolite)-50 [126]) or can be expanded by a silyating agent (e.g., dichlorodimethylsilane or diethoxydimethylsilane) to form CIT-11, which is stable following calcination (calcined material denoted as CIT-12) [101]. 

RUB-18, also known as octosilicate or ilerite, is the layered precursor of RWR zeolite [47,50,102,103]. The layered backbone is composed of four 5-MR pores as building units (Figure 6 and Table 2). Upon calcination, RUB-18 transforms into RUB-24, a zeolite with an RWR framework topology [51,127]. RUB-24 is a small-pore zeolite with a one-dimensional (1D) pore system consisting of straight and non-intersecting 8-MR channels. Though the structure analysis showed that the pores of the (idealized) silica framework are empty, nitrogen sorption experiments showed that there is no ‘‘free’’ access to the pore volume. However, compared to RWR zeolite, the 2D precursor, RUB-18, has many established capabilities, such as the interlamellar sorption of water and organic molecules, ion-exchange due to the interlayered hydrated counter-cations, and post-treatment to form multiple types of derivative materials (Table 3). For example, the original Na-form RUB-18 can be exchanged into the H^+^-form, which can behave as a proton conductor [103,109,128,129]. It can also be exfoliated into nanosheet layers [16,24], swollen to form organic-inorganic composites [30,130], and pillarized to form inorganic [110,111,112,131,132] or organic [113,114] pillared structures. Though RUB-18 does not contain heteroatoms, the pillarization treatment is able to introduce transition metals or organic functional groups that enable catalytic reactions over the derived 2D zeolite materials. 

### 3.3. 2D Layers with 10-MR (AFO, FER, HEU, MFI, MWW, RRO) Topology

In the category of 2D layered materials that have the 10-MR zeolite framework topology, six materials have been reported. Among them, one of them is aluminophosphate, while the rest of them possess the aluminosilicate composition. The presence of heteroatoms (e.g., Al) in the framework enables the materials to function as acid catalysts for catalysis applications, which is distinct from the 2D layers with 8-MR and 6-MR zeolite frameworks. In addition, the 10-MR micropores exist in some of these 2D layers (e.g., MFI and MWW), offering 2D layers that are directly capable of adsorption and catalytic applications. In addition, nearly all of them can be post-treated to form 2D derivative structures, as summarized in Table 4, Table 5, Table 6 and Table 7 below.

#### 3.3.1. 2D Layers with Aluminophosphate Framework

**Figure 7:**
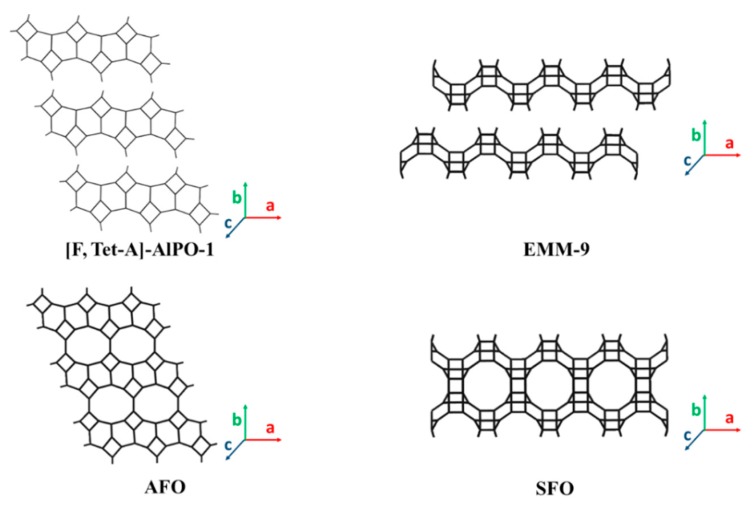
Schematic illustration of structures of 2D layered [F, Tet-A]-AlPO-1 and EMM (ExxonMobil Material)-9, as well as their corresponding 3D AFO ((AlPO_4_-41 (forty-one)) and SFO ((SSZ (Standard Oil Synthetic Zeolite)-51 (fifty-one)) zeolitic aluminophosphates.

Aluminophosphates (AlPO) are members of the zeolite framework materials. In comparison to ~20 2D zeolitic silicates and aluminosilicates, only a couple of 2D zeolitic aluminophosphates have been reported. One is the layered (fluoro)aluminophosphate, denoted as [F,Tet-A]-AlPO-1, which was the first reported 2D AlPO material [133]. The term “[F,Tet-A]” in [F,Tet-A]-AlPO-1 indicates the synthesis is done in a fluorine-medium (F) and uses the structure-directing agent azamacrocycle meso-5,7,7,12,14,14-hexamethyl-1,4,8,11-tetraazacyclotetradecane (Tet-A). The (100) layers in [F,Tet-A]-AlPO-1 resemble the AlPO-41 topology (framework-type code: AFO (AlPO_4_-41 (forty-one))) (Figure 7). The calcination treatment of [F,Tet-A]-AlPO-1 forms AlPO-41 with the AFO topology [134]. The translation of alternate (100) layers by 0.5a (~8.4 Å) and 0.5b (~4.8 Å) along with the a- and b-directions, respectively, followed by condensation in the c-direction, yields the AFO topology. 

The second layered (fluoro)aluminophosphate, denoted as EMM-9 (ExxonMobil Material #9), was reported recently by using a fluorine-medium and 4-(dimethylamino)pyridine (DMAP) as the organic structure-directing agent [135]. The 2D layers of EMM-9 are composed of STI (stilbite) composite building units, and DMAP cations are located between the layers. The layered EMM-9 structure is closely related to the 3D framework structure of EMM-8 and can be transformed to EMM-8 via calcination (Figure 7). EMM-8, which exhibits the SFO (SSZ-51 (fifty-one)) framework, contains 12- and 8-MR channels. Adjacent layers need to translate by 1/3a (4.8 Å) and 1/2b (6.8 Å) relative to one another before they are condensed along the c-axis. Though EMM-9 belongs to the category of 2D layers with a 12-MR zeolite topology to be discussed in Section 3.4 below, we include it here due to its compositional similarity to [F,Tet-A]-AlPO-1. The current studies on 2D layers with AFO and SFO AlPO zeolite topologies have been focused on synthesis and structure elucidation. 2D derivatives of these two 2D layered framework types have not been reported in literature.

#### 3.3.2. 2D Layers with FER Framework Topology

FER zeolite is a medium-pore aluminosilicate, including 2D intersecting channels with 8-MR channels (4.8 Å × 3.5 Å) along the [010] direction and 10-MR channels (5.4 Å × 4.2 Å) along the [001] direction. The 2D layers with the FER topology cut through the 10-MR channels, and, therefore, they only contain 8-MR pores (see Figure 5 in Section 3.2). Due to its close structural relationship with CDO zeolite, the FER framework topology has been identified in a number of 2D layered precursors (Table 4 and 2D layer precursors of CDO in Table 2). The differences between the crystal structure among these related 2D FER precursors result from the stacking sequence of FER layers, i.e., the displacement of layers parallel to the a–b plane and/or interlayer distance [136], which is the consequence of incorporating different templates in syntheses. Among these FER precursors, the most prominent ones are PREFER, PLS-3, ICP (Instituto de Catálisis y Petroleoquímica)-2 and ERS (EniRicerche molecular Sieve)-12 (Table 4). 

PREFER was synthesized in a fluoride-media in the presence of 4-amino-2,2,6,6-tetramethylpiperidine template [61,160]. The layer orientation is very straightforward: The stacking of FER layers occurs along the a-direction without translation in the b- or c-directions. Upon template elimination by calcination, the ordered 3D FER structure is formed through the condensation of the surface silanol groups. The layered PLS-3 silicate was prepared by a solid-state reaction using an H^+^-form of layered silicate (kanemite) as a silica source and tetramethylammonium as the SDA. The structure of PLS-3 is similar to that of the PREFER layer but with a smaller interlayer distance [91,161]. ICP-2 can be obtained in fluoride medium from aluminosilicate gels, using the chiral cation (1R,2S)-dimethylephedrinium (DMEP) as the SDA. It is a core-shell structure where the shell is composed of the organic cations arranged as supramolecular dimers surrounding the inorganic FER cores [137]. Similar to PREFER, ICP-2 can also be obtained in an Al-free form. The layered ERS-12 silicate is synthesized using the TEOS (tetraethyl orthosilicate) and TMAOH templates, which can also crystallize as germanosilicate, but not as alumino- and titanosilicate [138]. Calcined ERS-12 is, however, very different with respect to the calcined PREFER. This is caused by the fact that half of the silanol pairs on neighboring layers remain uncondensed during calcination, preventing the formation of a fully connected FER zeolite framework. In order to obtain the ordered FER framework, the layer must be shifted by 1/3c and 1/2b.

Similar to 2D layers with a CDO topology, layers with a FER topology have many 2D derivatives since delamination, pillarization, and silylation have been practiced in this type of 2D material. The delamination of PREFER was first done by Corma and co-workers by swelling the precursor in an aqueous CTAB (cetrimonium bromide)/TPAOH (tetra-n-propylammonium hydroxide) solution (pH 12.5) followed by ultra-sonication to exfoliate the 2D layers [14,63,64]. The as-produced FER monolayers are named ITQ-6, which has partial amorphization due to high pH condition. In 2011, Katz’s group [162] used a mild non-aqueous condition that contained a mixture of CTAB, tetrabutylammonium fluoride (TBAF), and tetrabutylammonium chloride (TBACl) in a dimethylformamide (DMF) solvent to swell PREFER. Afterwards, concentrated HCl was added to result in delamination of the swollen PREFER (denoted as UCB (University of California at Berkeley)-2). The characterization shows that the UCB-2 material does not have an amorphous structure and maintains the 2D layer structural integrity. The pillarization of swollen PREFER formed pillared FER, designated as ITQ-36 [34,120]. ZSM-55 has 2D FER layers, and it condenses to the CDO topology upon calcination. The pillaring of ZSM-55 was done recently, and it produced an ordered pillared FER at room temperature, as well as a structure with disorganization and partial layer degradation at high temperature (373 K) [33]. The interlayer expansion of FER layers by silylation formed new zeolite structures with larger 12-MR micropores [115,163] and 14 × 12 MR [164] materials, as noted by Wu et al. Due to the prominent 8-MR pore structure within 2D FER layers and the diversification of 2D derivatives and compositions, 2D FER zeolites have been proven to be efficient catalysts for different reactions. Heteroatoms such as Al, Ge, Ti, and B can be incorporated into the structures as well (Table 5). The pillaring process introduced additional elements such as Fe [34], Cr [34], and Sn [165], which further diversifies the acidity of FER-based 2D zeolite materials. 

#### 3.3.3. 2D Layers with HEU Framework Topology

**Figure 8:**
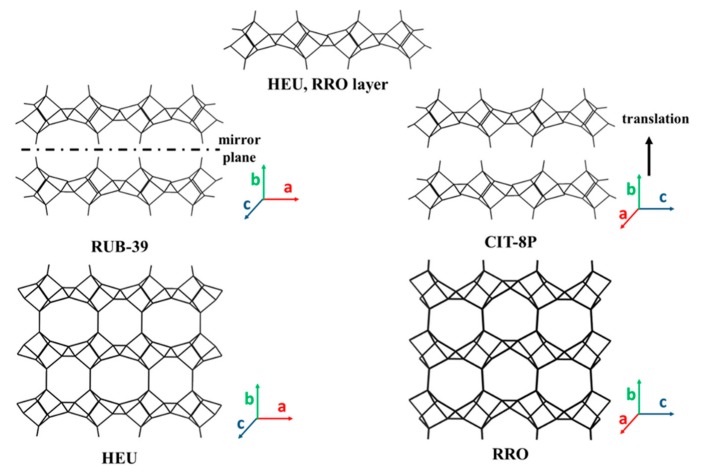
Schematic illustration of structures of 2D layered RUB-39 and CIT ((California Institute of Technology))-8P, and the corresponding 3D HEU and RRO zeolites.

As shown in Figure 8, the HEU (heulandite) zeolite framework contains a pore channel system with openings consisting of a 10-MR (3.1 Å × 7.5 Å) channel in the [001] direction as well as 8-MR micropores (3.6 Å × 4.6 Å) in the [001] direction (Table 4). Additionally, there is another set of 8-MR pores (2.8 Å × 4.7 Å) along the [100] direction. Materials with the HEU framework are divided into two distinct classes based on their Si/Al ratio. Those with an Si/Al ratio of less than four are known as heulandite, and those with an Si/Al ratio of greater than four are known as clinoptilolite or silica-rich heulandite. The 2D layers with an HEU topology are all high-silica layered aluminosilicate. CIT-8P is obtained from a low-water synthesis in fluoride media, with diquaternary amine as the SDA [139]. The condensation of CIT-8P by calcination produced CIT-8, which has the HEU topology. It should be noted that Ti and Al heteroatoms can be incorporated into the produced HEU zeolite. The layered silicates, HUS-2 [96] and HUS-7 [140], are comprised of 4-, 5-, and 6-MR with a framework topology similar to that of HEU-type zeolite. HUS-2 is synthesized using amorphous silica, sodium hydroxide, and choline hydroxide as the SDA. The change in SDA to biphenyl and benzyltrimethylammonium hydroxide led to the HUS-7 precursor. Silylation with the trichloromethylsilane of HUS-2 and subsequent calcination led to a microporous HUS-10 zeolite [166]. The Ti-species is intercalated into HUS-2, which leads to photooxidation applications [167], expanding on the siliceous derivative, which has only been considered for adsorption purposes. 

#### 3.3.4. 2D Layers with MFI Framework Topology

**Figure 9:**
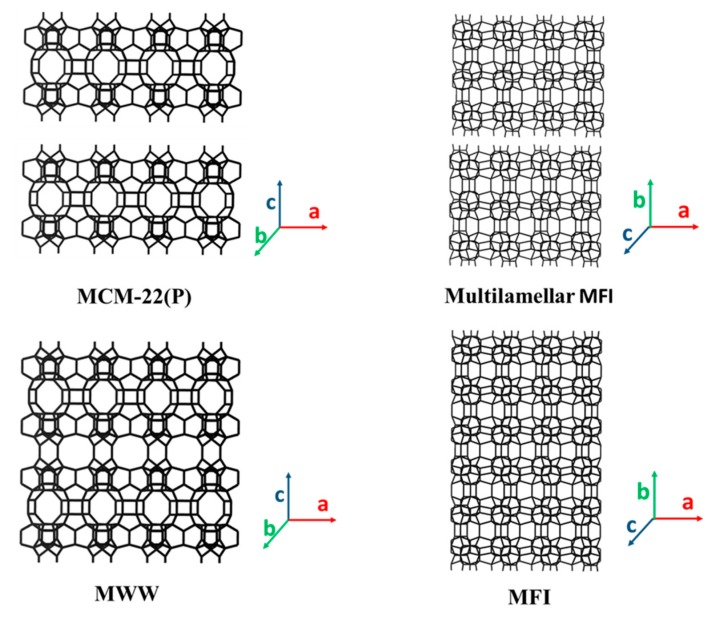
Schematic illustration of structures of 2D MWW (MCM-22 (twenty-two)) and MFI (ZSM (Zeolite Socony Mobil)-5 (five)) layered structures, and the corresponding 3D zeolites.

The MFI zeolite consists of two interconnected 10-MR channel systems: One is straight running along the b-axis direction (5.3 Å × 5.6 Å), and the other is zigzag running parallel to the a-axis direction (5.1 Å × 5.5 Å). The 2D MFI zeolite layers have the same zeolite micropore channels as those of the 3D, except the zigzag channel is lost due the layers being cut through this channel (Figure 9). The first synthesis of 2D layered MFI zeolite was achieved by Ryoo’s group in 2009, using the designed diquaternary ammonium surfactant, [C_22_H_45_–N^+^(CH_3_)_2_–C_6_H_12_–N^+^(CH_3_)_2_–C_6_H_13_][Br^−^]_2_ (denoted as C_22-6-6_), as the SDA [48]. Since then, 2D MFI zeolites with different nanolayer features (e.g., different layer thicknesses and interlayer distances) have been prepared using different SDAs (Table 4). For example, the C_22-6-6_ SDA led to an ordered multilamellar MFI structure in which each zeolite layer had a thickness of ~34 Å. The change of “–C_6_H_12_–” group in C_22-6-6_ into “–C_8_H_16_–” led to disordered zeolite nanosheets, and the thickness of the nanosheets was progressively increased according to the number of ammonium groups (N^+^(CH_3_)_2_) in SDAs [141]. In particular, the use of an SDA with the formula [C_18_H_37_–N^+^(CH_3_)_2_–C_6_H_12_–N^+^(CH_3_)_2_–C_6_H_12_–N^+^(CH_3_)_2_–C_18_H_37_][Br^−^]_3_ led to MFI nanosheets of 15 Å thickness, thinner than a single crystal unit-cell dimension (20 Å) [142]. The inclusion of biphenyl and naphthyl groups into the alkyl chain with a single quaternary ammonium head group in the SDA ([C_6_H_5_–C_6_H_4_–O–C_10_H_20_–N^+^(CH_3_)_2_–C_6_H_13_][Br]^−^) also forms ordered multilamellar MFI zeolite (named SCZN (single-crystalline mesostructured zeolite nanosheets)-1) [143]. Besides silicalite-1 and ZSM-5 compositions, 2D layered titanium silicalite-1 (TS-1) with ordered multilamellar structure was also prepared by using Ti-containing synthesis gel, C_22-6-6_ SDA and hexanediamine (C_6_DN) [144].

Inspired by the innovative syntheses of 2D MFI nanosheets using quaternary ammonium surfactant templates, a range of 2D MFI derivative structures have been prepared from direct hydrothermal crystallization. As summarized in Table 6, unilamellar [170,171,172], self-pillared [143,173], and nanosheet aggregates [174,175] with interconnected macro-/meso-/micropores have been created in the past decade. The unilamellar MFI nanosheets are synthesized when the SDA is in the hydroxide form (e.g., C_22_H_45_–N^+^(CH_3_)_2_–C_6_H_12_–N^+^(CH_3_)_2_–C_6_H_13_][OH^−^]_2_) [170,172]. The usage of nanocrystal-seeded growth triggered by a single rotational intergrowth in the presence of bis-1,5(tripropyl ammonium) pentamethylene diiodide (denoted as dC5) SDA also synthesized high-aspect-ratio MFI nanosheets with a thickness of 50 Å (2.5 unit cells) [171]. Self-pillared MFI (self-pillared pentasil, SPP) nanosheets were achieved by intergrowth with their 90° counterparts and with a small amount of MEL acting as a fourfold symmetric connector using the tetrabutylphosphonium cation as an SDA [173]. Following the usage of biphenyl and naphthyl groups in the SDA to synthesize SCZN-1, the usage of bolaform amphiphilic molecules with bi-quaternary ammonium head groups and biphenyl groups ([C_6_H_13_–N^+^(CH_3_)_2_–C_6_H_12_–N^+^(CH_3_)_2_–(CH_2_)_n_–O–C_6_H_4_–C_6_H_4_–O–(CH_2_)_n_–N^+^(CH_3_)_2_–C_6_H_12_–N^+^(CH_3_)_2_–C_6_H_13_][Br^−^]_4_) as the SDA synthesized MFI nanosheets joined with a 90° rotational boundary; this was named SCZN-2 [143]. All of these structures have an interconnected meso- and micro-porosity that facilitates mass transport for separation and catalysis applications.

Besides the direct synthesis method, traditionally practiced methods have also been used to produce 2D MFI nanosheet derivatives. The exfoliation of layered MFI nanosheets was first performed by Tsapatsis’s group using a polymer-melt-blending technique. Through the polystyrene melt blending, polymer removal, density gradient centrifugation, and redispersion steps, the as-obtained monolayer MFI nanosheets were further fabricated as molecular sieve membranes [18]. Following previous work, a more facile route was developed [19] in which the exfoliated multilamellar MFI zeolite from polystyrene-melt-blending was treated by a piranha solution to remove the organic residue. In 2017, Fan’s group reported the exfoliation of multilamellar MFI by suspending the layered zeolite precursors in telechelic liquid polybutadiene followed by brief shearing or sonication at room temperature [20]. The intercalation of TEOS into the multilamellar MFI zeolites followed by hydrolysis has been used to produce SiO_2_-pillared MFI [35,179]. Other metal oxide pillared cases have also been reported, such as titanosilicate [42], magnesium oxide, zinc oxide [180], and tin–silica [176]. An organic pillaring case was reported in Liu’s group, where acid extraction and UV light irradiation were sequentially employed to remove the SDA in multilamellar MFI zeolite, followed by intercalation of acrylic silsesquioxane (1,4-bis(triethoxysilyl)benzene, BTEB) molecules between multilamellar MFI layers [36]. It should be noted that the pillared MFI zeolites contain mesopores created by the inorganic pillar species sitting between MFI layers that is parallel to the zigzag channels and perpendicular to the straight channels within the layers. 

#### 3.3.5. 2D Layers with MWW Framework Topology

The MWW zeolite contains two independent pore systems. One system is defined by sinusoidal 10-MR channels with dimensions of 4.1 Å × 5.1 Å, and the other system consists of supercages delimited by 12-MR channels with dimensions of 7.1 Å × 7.1 Å × 18.1 Å. The consecutive supercages are connected through slightly distorted elliptical 10-MR windows (4.0 Å × 5.5 Å). One of the most prominent representative materials is the MCM-22 zeolite [57]. 2D layers with an MWW topology have many variations, which are differentiated by the layer ordering and interlayer repeat due to different synthesis conditions. Among these, the most prominent layers are MCM-22(P) [57,145], EMM-10P [146,147], ERB (EniRicerche-Boralite)-1 [148,149], MCM-56 [62,150,181], UZM-8 [152], SSZ-70 [153,154] and UJM-1P (Uniwersytet Jagiellonski Material #1) [157]. MCM-22(P) has layers stacked in vertical alignment with separation 2 Å longer (27 Å c-unit cell repeat) than in the complete 3-D framework (25 Å repeat) to which it converts upon calcination; hydrogen bonding between surface silanols was proposed as the interlayer connection maintaining the aligned in-register stacking. ERB-1 is a borosilicate zeolite material whose random stacking along the c-axis at well-defined distances is attributed to the piperidine molecules present in the interlayer region [148]. EMM-10P is closely related to MCM-22(P), but its layers are stacked without vertical alignment and are believed to be twisted off-register or otherwise disordered in-plane but still hydrogen bonded through silanols [147]. Upon calcination, EMM-10P partially converts to an ordered MWW structure, but, to a large extent, the stacking disorder persists when the layers fuse together. MCM-56 is a non-ordered material that can be regarded as a single layer collection, i.e., ‘partially delaminated,’ immature MCM-22 [182]. UZM-8 has a similar framework topology to that of MCM-56, but its inter-layer distance is larger than that of MCM-56 and smaller than that of MCM-22(P) [152]. UJM-1P is a new multi-layered and slightly expanded 2D MWW precursor, which was obtained by prolonging synthesis of the mono-layered MIT-1 material (see discussion in next paragraph). It is easier to swell with surfactants than MCM-22(P), which indicates a weak interlayer connection that may be due to the special SDA molecules lining the surface of its layers [157]. The as-synthesized SSZ-70(P) using imidazolium SDAs has a layered structure possessing some feature similarities to MCM-22(P), but the calcined form (e.g., SSZ-70) has different crystallographic features and catalytic performance to those of MCM-22 [153,154]. 

2D layers with an MWW zeolite topology have been explored extensively to produce numerous derivative structures. Delamination, pillarization, and interlayer expansion have all been investigated using these types of precursors (Table 7). First, MCM-22(P) has undergone numerous post-synthetic treatments that result in a plethora of layered and unilamellar zeolite structures [76]. A unilamellar MWW structure has also been developed by the delamination of the layered precursor and swollen material, resulting in MCM-56 [150] and ITQ-2 [13], respectively. More recently, the one-pot synthesis of MWW nanosheets was successfully implemented using an organic SDA containing both hydrophobic and hydrophilic portions to produce the desired 2D MWW structure that contains disordered single-layer MWW averaging 25 Å thickness and 150 nm length [67]. Another method of producing delaminated MWW nanosheets involves exfoliation in a mildly basic aqueous solution of pH 9, which results in the successful delamination of MCM-22(P) to form UCB-1 [22], and the Al-SSZ-70 zeolite precursor to form UCB-3 [183]. Pillared MWW (PMWW) was created by pillaring the MWW layers with SiO_2_, maintaining the 10-MR sinusoidal channels and hourglass shaped pores (half of the supercages in MWW) in the intact layers and mesopores between the layers. A silica source can be introduced as a post-synthetic technique to stabilize the gap between individual MWW layers and produce an ordered product (IEZ-MWW) from MCM-22(P) and disordered structure (EMM-10, EMM-12 [184]) from the disordered precursor, EMM-10P [147]. The slight interlayer spacing of MCM-22(P) can be manipulated further through swelling (MCM-22(P)-sw) with organic templates and pillaring for stability following calcination (MCM-36) [25]. SSZ-70 is available in silica, boron, and aluminum forms, all of which can be delaminated to form unilamellar structures [185,186]. 

#### 3.3.6. 2D Layers with STI and RRO Framework Topology

Zeolite RRO and STI also contain 10- and 8-MR channels, as shown in Table 4. The layered precursor (named PKU-22) with an STI topology was reported in 2017 [159], which is a silicogermanate and was hydrothermally synthesized under fluoride conditions using tetraethylammonium (TEA^+^) cations as the SDA. PKU-22 is constructed of STI layers stacked along the [100] direction, with TEA^+^ cations residing in the interlayer spaces and F^−^ anions existing within the layer and connected to Ge atoms, which also act as charge compensation species. Topotactic condensation was observed upon the heating of PKU-22, and the resulting product, PKU-22a, possessed an STI-type framework.

RRO zeolite has a two-dimensional channel system with intersecting 8- and 10-MR pores. The pore openings determined from structure analysis are 5.8 Å × 4.1 Å (8MR) and 5.9 Å × 4.1 Å (10MR). Similar to 2D layers with STI framework, the layered material with RRO framework only has one defined form (RUB-39) up until now. The synthesis of RUB-39 silicate was done by using dimethyldipropylammonium hydroxide as the SDA [60,196]. RUB-41, framework type code RRO, has been synthesized as a calcination product using the layered silicate, RUB-39, as precursor. The insertion of Al [169,197] and B [158] functional T-atoms into the layered precursor as well as its condensation to 3D framework silicate zeolites has been achieved. Silylation of the layered precursor RUB-39 with dichloro-dimethylsilane (DCDMS) providing layer interconnection the led to the formation of IEZ-RRO [168].

### 3.4. 2D Layers from 14- and/or 12-MR Zeolite (UTL, IWW, UOV, SAZ-1) Topology

**Figure 10:**
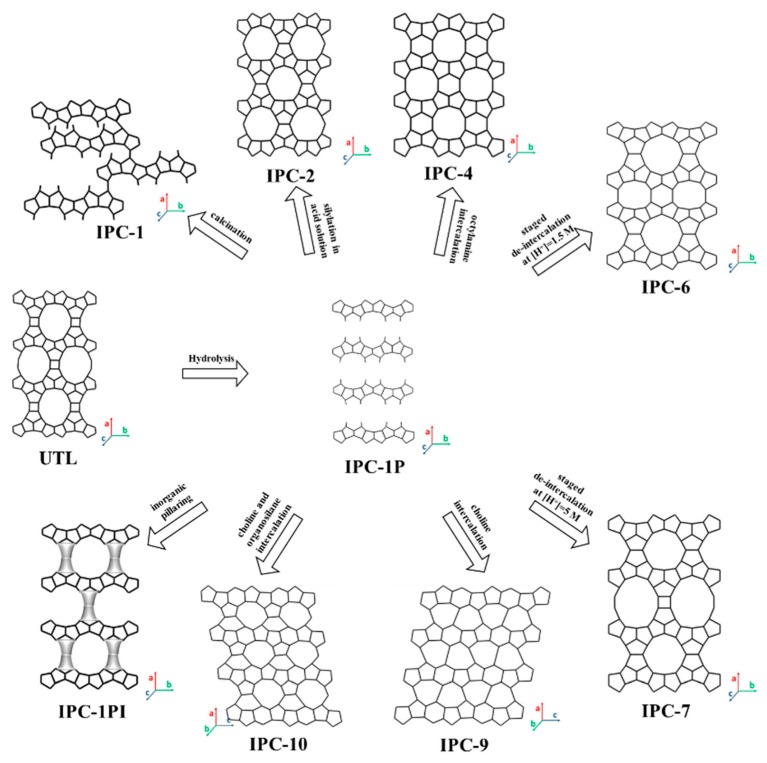
Schematic illustration of structures and processes for producing 2D layered intermediate and 3D or 2D zeolite derivatives by the ADOR method.

All of the 2D layered precursors discussed in the above sections are synthesized by hydrothermal crystallization of zeolite synthesis gels, i.e., the bottom-up synthesis. In this category of 2D precursor layers, the precursor is made from the pre-prepared parent zeolite in a synthesis process called the ADOR mechanism [49,198]. The key feature of the parent zeolite is the presence of a hydrolytically sensitive Ge dopant incorporated within the framework at a specific site (a double-four-ring (D4R) unit), which allows the chemically selective removal of the units containing the dopant. As a result, the germanium bonds such as Si–O–Ge or Ge–O–Ge (preferentially located within the D4R units) are selectively hydrolyzed, whereas the bonds within the layers, predominantly Si–O–Si bonds, are largely unaffected. This leads to the formation of 2D layered zeolite precursor materials, which can be treated with other post-methods to generate new zeolite structures and 2D zeolite derivatives (Figure 10).

The parent zeolites are large/medium pore materials, and the most extensively practiced zeolite materials include UTL, IWW (ITQ-22 (twenty-two)), UOV (Institut Français du Pétrole and University of Mulhouse—seventeen (one seven)), and SAZ-1 (University of St. Andrews zeolite—one) (Table 8). 

Germanosilicate UTL (IM (Institut Français du Pétrole and University of Mulhouse)-12, Mulhouse (twelve)) is an ideal ADOR starting point because of its chemical composition and stability of the layered units that are formed upon disassembly [199,200]. The resulting layered material was designated IPC-1P (Institute of Physical Chemistry-1 Precursor), which has a thickness of approximately 9 Å and possesses the same x–y projection as that of zeolite FER, although connectivity is more complicated in the z-direction, corresponding to a longer repeat unit, i.e., 12.5 Å vs. 7.5 Å. Similar to the FER precursor (PREFER), IPC-1P consists of rigid, compact layers that possess neither intra-layer zeolite-like channels nor well-defined inter-layer pores. IPC-1P has led to the formation of multiple types of zeolite materials, including IPC-1 (from direct calcination), IPC-4 (for layers connected simply through an oxygen atom), and IPC-2 (for layers connected through a single-four-ring unit (S4R)) [66]. The S4R connections and oxygen bridges produce two different inter-layer spacings, 11 Å and 9 Å, respectively. Furthermore, a medium/large pore zeolite, IPC-6 (12-10-MR and 10-8-MR pore systems), whose unit cell contains one of each of these different types of connections, has also been fabricated. A similar structure, IPC-7, has layer connections consisting of an equal quantity of S4Rs and D4Rs, possessing a unit cell consisting of one of each of these connections to produce a large-pore zeolite containing 14-12 and 12-10-MR pores [201]. IPC-1P can also be reconfigured using choline cations as the SDA to shift the layers with respect to each other to form IPC-9P. The layers can then be reassembled in two ways: by (1) calcination to form IPC-9 and by (2) calcination after intercalation of diethoxydimethylsilane to form IPC-10 [202].

Ge-rich IWW was treated with acidic solution at ambient temperature, leading to a layered material called IPC-5P with the inter-layer distance reduced by 1 Å to 3 Å depending on the applied conditions. IPC-5 is formed after calcination, confirming the successful application of the ADOR mechanism on an additional zeolitic structure—IWW [65]. In contrast to zeolite UTL, where two new zeolite structures (OKO (Oppervlakte en Katalyse One) and PCR (IPC-4 (four))) form from condensing the layered precursors via calcination, the layered structure, IPC-5P, tends to maintain the original IWW framework after calcination. Additionally, the ADOR transformation of a germanosilicate parent zeolite with a UOV topology produces a new zeolite named IPC-12 [207,208]. During this transformation, the pore system shifts from two dimensional, containing 12-MR and 8-MR channels along the [100] direction intersecting with a 10-MR channel, to a one dimensional pore system without the 10-MR intersecting channel. The ADOR method was also applied to a newly-discovered germanosilicate zeolite, SAZ-1, where an acid solution was used to remove the germanium-containing D4R units, producing a layered intermediate called SAZ-1P. SAZ-1P was further manipulated to produce IPC-15, whose layers were connected by O-linkages, and IPC-16, which contained S4R links between layers [209]. The same methodology has been applied to zeolites with ITH (Instituto de Tecnologia Quimica Valencia-thirteen), ITR (Instituto de Tecnologia Quimica Valencia-thirty-four), and IWR (Instituto de Tecnologia Quimica Valencia-twenty-four) topologies, but the resulting materials have not been explored with enough depth to fully elucidate the their structures [210]. 

In addition to the formation of microporous zeolite structures, the 2D layers formed form the ADOR process can be further treated to create swollen and pillared zeolites [31,203,204,205]. For example, swelling is performed using the cationic surfactant, hexadecyltrimethylammonium (CTMA), yielding IPC-1SW. The pillared derivative of this material was denoted as IPC-1PI. Isomorphically substituted B, Ti, Al and Fe have also been successfully incorporated in the layered precursor materials. It is important to note that these pillared materials do not possess microporosity because they consist of dense layers supported by amorphous silica pillars.

### 3.5. Other Types of 2D Zeolites (MEL, FAU, MOR, MRE, TON)

The successful synthesis of lamellar MFI zeolite inspired the exploration of other 2D zeolite frameworks containing heteroatoms and medium/large micropores. In the past decade, 2D lamellar zeolites with MEL [69], MOR [74], MRE [75], TON [73], and FAU [70,71] topologies have been prepared. It should be noted that these zeolites do not yet have unilamellar, multilamellar, or 2D derivative structures, unlike the 2D layered zeolites discussed in Section 3.1, Section 3.2, Section 3.3 and Section 3.4 above. Instead, they are nanosheet aggregates or nanosheet plates in which the nanosheets are tens of nanometers thick formed in a one-step hydrothermal crystallization process. Table 9 summarizes the structural and compositional properties of these 2D zeolites as well as the topological features of their 3D counterparts. 

The 2D MEL-type zeolite consists of titanosilicate (MTS (Multilayered Titanium Silicalite)-2) nanosheets synthesized using binary templates cetyltrimethylammonium tosylate (CTATos) and tetrabutylammonium hydroxide (TBAOH) [69]. The MTS-2 product exhibits a morphology of micro-sized nanosheet aggregates, in which each of the nanosheets exists in the range of 50–100 Å thickness, and it is inclined to orient along one direction. The 2D FAU-type zeolite X has a similar particle morphology to that of MTS-2, but the nanosheets (~70 Å thick) are organized in a house-of-cards-like assembly with wide macroporous interstices between the nanosheet stacks. The initial synthesis in 2D FAU zeolite used 3-(trimethoxysilyl)propyl hexadecyl dimethyl ammonium chloride (TPHAC) as the SDA [70]; later on, inorganic salts such as zinc nitrate and lithium carbonate were shown to produce similar particle morphologies [71]. 2D MOR, MRE and TON zeolites all have a plate-like morphology. The MOR layered zeolite was synthesized in the presence of the C_16_H_33_–N^+^(CH_3_)_2_–C_2_H_4_–N(CH_3_)_2_Br (C_16-2-0_) template [74], resulting in nanoplates with a wide dimension of about 3 µm on the a–b planes and 200–400 Å thickness along the c-axis direction. Further investigation into the synthesis parameters’ effects on developing layered MOR material determined that the structure and charge of the cationic gemini-type SDA played a critical role in determining morphology. As a result, hierarchical MOR displayed nanorod, nanobrick, and nanoplate morphologies under different synthesis conditions [211]. 3D MOR is characterized by a 12-MR main channel (6.5 Å × 7.0 Å) and a parallel 8-MR channel (2.6 Å × 5.7 Å) along the c axis, which are interconnected by 8-MR side-pockets (3.4 Å × 4.8 Å) along the b-axis. The as-produced 2D MOR nanoplate has a reduced thickness along the c-axis, which shortens the length of the 12- and 8-MR channels in the material. Though this material was referred to nanosheets in the original reports, the structural analysis down to the unit-cell level has not yet been assessed, and, therefore, we refer to the morphology as a nanoplate structure to provide this distinction. The 2D TON (i.e., ZSM-22) zeolite nanoplates, with a thickness of about 100 Å, were synthesized through hydrothermal crystallization using a 1-ethylpyridinium bromide template. The mechanism was assumed to be a multi-step crystallization process involving the aggregation and fusion of elementary nanorods, and inhomogeneous Al distribution was considered to be a key factor [73]. TON is known as a 1D channel system with 10-MR pore openings of 4.5 Å × 5.5 Å. The thickness of the nanosheets increased from 80 Å to 500 Å with increasing Al content in the starting mixture. Lastly, nanosheets possessing an MRE zeolite topology (LMZN) were synthesized using a benzophenanthrene template and a ((C_6_H_2_)_3_–(O–C_n_H_2n_–N^+^(CH_3_)_2_–C_6_H_12_–N(CH_3_)_2_(Br^−^))6 (denoted BPT_n−6−0_) surfactant. The nanosheets were composed of alternating ∼30 Å-thick zeolite layers and ∼40 Å -thick surfactant micelles [75].

## 4. Acidity Properties of 2D Zeolites

**Figure 11:**
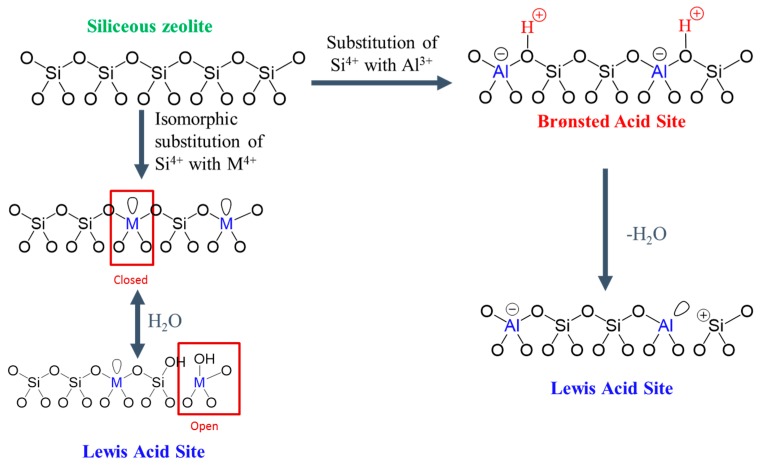
Schematic illustration for structures of Brønsted and Lewis acidity in zeolites.

The catalytic advantages of 2D zeolite materials, compared to their 3D analogues, are derived from the presence of active sites with appropriate acidity and improved accessibility. Acid sites are formed when heteroatoms are incorporated into zeolites via the isomorphous substitution of Si with other elements during direct hydrothermal synthesis or post-modification. The composition and coordination of heteroatoms determine the acidity type (i.e., Brønsted and Lewis) and strength, as shown in Figure 11. Enhanced acid site accessibility indicates the capability of 2D zeolites to process bulky molecules in catalysis. Relative to the multilamellar 2D zeolite precursors, increased acid site accessibility can be realized by the delamination and pillarization of 2D zeolite precursors; delamination increases external surface area and exposes the acid sites along the outward surface, and pillarization maintains and/or expands the gallery space between two adjacent zeolite layers, thus enhancing the accessibility of external acid sites. Though many studies have shown that higher conversions are achieved in catalytic reactions over 2D zeolites than their 3D microporous analogues, studies on effects of reduction in zeolite dimension on acidity properties have lagged behind. Therefore, very few reviews have been published on the acidity properties of 2D zeolites in past few decades [8,43]. In this section, we summarize the recent progress made on acidity characterizations of 2D zeolites, with focuses on the delaminated and pillared structures. It should be noted that many acidity measurement tools and protocols developed for 3D zeolites are equally applicable to 2D zeolite materials. Given the excellent review papers on these methods for 3D zeolites [4,212,213,214], we only focus on the introduction of techniques and acidity results that are obtained from the characterization of 2D zeolites. For visual significance, Figure 11 shows the representative structures of acidity in zeolite materials.

### 4.1. Techniques for Acidity Characterization of 2D Zeolite Materials

The acid site type and strength influence the activity and selectivity of 2D zeolite materials in catalysis, which are correlated to the coordination environment of heteroatoms. The most widely studied heteroatom in 2D zeolites is Al, therefore ^27^Al magic-angle spinning nuclear magnetic resonance (^27^Al-MAS-NMR) spectroscopy is commonly used to probe its coordination structures. In this method, the tetrahedrally coordinated framework Al atoms exhibit a signal in the range 51.5–65 ppm (depending on the type of zeolite and Si/Al ratio), while the octahedrally coordinated Al species (denoted as extra-framework Al) exhibit a chemical shift of 0 ppm [215,216]. In addition, a weak signal around 30 ppm is sometimes reported and ascribed to penta-coordinated Al species [43,217,218]. It is said that the shape and signal position of the ^27^Al-MAS-NMR spectra are independent of the crystal size, shape, and dimensionality of a particular zeolite [79,219]. The 2D zeolites, whether in delamination and pillarization form, however, often have higher fractions of extra-framework Al compared to their 3D analogues.

The FTIR spectroscopy of the hydroxyl (-OH) groups or adsorbate probe molecules is a well-established technique for evaluating the nature and strength of acid sites in zeolite materials. Depending on the zeolite framework and dimension, there are several types of -OH groups, including (i) lattice termination silanol (-SiOH) groups, (ii) –OH groups occurring at defect sites (hydroxyl nests), (iii) –OH groups attached to extra-framework heteroatoms, and (iv) bridging –OH groups (i.e., Brønsted (Si-OH-Al) acid sites). These –OH groups give rise to bands in the fundamental stretch region at ~3740, ~3720, ~3680, ~3600–3650 cm^−1^, in sequence [220,221,222,223,224,225]. The precise position of the –OH group in the Brønsted acid site varies with the zeolite’s framework structure and dimensionality [226,227]. 

The FTIR spectra of adsorbed probe molecules on acid sites in zeolites can provide information on the acid type and strength. These probe molecules are often organic bases and should be small enough to access all acid sites enclosed within the zeolite micropores. Pyridine, with a kinetic dimeter of 5.4 Å [45,228], is a commonly used organic probe molecule. The FTIR spectrum of adsorbed pyridine produces one band at around 1545 cm^−1^ due to the formation of pyridinium ions on Brønsted acid sites and another band at 1454 cm^−1^ due to pyridine ions on Lewis acid sites. The FTIR spectra of adsorbed pyridine can quantify the acid site concentration by combining the band intensity with the corresponding molar extinction coefficients by using the Lambert–Beer equation. The capability of retaining pyridine at different temperatures reflects the different strength of acid sites in zeolites. In general, the higher retaining temperature corresponds to stronger acid sites in the examined zeolites. Similarly, the acid site quantity and strength in zeolites can be probed by the temperature-programmed desorption (TPD) of amines such as ammonia (NH_3_). In the measurement, the catalyst is firstly saturated with NH_3_ at a lower temperature. A linear ramping of the temperature of the zeolite sample in a flowing inert gas stream is then applied, which results in a profile of TPD-NH_3_. The acid site concentration is determined from the amount of ammonia desorbing above some characteristic temperature in the profile, while the acidity strength is reflected by the peak desorption temperatures. The applications of the TPD-NH_3_ technique has been well described in previous review articles [4,213,229].

The quantitative analysis of the external acid sites in 2D zeolites is important to understand these materials’ catalytic performances in processing bulky reactant molecules. The concentration of total acid sites obtained from using a small organic base probe molecule, which can access all of the acid sites, combined with the concentration of acid sites determined using a bulky analogue that cannot enter the system of micropores, provides information about the distribution of acid sites in distinct locations of the 2D zeolites. A range of organic base molecules, as summarized in Table 10, have been paired together for this purpose. In the measurement, the uptake of small organic base molecules determines the density of total acid sites in the material, while the uptake of the large molecules measures the concentration of external acid sites. The ratio of adsorbed large base molecules to the small ones is the accessibility (or fraction) of the external acid sites in the analyzed 2D zeolites. This technique is often combined with FTIR spectroscopy, GC (gas chromatography) or MS measurement to quantify the adsorbed organic bases. In addition, the ^31^P MAS NMR spectroscopy of adsorbed phosphine oxides is a powerful tool for quantifying the external acid sites and acid site strength [67,79,230]. The method takes advantage of the strong adsorption of basic phosphine oxides (TMPO (tetramethylphosphonium) or TBPO (tributylphosphine oxide)) on Brønsted acid sites in zeolite framework, resulting in a one-to-one ratio. The interaction between a Brønsted (Si-OH-Al) acid site and an oxygen atom from phosphine oxide results in a ^31^P MAS NMR chemical shift that increases with the acid strength. Therefore, the chemical shifts of ^31^P MAS NMR signals indicate the acid strength, while the peak area suggests the quantity of the acid sites with the identified acid strength. TMPO and TBPO probe the total and external surface acid sites in zeolites, respectively, and the ratio of adsorbed TBPO to TMPO defines the accessibility of acid sites in the measured 2D zeolite.

### 4.2. 2D Zeolites Explored as Acid Catalysts

The structural and compositional properties of 2D zeolites and their derivative structures are summarized in Table 1, Table 2, Table 3, Table 4, Table 5, Table 6, Table 7, Table 8 and Table 9. The elements Al, Ti, Sn, Ge, and B are currently the dominant heteroatoms present in the available 2D zeolites, and they offer the Brønsted and/or Lewis acidity required for catalysis. The 6-MR 2D zeolites (e.g., AST and SOD) are only available in the siliceous form, and their pore sizes within the layers are too small to accommodate any hydrocarbon molecules for reaction. Therefore, no acidity studies have been conducted on this class of 2D zeolites. In the category of 8-MR 2D zeolites, NSI and RWR have isomorphously substituted heteroatoms and derivative (i.e., delamination and pillarization) structures, so acidity characterization has mainly focused on these two zeolite materials. For the 10-MR 2D zeolites, STI is a siliceous zeolite, while HEU and RRO do not yet have a delaminated or pillared structure. Hence, acidity characterization has been centered on the derivative structures of FER, MFI, and MWW zeolites. Due to the small pore sizes in the 2D layers of NSI, RWR, and FER, the acidity characterization has not been studied in great detail compared to 2D MFI and MWW zeolites. Lastly, the 2D layered IPC-1P, generated from the 14- and 12-MR parent zeolite, has B, Ge, and Al heteroatoms within its framework, as well as within that of its 2D derivative structures. The lack of microporosity in the 2D layers and structural stability limits the current research on the synthesis and structural elucidation of this class of materials. The acidity characterization and catalysis applications of these materials have not been explored extensively [240]. Given these considerations, the acidity characterizations for 2D zeolites have mainly focused on the derivatives of NSI, RWR, FER, MFI, and MWW zeolites, as sequentially introduced below. 

#### 4.2.1. Acidity Characterization for 2D NSI, RWR and FER Zeolites

Among the derivative structures of 2D NSI zeolites, the delaminated form (i.e., ITQ-18) has been explored for its acidity properties and compared with those of the 3D NSI structure (i.e., Nu-6(2)). The coordination structure of Al in ITQ-18 was characterized using ^27^Al MAS NMR spectroscopy, where the resulting spectrum shows a resonance at ~52 ppm for Al in tetrahedral coordination and an additional weak peak at ~0 ppm for extra-framework octahedral Al [15,41,106,178]. The presence of some extra-framework Al is attributed to the calcination step, which occurs immediately after the delamination treatment of its 2D layered precursor, Nu-6(1), since the latter does not show an obvious peak at ~0 ppm in its spectrum. Nu-6(2) has a comparable ^27^Al MAS NMR spectrum to that of ITQ-18, confirming the co-existence of framework and extra-framework Al after the direct calcination of Nu-6(1). FTIR spectra in the -OH region show that the Brønsted (Si-OH-Al) acid group has a band at 3610 cm^−1^ in ITQ-18, a similar position to that of Nu-6(2) [15]. In addition, FTIR spectra of adsorbed pyridine illustrate that the acid sites in ITQ-18 are accessible to pyridine, while Nu-6(2) has a negligible pyridine adsorption [15]. This suggests that the delaminated 2D NSI should have higher activity when carrying out acid catalyzed reactions than the 3D Nu-6(2) zeolite. 

For 2D zeolites with an RWR topology, only the pillared derivative structures have been studied for acidity properties because the heteroatoms, such as Al, were introduced during the post-treatment (i.e., pillarization) of the 2D layered materials. The ^27^Al MAS NMR spectra confirm the presence of tetrahedral framework Al in the pillared RWR zeolite [112,241]. When the Al content is high and the product was calcined, a small amount of Al exists as an octahedrally coordinated structure. The presence of Brønsted (Si-OH-Al) acid groups was characterized by the FTIR spectra of –OH groups in the pillared RUB-18 material, which showed a tail in the range of 3687–3413 cm^−1^ [241]. Compared to 2D zeolites with NSI and RWR frameworks, 2D zeolites with an FER topology have been studied more often in catalytic reactions [14,161,242,243,244]. The acidity properties of 2D FER zeolite in delamination and pillarization forms have been studied and compared to the 3D FER analogue. ITQ-6, the representative delaminated FER zeolite, was studied using both the ^27^Al MAS NMR and FTIR of –OH groups to determine the presence of tetrahedral framework Al, and the absolute acid site concentration was lower than that of 3D FER due to the dealumination during the exfoliation process [14]. The adsorption–desorption of pyridine at different temperatures traced by FTIR spectra has shown that ITQ-6 has a higher portion of Lewis acid sites and thus a lower portion of Brønsted aid sites than FER zeolite. Additionally, the fraction of strong acid sites is higher in the exfoliated form (i.e., ITQ-6) than that of the 3D FER zeolite [14]. The acid sites in both 2D ITQ-6 and 3D FER are accessible to pyridine, and the adsorption of di-tert-butyl peroxide (DTBP), which cannot access the acid sites within the 10-MR micropores of 3D FER, shows that the fraction of external acid sites is <5% in 3D FER, in contrast to ~90% in ITQ-6 zeolite [14]. The pillaring of 2D layered FER precursors has been extensively explored (Table 4 and Table 5). ITQ-36 is the earliest version of pillared FER zeolite [34], but the acidity characterization of this material is not available in open literature. The acidity characterization of another pillared 2D FER precursor (i.e., PLS-3) by both FTIR of –OH groups and ^27^Al MAS NMR spectra shows that the acidic Si-OH-Al groups are present in the FER framework, and the acid sites have higher accessibility to DTBP compared to the direct calcined PLS-3 zeolite [244]. 

#### 4.2.2. Acidity Characterization for 2D MFI Zeolites

Corresponding to the success of synthesizing 2D multilamellar MFI aluminosilicate and its derivative structures (Table 4 and Table 6), the acidity characterization for these 2D MFI zeolites has been intensively conducted. Acidity measurements have been done on the unilamellar, multilamellar, and pillared MFI structures and have been compared to the acidity properties of conventional 3D MFI zeolite (Table 11). As seen in the characterizations for other 2D zeolites discussed above, the ^27^Al MAS NMR technique has been used to examine the coordination environment of the Al element in 2D MFI zeolites [245,246,247,248,249]. The results have shown that, in randomly stacked (i.e., unilamellar) MFI nanosheets, the Al element is found not only in tetrahedral environments but also in octahedral and penta-coordinated/distorted four-coordinated positions [246]. Multilamellar MFI and its pillared form contain tetrahedral framework Al and a small amount of extra-framework Al species [48,245,248,249]. The quantitative analysis for fractions of extra-framework Al in these 2D MFI zeolites has been done by comparing the peak area of each species visible in the ^27^Al MAS NMR spectra. As shown in Table 11, multilamellar MFI contains more extra-framework Al than pillared MFI, while the 3D MFI has much less extra-framework Al compared to these two derivatives [245,249]. Overall, the fraction of extra-framework Al is indicative of the defective nature of 2D MFI zeolites, which is expected to follow the order of unilamellar MFI > multilamellar MFI > pillared MFI > 3D MFI. The FTIR spectra of –OH groups are used to recognize the Brønsted (Si-OH-Al) acid sites in these MFI zeolites. For all 2D and 3D MFI zeolites, the -OH vibration in Brønsted acid sites is located at ~3615 cm^−1^ [246,250]. The 2D MFI nanosheet, however, is dominated by –Si-OH groups on the external surfaces, which differs from the low peak intensity for this group in 3D MFI zeolite. 

The acid site type, quantity, and accessibility in 2D MFI zeolites have been measured by different techniques, including the FTIR of adsorbed small and bulky organic base molecules, organic base uptake measurement by GC/MS instruments, and ^31^P MAS NMR spectra (Table 11). For example, the FTIR spectra of adsorbed pyridine, collidine, and DTBP molecules have shown that the 2D unilamellar and multilamellar MFI zeolites have a higher fraction of Lewis acidity than that of 3D MFI, while the accessibility of acid sites is improved due to the open structure [247,248,249,250]. The acid site accessibility of pillared MFI and SPP zeolites has been measured by direct DTBP uptake measurements, which maintain the characteristics of the high acid site accessibility in 2D MFI zeolites [173,245,251,252]. In particular, the concentrations of external surface and pore mouth Brønsted acid sites in the pillared MFI zeolite was quantified by a combined dimethyl ether (DME) titration and methanol dehydration in the presence of DTBP or triphenylphosphine (TPP) titrants, respectively [232]. DME can access all Brønsted acid sites in pillared MFI zeolites and thus determine the total number of acid sites. DTBP cannot access acid sites in micropores, but it can access the external surface and at the pore mouth regions. In contrast, TPP can only access acid sites on the external surface. The degree of decrease in methanol dehydration rate in the presence of DTBP or TPP indicates the fraction of the sum of external surface and pore mouth acid sites and the sole fraction of external surface acid sites, respectively. It shows that pillared MFI contains ∼32% external surface and ∼6% pore mouth acid sites.

The acid site strength in 2D MFI has been measured by FTIR spectra of pyridine adsorption after desorption at different temperatures [247,249,253]. In comparison to 3D MFI zeolites, the unilamellar 2D MFI nanosheets have weaker acid strength. The TPD-NH_3_ profiles show that the MFI nanosheet exhibits a lower percentage of strong acid sites over total acid sites [254,255], consistent with the results from adsorption–desorption of pyridine measurement. In addition, the acid site location, strength, and accessibility in 2D MFI nanosheets were evaluated by Ryoo’s group using the ^31^P MAS NMR spectra of TMPO and TBPO probe molecules [79]. TMPO has a kinetic diameter of 5.5 Å [230], so it can access all external and internal acid sites. On the other hand, the TBPO molecule (∼8.2 Å) can be adsorbed exclusively on external surfaces for titration of external acid sites [44]. The spectra for the MFI zeolites were deconvoluted into multiple peaks with different chemical shifts, which were assigned to the chemisorption of probe molecules on Brønsted acid sites with four different strengths. Different from the acidity strength results obtained from FTIR spectra of adsorbed pyridine, the ^31^P MAS NMR spectra of TMPO show that 2D MFI nanosheets have high fractions of weak, medium, and strong acid sites, while the fraction of acid sites with medium-high strength is low compared to 3D commercial MFI zeolites. The external acid sites with three different (weak, medium, and high) strengths were determined by ^31^P MAS NMR spectra of TBPO in both 2D and 3D MFI zeolites. Similarly, 2D MFI nanosheets have high proportions of weak and strong acid sites, while the concentration of acid sites with medium strength is low. The external acid sites were calculated to be 32.0% of the total concentration of framework acid sites. The variation in fractions of external acid site accessibility in 2D MFI zeolites in Table 11 should be attributed to different measurement methods and variations in precise material structures produced from different research groups. Overall, the 2D MFI materials consistently show higher acid site accessibility than that of 3D MFI zeolite.

#### 4.2.3. Acidity Characterization for 2D MWW Zeolites 

Due to the aluminosilicate composition and medium micropore sizes, the acidity of 2D MWW zeolites has been extensively studied. Among many types of delaminated and pillared derivative structures, the ones generated from MCM-22(P) [57,145], MCM-56 [62,150,181], and SSZ-70 [153,154] have been the most widely studied. Over the past four decades, different protocols have been developed for the delamination of MCM-22(P) to form derivative structures. The earliest technique required a high-pH (in the range of 13.5–13.8) aqueous medium at an elevated temperature (e.g., 353 K) during MCM-22(P) swelling [104,256,257]. The swollen material was subsequently pillared to produce MCM-36 or exfoliated by ultrasonication to form ITQ-2 [13,258]. The ^27^Al MAS NMR spectra show a decrease in tetrahedral framework Al and a small increase in extra-framework hexa- or penta-coordinated Al when transiting from MCM-22 to ITQ-2 or MCM-36 [28,62,187,219,259], which confirms the dealumination and partial amorphization of the 2D zeolite layers during this approach. In 2010, Tspatasis and co-workers swelled MCM-22(P) at room temperature conditions [21,260], which preserved the layer and pore structure, as well as the zeolite composition, since both MCM-22 and MCM-36 showed similar peaks in the ^27^Al MAS NMR spectra. In 2011, Katz’s group exfoliated MCM-22(P) using a combination of tetrabutylammonium fluoride and chloride surfactants at pH 9 in an aqueous solution. The resultant product, UCB-1, showed the retention of tetrahedral framework Al at ~55 ppm and did not lead to the formation of extra-framework Al at ~0 ppm [22]. 

Similar to MCM-22(P), MCM-56 has been delaminated [182] and pillared [182,261] using the original protocol practiced for MCM-22(P). The ^27^Al MAS NMR spectra for the swollen and exfoliated samples contain signals for both framework and extra-framework Al. The intensity of the framework Al signal diminishes after swelling and sonication, in accordance with the decrease in the absolute concentration of framework Al and increase in extra-framework Al in this material. UCB-3 was synthesized by delamination of Al-SSZ-70 under non-aqueous conditions using fluoride anions [185]. The mild delamination conditions did not lead to leaching of framework Al to form extra-framework Al, which was consistent with UCB-1 synthesis.

Changes in acidity between the 3D and 2D MWW derivative structures have been further studied by monitoring the -OH vibration region in the FTIR spectra [28,62,258,262,263,264,265]. The results have shown that a larger number of -SiOH groups (bands at 3745 cm^−1^) are present on ITQ-2 and MCM-36 than those in 3D MWW, as is expected from the delamination process. Meanwhile, spectroscopic results in the Si-OH-Al region (~3615 cm^−1^) have shown a decrease after delamination, which has been caused by a certain degree of dealumination. Further analyses for the distinct positions of Si-OH-Al groups in the supercages (3621 cm^−1^) and in the sinusoidal channels (3608 cm^−1^) of MWW zeolites were also performed to provide a basis for comparison between 3D MWW (MCM-22) and 2D MWW (MCM-36) zeolites [226]. The signal belonging to Brønsted sites in the MWW supercages was about seven times weaker for 3D MWW than that of 2D MWW, indicating that the supercages are not re-formed when the zeolite does not condense into a 3D connected structure. 

To quantify the concentration and strength of acid sites in the MWW structures, the FTIR of adsorbed pyridine at different temperature conditions has commonly been enlisted (Table 12). The absolute quantity of both Brønsed and Lewis acid sites in MCM-22, ITQ-2, and MCM-36 has varied across different research groups [182]. Overall, the data have shown that the pillarization and exfoliation processes result in a decrease in the Brønsted acid sites, while the Lewis acid sites are either not affected or increased slightly [182,266]. Except for the very early work [187,258] that showed that pillared MCM-36 and exfoliated ITQ-2 had higher percentages of strong acid sites than that of 3D MCM-22 zeolite, most studies have drawn the opposite conclusion, in which the 2D MCM-36 or ITQ-2 has lower fractions of strong acid sites (Table 12). The synthesis of MCM-36 under mild conditions leads to a much higher concentration of Brønsted acid sites and a higher proportion of sites with medium-to-strong acidity. The MCM-36 prepared under high pH and high temperature conditions has a lower number of total Brønsted acid sites, in agreement with its lower Al content, but the fraction of stronger acid sites is high [260]. The acid site strength of 2D and 3D MWW zeolites is also characterized by profiles of TPD-NH_3_, and similar conclusions to those from FTIR of adsorbed pyridine have been drawn. For MCM-36 or ITQ-2 that are prepared under harsh (high pH and high temperature) conditions, three peaks are often observed in the profiles that consist of a maximum peak at ~608 K, a shoulder at ~498 K, and a characteristic shoulder in the range of 720–850 K. These peaks are sequentially assigned to the physiosorbed NH_3_ and NH_4_^+^ ions formed on strong Brønsted acid sites and NH_3_ adsorbed on the strong Lewis acid sites. In comparison to the MCM-22 parent material, MCM-36 and ITQ-2 have more strong Lewis acid sites, consistent with a partial dealumination during the swelling or pillaring processes [26,28,219,265]. Opposite to this trend, the MCM-36 or ITQ-2 prepared under mild conditions show lower acid site strength than MCM-22 [259,261,266].

The accessibility of acid sites to large molecules has been probed by FTIR of adsorbed bulky organic molecules such as DTBP [28,62,156,187,235,238,258,264,269], dimethylquinoline (DMQ) [226,262], 2,2,4-trimethylpentane [26], and pivalonitrile [182]. Along with the total number of acid sites that are determined from pyridine adsorption, the fraction of acid sites accessible to the bulky molecules have also been assessed, as shown in Table 13. It can be seen that there is a much larger concentration of external acid sites in ITQ-2 and MCM-36 than in MCM-22, a much larger concentration of external acid sites in pillared and delaminated MCM-56 than in directly calcined MCM-56, and a much larger concentration of external acid sites in IPC-3PI (pillared IPC-3) than IPC-3. This again supports the conclusion that the delamination and pillarization of 2D layered zeolites increase the number of Brønsted acid sites accessible to large molecules. Due to a higher degree of disorder along the c-axis in MCM-56 than in MCM-22(P) [62], MCM-56 has a higher fraction of external acid sites than that of MCM-22 (Table 13). 

DTBP titration, in combination with DME titration or the ethanol dehydration reaction, was used to evaluate the accessibility of acid sites in the MCM-36 prepared under mild conditions [173,245,251,252]. The ethanol dehydration rates as a function of cumulative DTBP or pyridine titrant addition on the MCM-22 and MCM-36 zeolites, respectively, were measured. Titration with DTBP over all zeolites initially resulted in a linear decrease in dehydration rates with the increasing addition of the titrant, consistent with the stoichiometric titration of the active sites along the catalyst bed. At saturation, DTBP titration maintained different residual rates over the zeolites with different pore structures. The loss in ethanol dehydration rates reflects the degree of accessibility of bulky DTBP molecules to Brønsted acid sites in zeolites with different pore structures and hence, the number of active sites accessible from the mesoporous environment. The calculation using results from this technique indicated that 8% and 67% Brønsted acid sites in MCM-22 and MCM-36, respectively, are accessible to the DTBP molecule. Compared to DTBP, the TPP molecule is sterically bulkier with a diameter at 9.4 Å [239], but it has a weaker base strength [270]. It can solely access the acid sites on the external surface [271]. The decrease in methanol dehydration rate in the presence of TPP titrant reflects the fractions of external surface and/or pore mouth acid sites in MCM-36 zeolite, where it consists of ∼33% external surface and ∼31% pore mouth acid sites [232]. 

^31^P MAS NMR spectroscopy was used to analyze acid site locations in the 2D MIT-1 structure [67]. The measurements were conducted for MCM-22 and MCM-56 zeolites for comparison. Similar to the results from the 2D MFI zeolite spectra, the ^31^P MAS NMR spectra of probe molecules (TMPO and TBPO) show peaks with different chemical shifts caused by the Brønsted acid sites with different acid strengths. In particular, MCM-22, MCM-56, and MIT-1 all have peak signals at 85, 72, 68, 63, and 53 ppm, of which the first four peaks are associated with strong Brønsted acid sites and the last one is due to TMPO adsorbed onto Lewis acidic extra-framework Al. Both MCM-56 and MIT-1 have much lower percentages of strong Brønsted acid sites than that of MCM-22. The total number of acid sites was quantified using spectra integration coupled with elemental analysis, which showed that MIT-1 has 64% external acid sites, higher than 41% external acid sites for MCM-56 and 13% for MCM-22. In ^31^P MAS NMR spectroscopy characterization for 2D MFI nanosheets [79] and MWW (MIT-1) nanosheets [67], it is important to note that both materials display one fewer type of Brønsted acid site on the external surface compared to inside the micropores. The Lewis acid site concentration in 2D MWW nanosheets seems to be high compared to that of 2D MFI nanosheets since the ^31^P MAS NMR spectra of TMPO adsorbed on MFI does not have the clear peak assigned to the Lewis acid sites; this is different from those of TMPO on MIT-1 nanosheets. This phenomenon is consistent with acid site titration results when using DME molecules reported by Liu et al. [245]. MCM-36 and MCM-22 both have a much lower concentration of Brønsted acid sites than Al concentrations, while MFI and PMFI (pillared MFI) zeolites have consistent concentrations of Brønsted acid sites and Al content.

#### 4.2.4. Acidity Characterization for 2D Materials Generated from UTL Zeolite

Presently, research has been focused on the synthesis of 2D zeolites and 3D zeolite structures derived from the 14- and/or 12-MR parent zeolites in the ADOR process. Thus far, acidity characterization has only been conducted on the 2D zeolites (e.g., IPC-1P and IPC-1PI) generated from UTL zeolites. IPC-1P contains individual ultrathin 2D layers with the UTL structure, while these layers of thickness ~9 Å possess neither intra-layer zeolite-like channels nor inter-layer pores. The 2D layers are separated by amorphous silica pillars in IPC-1PI. Therefore, the pillared IPC-1PI material does not exhibit any microporosity but possesses large mesopore voids created by the amorphous SiO_2_ pillars. The FTIR of –OH vibration regions and adsorbed CD_3_CN was employed to probe the type and concentration of acid sites in the IPC-1PI catalyst [272]. The most intense bands in the spectra, at around 3745 cm^−1^, were observed in the case of IPC-1PI, indicating a high concentration of silanol groups, which is characteristic of pillared materials with amorphous silica pillars. In contrast, the intensity of the band representing bridging -OH groups was very low compared to that of the UTL parent zeolite. The Brønsted acidity was greatly decreased, while Lewis acidity decreased slightly, according to the IR measurement of adsorbed CD_3_CN.

## 5. Summary and Outlook

The alteration of conventional 3D microporous zeolites into layered 2D zeolite structures has great potential to diversify zeolites and tailor their structures and acid site environments for catalysis applications. In 2D zeolites, the extremely thin nanosheet slices of crystals produce high external surface areas, up to 50% of total surface area, compared to ~2% in micron-sized 3D zeolite, and acid site accessibility is increased by exposing the majority of active sites on external surfaces. The implications of these unique properties of 2D zeolites—including the feasibility of converting bulky compounds that are incapable of entering the zeolite micropores; transport enhancement in handling bulky reactants, intermediates, and products; and less pronounced deactivation effects resulting from the formation of coke deposits—have been demonstrated in many catalysis studies. Overall, 2D zeolites show a significantly better catalytic performance than that of conventional 3D analogues. Despite the demonstrated success of 2D zeolites in advancing catalysis, there are still many challenges that can be addressed to improve and expand their future applications. This review summarized the types and structures, as well as the acidity characterization, of 2D zeolite materials reported in the literature. To better understand the acidity properties of 2D zeolites, the structural and compositional properties of 2D zeolite precursors, as well as their derivatives, were discussed in parallel. The research attempts in the area of 2D zeolites in the past few decades suggest certain future directions.

The most prominent topic in the development of 2D zeolites remains the synthesis of these 2D zeolite structures. Though a great number of zeolite structures have been synthesized in practice, a small fraction has been successfully made into 2D structures. Among the available 2D zeolite materials, the majority have been found by accidental synthesis. The templated synthesis of 2D MFI and ADOR methodology for the synthesis of IPC-1P inspire the feasibility of designed synthesis of 2D zeolite materials. To increase the number of 2D zeolite structures, innovative and creative synthesis methods are required. Accompanying the synthesis of 2D zeolite precursor materials, the post-modification of these precursors to produce diversified 2D derivative structures is also needed. Swelling, delamination, pillaring, and interlayer expansion are the four major methods practiced in formation of 2D zeolite derivatives. Among nearly twenty 2D zeolite precursors, six of them (FER, MWW, MFI, NSI, RWR, and IPC) have been explored in the formation of 2D zeolite derivatives, but only two of them (FER and MWW) have been extensively studied in terms of these four post-modification methods. The application of these post-modification methods to all the existing 2D zeolite precursors would increase the number of members of the 2D zeolite family. 

For the catalysis applications of 2D zeolite materials, the inclusion of heteroatoms into the zeolite precursor layers and derivative structures is vital to produce catalytic active sites. The chemical compositions of 2D zeolites, as shown by the statistics in Section 3, indicate that majority of current 2D zeolite precursors solely exist in the siliceous form. Only very few zeolites can be directly synthesized across the entire range of Si/Al ratios from 1 to infinity. The incorporation of heteroatoms such as Al, B, Ti, and Sn allows Brønsted and/or Lewis acidity to exist in 2D zeolite structures. Tactical synthesis by modifying the current recipes for producing 2D siliceous zeolites or by creating new procedures in the presence of heteroatoms in zeolite synthesis gels are needed in order to increase the compositional diversity in 2D zeolites. The post-modification of 2D zeolites can also provide a pathway to the inclusion of heteroatoms into their structures. The pillarization and inter-layer expansion methods have been used to bring heteroatoms such as Ti, B, and Sn into the zeolite pillars or frameworks, but this technique has been limited to only a few 2D zeolites. The diversification of heteroatom precursors and/or 2D zeolite precursors in post-modification processes will provide a great opportunity to create 2D zeolite derivatives with multiple types of acid sites and strengths. 

Thirdly, an understanding of the fundamental catalytic acidity properties of 2D zeolites is required. Since zeolite micropores present diameters very close to the size of many molecules, the shape selectivity and molecular sieving are unique properties of 3D microporous zeolites in catalysis. As the characteristic length of the micropore domain shrinks at the single- or near single-unit-cell level in 2D zeolites and the fraction of external zeolite surface area becomes comparable to that of micropore surface area as a consequence, the catalytic properties of the former (if not deactivated) become important or dominant contributors. Though the textural properties of layered 2D zeolite structures and their catalytic applications have been studied, the quantitative evaluation of acidity and acid site locations has not been extensively studied. The discrimination of acid site location and strength, especially in comparison to those of 3D zeolite analogues, will build a fundamental connection to the performance in product selectivity and activity in catalysis. More importantly, the understanding of location of active sites in zeolite frameworks—inside of the channels versus on the external surface—could assist in the rational design of 2D zeolites with specific acid locations, pushing capabilities beyond those of accidental discovery. 

Lastly, the advantages sustained by these 2D zeolites have been clearly demonstrated when compared to the activity seen in their 3D counterparts for certain catalytic reactions. However, there is still a lack of understanding regarding long-term, thermal, and hydrothermal stability of the more delicate layered or delaminated 2D zeolite structures. Hydrothermal stability has been investigated for micro-/mesoporous zeolites designed via organic structure-directing agents, resulting in the discovery of significantly lowered stability in these materials relative to their 3D equivalents [273]. This has been attributed to the amorphous structure developing around pore walls due to the SDA [274], but this phenomenon cannot be applied to the crystalline layered and single-sheet zeolite materials. The investigation into the hydrothermal stability of 2D zeolites under reaction conditions could determine their best-suited applications in catalysis and beyond to avoid structural breakdown. With extensive studies and achievements in the areas noted above, it is expected that 2D zeolite could substantially enhance and expand the possibilities of catalysis applications and further increase the widespread use of zeolites as catalysts in both laboratory studies and industry practice.

## Figures and Tables

**Table 1:** Structural properties of 2D layered precursors and their related 3D (6-MR) zeolites.

3D Zeolite Framework *^a^* (Pore Structure) (Å)	2D Layer Precursor (Layer Stacking direction) *^b^*	SDA in 2D Precursor Synthesis	2D Layer Pore Structure *^a^* (Å)	2D Layer Property			
				Q^3^:Q^4^ Ratio *^c^*	d(OH⋯OH) *^d^* (Å)	Layer Thickness (Å)	Inter-Layer Distance (Å)
AST (6MR: 1.7 × 2.9)	β-HLS [52,53,54] (a-axis)	TMAOH	6MR 1.7 × 2.9	4.1:1	3.2	7.2	4.0 and 4.6 *^e^*
	HUS-1 [80] (a-axis)	TMAOH BTMAOH	6MR 1.7 × 2.9	4.3:1	2.6	7.4	1.5–2.6
	HUS-5 [81] (a-axis)	TMAOH	6MR 1.7 × 2.9	4.9:1	-	7.4	4.0
	RUB-55 [82] (a-axis)	TMAOH	6MR 1.7 × 2.9	3.7:1	2.3	6.9	7.7 or 2.9 *^f^*
SOD (6MR: 2.5 × 1.8)	RUB-15 [59,86] (c-axis)	TMAOH	6MR 2.5 × 1.8	2.0:1	2.5	6.3	7.7
	DLM-2 [84] (c-axis)	TMAOH	6MR 2.5 × 1.8	-	-	-	-
	RUB-51 [83] (c-axis)	BTMAOH	6MR 2.5 × 1.8	2.0:1	-	-	8.8
	ULS-1 [87] (c-axis)	ETMAOH	6MR 2.5 × 1.8	2.0:1	-	-	8.3

*^a^* reported for the biggest micropore opening (i.e., 6-MR) in each zeolite; *^b^* layer stacking direction for 2D zeolite precursor; *^c^* Q^3^: three-connected [SiO_4_] tetrahedra structure; Q^4^: four-connected [SiO_4_] tetrahedra structure; *^d^* minimum intra-layer distance between terminal silanol (Si-OH) or siloxy (Si-O) groups; *^e^* inter-layer distance alternated between 4.0 and 4.6 Å for adjacent layers; and *^f^* 7.7 Å in hydrated precursor and 2.9 Å after dehydration.

**Table 2:** Structural properties of 2D layered precursors and their related 3D (8-MR) zeolites.

3D Zeolite Framework (Pore Structure) ^*a*^ (Å)	2D Layered Precursor (Layer Stacking Direction) *^b^*	SDA in 2D Precursor Synthesis	2D Layer Pore Structure *^a^* (Å)	2D Layer Property			
				Q^3^:Q^4^ Ratio *^c^*	d(OH⋯OH) *^d^* (Å)	Layer Thickness (Å)	Inter-Layer Distance (Å)
CAS (8MR: 2.4 × 4.7)	EU-19 [58,88] (c-axis)	Piperazine	6MR 1.9 × 2.6	0.5:1	6.0	8.3	3.2
	MCM-69(P) [89] (c-axis)	Piperazine	6MR 1.9 × 2.6	0.5:1 *^e^*	4.9 *^f^*	-	-
NSI (8MR: 2.6 × 4.5 8MR: 2.4 × 4.8)	Nu-6(1) [56,90] (c-axis)	4,4′-bipyridine	6MR 1.8 × 2.5	-	-	8.0	5.4
CDO (8MR: 3.1 × 4.7 8MR: 2.5 × 4.2)	PLS-4 [91] (b-axis)	DEDMAOH	5MR 1.1 × 1.5	-	2.2	-	11.1
	PLS-1 [92] (b-axis)	TMAOH and K^+^	5MR 1.1 × 1.5	-	-	-	10.5
	RUB-20 [93] (b-axis)	TMAOH	5MR 1.1 × 1.5	0.5:1	2.4	-	10.4
	RUB-40 [93] (b-axis)	TMPOH	5MR 1.1 × 1.5	0.4:1	2.6	-	10.6
	RUB-36 [93] (b-axis)	DEDMAOH	5MR 1.1 × 1.5	0.3:1	2.4	-	11.1
	RUB-38 [93] (b-axis)	MTEAOH	5MR 1.1 × 1.5	0.3:1	2.4	-	11.3
	RUB-48 [93] (b-axis)	TMPAOH	5MR 1.1 × 1.5	0.3:1	2.4	-	11.1
	MCM-47 [78] (b-axis)	TMMPBr	5MR 1.1 × 1.5	0.3:1	2.2	-	11.2
	MCM-65 [94] (b-axis)	Quinuclidine and TMAOH	5MR 1.1 × 1.5	1:1	2.7	-	11.3
	UZM-13 [95] (b-axis)	DEDMAOH	5MR 1.1 × 1.5	0.3:1	2.5	-	11.1
	HUS-4 [96] (b-axis)	Choline hydroxide and Na^+^/K^+^/Rb^+^/Cs^+^	5MR 1.1 × 1.5	-	-	-	-
	ZSM-55 [33,94,97,98] (b-axis)	choline chloride	5MR 1.1 × 1.5	0.3:1	-	-	11.2
	ZSM-52 [94,99] (b-axis)	choline chloride	5MR 1.1 × 1.5	-	-	-	-
MTF (8MR: 3.6 × 3.9)	HPM-2 [100] (b-axis)	2E134TMI	6MR 1.5 × 2.9	0.3:1	2.5	-	17.5 *^g^*
RTH (8MR: 3.8 × 4.1 8MR: 2.5 × 5.6)	CIT-10 [101] (c-axis)	diquaternary imidazoles	8MR 2.5 × 5.6	0.3:1	-	-	11.8 *^g^*
RWR (8MR: 2.8 × 5.0)	RUB-18/ilerite [47,50,102,103] (c-axis)	sodium	5MR 1.1 × 1.7	2:1 or 1:1 *^h^*	2.3	7.1	2.0

*^a^* reported for largest micropore opening and micropores with greater than 6MR in each framework; *^b^* layer stacking direction for 2D zeolite precursor; *^c^* Q^3^: three-connected [SiO_4_] tetrahedra structure; Q^4^: four-connected [SiO_4_] tetrahedra structure; *^d^* minimum intra-layer distance between terminal silanol (Si-OH) or siloxy (Si-O) groups; *^e^* Q^3^:Q^4^ for ratio for calcined MCM-69(P), MCM-69; *^f^* minimum distance in MCM-69; and *^g^* d-spacing distance in 2D zeolite precursor; *^h^* B-ilerite: Q^3^:Q^4^ ratio = 1:0.5; H-ilerite: Q^3^:Q^4^ ratio = 1:1.

**Table 3:** Structural and compositional properties of 2D layered derivatives of AST (aluminophosphate with sequence number sixteen), CAS (cesium aluminosilicate), NSI ((Nu-6(2) (six)), CDO (CDS-1 (one)), RTH (RUB-13 (thirteen)) and RWR ((RUB-24 (twenty-four)) zeolites.

3D Zeolite Framework	2D Layered Precursor	Re-Organizing Method	Derivative Structure Property				
			2D Zeolite Derivative	Inter-Layer Pore Formed *^a^*	Layer Heteroatom Composition	Pillar Heteroatom Composition	Inter-Layer Distance (Å)
AST	HUS-1	silylation	DMS-HUS [85]	8MR	-	-	1.8
CAS	MCM-69(P)	detemplated	MCM-69 [89]	-	Al	-	-
		delaminated	[89]	-	Al	-	-
NSI	Nu-6(1)	detemplated	MCM-39 [32,104]	-	Al	-	1.7
		delaminated	ITQ-18 [15,105]	-	Al	-	-
			Direct exfoliated Nu-6(2) [17]	-	Al	-	-
			[V,Al]-ITQ-18 [106]	-	V, Al	-	-
			Del-Nu-6 [107]	-	Al	-	-
		inorganic pillared	MCM-39(Si) [32]	30 Å	Al	-	28.8
		silylation	IEZ-Nu-6(1) [108]	10MR 4.8 Å × 5.8 Å	Al	-	-
RWR	RUB-18/ilerite	detemplated	octosilicate [109]	-	-	-	-
		delaminated	Ex-bim-Oct [24]	-	-	-	-
			(C_10_)_2_DMA-Oct [16]	-	-	-	-
		inorganic pillared	Silica- pillared [110]	10 Å	-	-	25.9
			Ta-, Nb-, Si- pillared [111]	mesopore	-	Ta, Nb	12.9–18.0
			Ti-, Al- Zr- SiO_2_-pillared [112]	20 Å	-	Ti, Al, Zr	20.3–30.3
		organic pillared	B-ilerite [113]	-	-	-	12.2
			RUB-N, RUB-2N, RUB-3N [30]	-	-	-	11.5, 23.1, 30.9
		silylation	APhS-ilerite-2 [114]	-	-	-	-
RTH	CIT-10	silylation	CIT-12 [101]	10MR	-	-	-
CDO	MCM-47	silylation	IEZ-CDO [115]	10MR	-	-	-
	PreCDO	silylation	IEZ-CDO [116]	10MR	Al	-	-
	Al-RUB-36	silylation	Al-COE-4 [117]	10MR	Al	-	-
	RUB-36	silylation	Al-COE-4/Fe [118]	10MR	Al, Fe	-	-
	PLS-1	silylation	IEZ-CDO [115]	10MR	-	-	-
			IEZ-1 [119]	10MR	-	-	-
			APZ-1 [120]	10MR	-	-	-
	PLS-4	silylation	IEZ-PLS-4 [121]	10MR	-	-	-
			APZ-3 [120]	10MR	-	-	-
	ZSM-55	inorganic pillared	[33]	mesopore	-	-	18.0

*^a^* information reported as pore classification (i.e., mesopore), dimension (in Å) and/or pore size (MR).

**Table 4:** Structural properties of 2D layered precursors and their related 3D (10-MR) zeolites.

3D Zeolite Framework (Pore Structure) *^a^* (Å)	2D Zeolite Precursor (Layer Stacking Direction) *^b^*	SDA in 2D Precursor Synthesis	2D Layer Pore Structure *^a^* (Å)	2D Layer Property			
				Q^3^:Q^4^ Ratio *^c^*	d(OH⋯OH) *^d^* (Å)	Layer Thickness (Å)	Inter-Layer Distance (Å)
AFO (10MR: 4.1 × 5.3)	[F, Tet-A]-AlPO-1 [133,134] (b-axis)	TMAOH	6MR 2.2 × 3.0	-	-	-	-
FER (10MR: 4.2 × 5.4 8MR: 3.5 × 4.8)	PREFER [61] (a-axis)	ATMP	5MR 1.0 × 1.8	0.3:1	5.7	9.5	3.6
	PLS-3 [91] (a-axis)	TEAOH	5MR 1.0 × 1.8	0.3:1	1.9	-	11.7
	ICP-2 [137] (a-axis)	DMEP	5MR 1.0 × 1.8	-	-	-	19.8
	ERS-12 [138] (a-axis)	TMAOH	5MR 1.0 × 1.8	-	-	-	10.6
HEU (10MR: 3.1 × 5.5 8MR: 4.1 × 4.1)	CIT-8P [139] (b-axis)	diquaternary imidazoles	5MR 0.9 × 2.2	0.7:1 *^e^*	-	-	12.8 *^f^*
	HUS-2 [96] (b-axis)	choline hydroxide and Na^+^	5MR 0.9 × 2.2	0.6:1	2.6	-	3.6
	HUS-7 [140] (b-axis)	BTMAOH and biphenyl	5MR 0.9 × 2.2	0.7:1	2.4	-	17.3
MFI (10MR: 5.1 × 5.5 10MR: 5.3 × 5.6)	multilamellar MFI [48,141] (b-axis)	C_22-6-6_Br_2_	10MR 5.1 × 5.5 10MR 5.3 × 5.6	0.2:1	2.7	19.7 or 34.0 *^g^*	41.0
		multi-quaternary ammonium	10MR 5.1 × 5.5 10MR 5.3 × 5.6	-	-	20.0–34.0 *^h^*	20.0–60.0 *^i^*
	single-pore thickness MFI [142] (b-axis)	C_18-6-6-18_Br_3_	10MR 5.1 × 5.5 10MR 5.3 × 5.6	-	-	15.0	34.0
	SCZN-1 [143] (b-axis)	C_Ph–Ph-10-6/_C_Nh-10-6_	10MR 5.1 × 5.5 10MR 5.3 × 5.6	-	-	-	-
	Multilamellar TS-1 [144] (b-axis)	C_22-6-6_Br_2_	10MR 5.1 × 5.5 10MR 5.3 × 5.6	-	-	34.0	12.0
MWW (10MR: 4.0 × 5.5 10MR: 4.1 × 5.1)	MCM-22(P) [57,145] (c-axis)	HMI	12MR 7.1 × 18.2 10MR 4.1 × 5.1	0.5:1	8.3	25.1	1.9
	EMM-10P [146,147] (c-axis)	Diquat-C5	12MR 7.1 × 18.2 10MR 4.1 × 5.1	-	-	25.0	>1
	ERB-1 [148,149] (c-axis)	Piperidine	12MR 7.1 × 18.2 10MR 4.1 × 5.1	-	-	-	1.8
	MCM-56 [62,150,151]	HMI	12MR 7.1 × 18.2 10MR 4.1 × 5.1	-	9.9–11.0	25.0	-
	UZM-8 [152]	DEDMAOH	12MR 7.1 × 18.2 10MR 4.1 × 5.1	-	-	-	13.4
	SSZ-70 [153,154,155]	diquaternary imidazoles	12MR 7.1 × 18.2 10MR 4.1 × 5.1	-	-	-	2.0
	IPC-3P [156]	1,4-MPB	12MR 7.1 × 18.2 10MR 4.1 × 5.1	-	-	-	4–12.6
	UJM-1P [157]	Ada-4-16	12MR 7.1 × 18.2 10MR 4.1 × 5.1	-	-	-	26 *^f^*
RRO (10MR: 4.0 × 6.5 8MR: 2.7 × 5.0)	RUB-39 [60] (b-axis)	DMDPAOH	5MR 1.1 × 1.8	0.3:1	7.0	7.8	3.0
	Al-, B-RUB-39 [158] (b-axis)	DMDPA	5MR 1.1 × 1.8	-	-	-	-
STI (10MR: 4.7 × 5.0 8MR: 2.7 × 5.6)	PKU-22 [159] (b-axis)	TEAOH	6MR 0.5 × 2.6	-	2.8	-	10.6 *^f^*

*^a^* reported for micropore opening with sizes greater than 6MR in each framework. *^b^* layer stacking direction for 2D zeolite precursor; *^c^* Q^3^: three-connected [SiO_4_] tetrahedra structure; Q^4^: four-connected [SiO_4_] tetrahedra structure; *^d^* minimum intra-layer distance between terminal silanol (Si-OH) or siloxy (Si-O) groups; *^e^* Q^3^:Q^4^ for ratio for calcined CIT-8P, CIT-8; *^f^* d-spacing distance in 2D zeolite precursor; *^g^* layer thickness is under debate; *^h^* the layer thickness varies with different templates in synthesis; and *^i^* the interlayer distance varies with different templates in synthesis.

**Table 5:** Structural and compositional properties of derivatives of 2D FER, HEU (heulandite), and RRO (RUB-41 (forty-one)) zeolites.

3D Zeolite Framework	2D Zeolite Precursor	Re-Organizing Method	Derivative Structure Property				
			2D Zeolite Derivative	Inter-Layer Pore Formed *^a^*	Layer Heteroatom Composition	Pillar Heteroatom Composition	Inter-Layer Distance (Å)
FER	PREFER	delaminated	ITQ-6 [14,63]	-	Al, Ti	-	-
			UCB-2 [162]	-	Al	-	-
		inorganic pillared	ITQ-36 [34]	mesopore	Al, Ge, Ti	Ge, Ti, Al, B, Fe, Cr, Ga	27.5
		silylation	IEZ-FER [115,163]	12MR	-	-	-
		silylation	APZ-4 [120]	12MR	-	-	-
	ZSM-55	inorganic pillared	Pillared FER [33]	mesopore	B	-	25.0
	PLS-3	silylation	IEZ-Sn-PLS-3 [165]	12MR	-	Sn	-
		silylation	IEZ-PLS-3 [163]	12MR	Al	-	-
		silylation	ECNU-9 [164]	14MR	Ti	-	-
		silylation	APZ-2 [120]	12MR	-	-	-
HEU	HUS-2	silylation	HUS-10 [166]	12MR	-	-	-
			Ti*_x_*-HUS [167]	12MR	-	Ti	-
RRO	RUB-39	silylation	COE-1 [168]	12MR	-	-	-
		silylation	RUB-39 DCDMS/HMDS [169]	12MR	Al	-	-

*^a^* information reported as pore classification (i.e., mesopore), dimension (in Å) and/or pore ring size (MR).

**Table 6:** Structural and compositional properties of derivatives of 2D MFI zeolite.

3D Zeolite Framework	2D Zeolite Precursor	Re-Organizing Method	Derivative Structure Property				
			2D Zeolite Derivative	Inter-Layer Pore Formed *^a^*	Layer Heteroatom Composition	Pillar Heteroatom Composition	Inter-Layer Distance (Å)
MFI	multilamellar MFI	delaminated	exfoliated MFI [18,19,20]	-	Al	-	-
		inorganic pillared	pillared MFI [35]	mesopore	Al	-	41.0
			titanosilicate pillared MFI [42]	mesopore	Al	Ti	23.0
			tin–silica pillared MFI [176]	mesopore	-	Sn	31.9
		organic pillared	BTEB pillared MFI [36]	-	Al	BTEB	12.6
	multilamellar TS-1	inorganic pillared	pillared TS-1, Ti-pillared TS-1 [177]	mesopore	Ti	Ti	31.9
			P-TS-1 with long-range order [144]	mesopore	Ti	-	28.0
	direct synthesis	unilamellar	MFI nanosheet agglomeration [178]	-	Al	-	-
			MFI nanosheet [171]	-	-	-	-
			TS-1 nanosheet agglomeration [172]	-	Ti	-	-
		inorganic pillared	self-pillared pentasil [173]	mesopore	Al, Sn	Al	20.0–70.0
			SCZN-2 [143]	mesopore	Al	Al	16.7–28.2
			MZIN [174]	mesopore	Al	Al	20.0–40.0

*^a^* information reported as pore classification (i.e., mesopore), dimension (in Å) and/or pore ring size (MR).

**Table 7:** Structural and compositional properties of derivatives of 2D MWW zeolite.

3D Zeolite Framework	2D Zeolite Precursor	Derivative Structure Property					
		Re-Organizing Method	2D Zeolite Derivative	Inter-Layer Pore Formed *^a^*	Layer Heteroatom Composition	Pillar Heteroatom Composition	Inter-Layer Distance (Å)
MWW	MCM-22(P)	detemplated	[46]	-	Al	-	-
		delaminated	ITQ-2 [13,187], Ti-ITQ-2 [188]	-	Al, Ti	-	-
			UCB-1 [22]	-	Al	-	-
			exfoliated MCM-22(S) [20]	-	Al	-	-
			swollen MCM-22(P) [21] extrusion	-	Al	-	-
		inorganic pillared	MCM-36 [25]	30.0 Å-35.0 Å	Al	-	>24.9
			Al_2_O_3_-MCM-36, MgO-Al_2_O_3_-MCM-36, BaO-Al_2_O_3_-MCM-36 [27,28]	mesopore	Al	Al, Mg, Ba	5.0–24.9
			Ti-MCM-36, Si/Ti-MCM-36 [189,190,191]	mesopore	Al	Ti	14.9–18.9
		organic pillared	MCM-22(PS-RT) [21]	-	Al	-	16.9
			MWW-BTEB [29]	-	Al	-	15.1
	direct synthesis	unilamellar	DS-ITQ-2 [192]	-	Al	-	-
			MIT-1 [67]	-	Al	-	-
	MCM-56	delaminated	[182]	-	Al	-	-
		pillared	[182]	mesopore	Al, Sn, B	-	45.1 ^*b*^
	ERB-1P	delaminated	ERB-1-del-135 [23]	-	Al	-	-
		inorganic pillared	Si/Ti oxide pillared MCM-36 [190]	-	Al	Ti	45.1 *^b^*
		silylation	IEZ-MWW [115,193]	12MR	Al, Ce	-	-
			Ti-YNU-1 [194,195]	12MR	Al	-	-
	SSZ-70	delaminated	UCB-3, UCB-4 [183]	-	Al, B	-	-

*^a^* information reported as pore classification (i.e., mesopore), dimension (in Å) and/or pore ring size (MR); *^b^* d-spacing distance.

**Table 8:** Structural and compositional properties of 2D zeolite layers, derivatives, and 3D parent zeolites practiced in the ADOR (assembly–disassembly–organization–reassembly) process.

3D Parent Zeolite (Pore Structure) *^a^* (Å)	2D Zeolite Precursor (Layer Stacking Direction) *^b^*	Re-Organizing Method	Derivative Structure Property					
			2D Zeolite Derivative	Inter-Layer Connection Unit	Inter-Layer Pore Dimension	Layer Heteroatom Composition	Pillar Heteroatom Composition	d-Spacing (Å)
UTL (14MR: 9.5 × 7.1 12MR: 8.5 × 5.5)	IPC-1P [199] (c-axis)	direct calcination	IPC-1 [199,200]	oxygen *^c^*	sub-zeolite *^f^*	Ge, B	--	9.0
		silylation in acid solution	IPC-2 [199] (OKO)	s4R *^d^*	12 and 10MR	Ge, Ti	-	11.5
		octylamine intercalation	IPC-4 [66] (PCR)	oxygen	10 and 8MR	Ge, Ti	-	-
		staged de-intercalation	IPC-6 [201] (*PCS)	oxygen and s4R	12, 10 and 8MR	Ge	-	-
		staged de-intercalation	IPC-7 [201]	d4R *^e^* and s4R	14, 12 and 10 MR	Ge	-	-
		choline intercalation	IPC-9 [202]	oxygen	10 and 7MR	Ge	-	-
		choline and organosilane intercalation	IPC-10 [202]	s4R	12 and 9MR	Ge	-	-
		swelling	IPC-1SW [31,203,204]	organic	mesopore	Ge	-	10.4–39.0
		inorganic pillaring	IPC-1PI [31,203]	SiO_2_	mesopore	Ge,	-	38.0
			B-IPC-1PI [31]	SiO_2_	mesopore	Ge, B	-	42.0
			Fe-IPC-1PI [31]	SiO_2_	mesopore	Ge, Fe	-	44.1
			Ti-IPC-1PISi [205] Ti-IPC-1PITi	SiO_2_ SiO_2_/TiO_2_	mesopore	Ge, Ti	Ti	37.0
IWW (12MR: 6.0 × 6.7 10MR: 4.9 × 4.9)	IPC-5P [65] (c-axis)	direct calcination	IPC-5 [206]	d4R	12, 10 and 8MR	Ge	-	-
		silylation	IWW (siliceous) [65]	d4R	12, 10 and 8MR	-	-	-
		alumination with AlCl_3_	IWW (Al-containing) [65]	d4R	12, 10 and 8MR	Ge, Al	-	-
		swelling	IPC-5SW [65]	organic	mesopore	Ge	-	-
UOV (12MR: 6.0 × 7.7 12MR: 5.9 × 7.1; 10MR: 4.7 × 5.9)	IPC-12P [207,208] (a-axis)	direct calcination	IPC-12 [207,208]	oxygen	12 and 8MR	Ge	-	-
SAZ-1	SAZ-1P [209] (a-axis)	octylamine intercalation	IPC-15 [209]	oxygen	-	-	-	8.4
		silylation in acid solution	IPC-16 [209]	s4R	-	-	-	10.2

*^a^* reported for the micropore openings (i.e., 14 and 12MR) in each zeolite. *^b^* layer stacking direction for 2D zeolite precursor; *^c^* linkage between layers is oxygen atom; *^d^* linkage between layers is single four-ring units (S4R); *^e^* linkage between layers is double four-ring units (D4R); *^f^* layers are partially connected and partially collapsed in sub-zeolite.

**Table 9:** Structural and compositional properties of other types of 2D zeolite structures.

3D Zeolite Framework (Pore Structure) *^a^* (Å)	2D Zeolite Properties				
	2D Zeolite Structure (Layer Stacking Direction) *^b^*	SDA Used in Synthesis	Particle Morphology	Layer Thickness (Å)	Heteroatom Composition
MEL (10MR: 5.3 × 5.4)	MTS-2 [69]	CTATos, TBAOH	olive-like nanosheet aggregates	50–100	Ti
FAU (12MR: 7.4 × 7.4)	NaX-T-cal [70,71]	TPHAC, Zn(NO_3_)_2_, Li_2_CO_3_	ball-shaped house-of-cards nanoplate assemblies	~70	Al
MOR (12MR: 6.5 × 7.0 8MR: 4.8 × 3.4 8MR: 2.6 × 5.7)	MOR nanoplate [74] (c-axis)	C_16-2-0_	nanoplate aggregates	200–400	Al
	MOR nanoplate [211] (c-axis)	poly-quaternary ammonium	nanoplate aggregates	-	Al
TON (10MR: 4.6 × 5.7)	ZSM-22 [73]	1-ethylpyridinium bromide	nanoplates	80–500	Al
MRE (10MR: 5.6 × 5.6)	LMZN [75] (c-axis)	BPT_n−6−0_	flower-like nanosheet agglomerates	30 *^b^*	-

*^a^* Reported for all micropore openings greater than or equal to 8 MR in each zeolite. *^b^* zeolite layers connected by 40 Å surfactant layers.

**Table 10:** Representative organic base molecules used in acidity determination in 2D zeolites.

Organic Base	Kinetic Diameter (Å)	Accessibility to Acid Sites in Zeolites	Acidity Type
CO	3.8 [231]	>6 MR	Brønsted; Lewis
DME	4.7 [232]	>6 MR	Brønsted
CD_3_CN	4.8 [233]	>6 MR	Brønsted; Lewis
IPA	5.3 [234]	>8 MR	Brønsted
Pyridine	5.4 [235]	>8 MR	Brønsted; Lewis
Pivalonitrile	6.2 [236]	>10 MR	Brønsted
2,6-lutidine	6.7 [235]	>12 MR	Brønsted; Lewis
2,4,6-collidine	7.4 [235]	>12 MR	Brønsted; Lewis
DTBP	7.9 [237]	>12 MR	Brønsted
DMQ	8.3 [238]	>12 MR	Brønsted
TPP	9.4 [239]	>12 MR	Brønsted
TMPO	5.5 [230]	>8 MR	Brønsted; Lewis
TBPO	8.2 [44]	>10 MR	Brønsted; Lewis

**Table 11:** Selected literature values of extra-framework Al and external acid sites in a 2D MFI zeolite probed by different techniques.

Zeolite Material	Extra-Framework Al (%)	External Acid Sites (%)			
	^27^Al MAS NMR *^a^*	FTIR of Adsorbed Base		Base Titration	^31^P MAS NMR
		DTBP	Collidine *^g^*	DTBP *^h^*	TBPO *^j^*
3D MFI	22.0 *^b^*/6.7 *^c^* [248]	4.0 *^e^* [235]	6.3 *^b^*/2.5 *^c^* [248]	3.2 *^i^* [173]	4.7 *^i^* [79]
Unilamellar MFI	-	11.3 *^f^* [247] 50.0 *^e^* [235]	-	-	32.0 [79]
Multilamellar MFI	30.0–46.5 *^d^* [248]	-	19.0–45.9 [248]	-	-
Pillared MFI	16.0 [245]	-	-	28.7 [173]	-
SPP	-	-	-	40.8 [173]	-

*^a^* calculated by peak area of extra-framework Al/framework Al in ^27^Al MAS NMR spectra; *^b^* catalyst from Akzo Nobel Catalysts (now Albemarle Catalysts); *^c^* catalyst from AlSiPenta (SüdChemie, now Clariant); *^d^* 2D MFI zeolites synthesized using variant versions of multi-quaternary ammonium templates; *^e^* calculated by acid sites from DTBP adsorption divided by Brønsted acid sites from pyridine adsorption; *^f^* calculated by acid sites from DTBP adsorption divided by acid sites from pyridine adsorption; *^g^* evaluated by acid sites from collidine adsorption divided by acid sites from pyridine adsorption; *^h^* obtained from DTBP uptake measurement; *^i^* catalyst is from Zeolyst (CBV8014); and *^j^* calculated from acid sites from TBPO signal divided by acid sites from TMPO signal in ^31^P MAS NMR measurement.

**Table 12:** Selected literature values of acid site concentration and strength for 2D MWW zeolites evaluated by the FTIR of adsorbed pyridine after desorption at different temperature conditions.

Ref. #	Zeolite Material	Si/Al Ratio	Acidity (µmol g^−1^)						Acid Strength	
			Brønsted			Lewis			523 K/423 K *^e^*	623 K/423 K *^f^*
			423 K	523 K	623 K	423 K	523 K	623 K		
[187,258]	MCM-22	50	39	24	15	23	15	14	0.63	0.47
	MCM-36	- *^a^*	7	5	3	7	6	6	0.79	0.64
	ITQ-2	- *^a^*	21	15	9	23	20	15	0.80	0.55
[62]	MCM-22	50	57	48	33 *^d^*	23	20	20 *^d^*	0.85	0.66 *^g^*
	MCM-56	9	64	59	35 *^d^*	77	25	21 *^d^*	0.60	0.40 *^g^*
	ITQ-2	- *^a^*	21	15	9 *^d^*	23	20	15 *^d^*	0.80	0.55 *^g^*
[260]	MCM-36	54 *^b^*	56	50	33	19	17	17	0.89	0.67
	MCM-36	23 *^b^*	108	98	65	40	31	30	0.87	0.64
	MCM-36	24	62	47	21	61	44	42	0.74	0.51
[267]	MCM-22	50	**-**	208	191	**-**	47	19	**-**	0.82 *^h^*
	MCM-36	28 *^c^*	**-**	87	70	**-**	61	32	**-**	0.69 *^h^*
	MCM-36	50 *^c^*	**-**	57	48	**-**	40	23	**-**	0.73 *^h^*
	MCM-36	100 *^c^*	**-**	36	32	**-**	31	15	**-**	0.70 *^h^*
[268]	MCM-22	15	216	195	157	65	67	66	0.93	0.79
	MCM-36	- *^a^*	214	187	125	81	74	62	0.88	0.63
	ITQ-2	- *^a^*	130	112	78	80	77	65	0.90	0.68

*^a^* unknown Si/Al ratio, but the zeolite material was derived either by pillaring or exfoliation of MCM-22(P) that led to MCM-22 in the same study; *^b^* obtained by swelling MCM-22(P) at room temperature condition; *^c^* Si/Al ratio refers to that of MCM-22(P) that is used to synthesize MCM-36; *^d^* temperature of desorption is 573 K; *^e^* calculated by ratio of total acid sites measured at 523 K to that of at 423 K; *^f^* calculated by ratio of total acid sites measured at 623 K to that of at 423 K; *^g^* calculated by ratio of total acid sites measured at 573 K to that of at 423 K; and *^h^* calculated by the ratio of total acid sites measured at 623 K to that of at 523 K.

**Table 13:** Selected literature values of extra-framework Al and external acid sites in 2D MWW zeolite probed by different techniques.

Zeolite Material		Extra-Framework Al (%)	External Acid Sites (%)				
		^27^Al MAS NMR *^b^*	FTIR of Adsorbed Base			Base Titration	^31^P MAS NMR
			DTBP *^e^*	DTBP *^f^*	Pivalonitrile or DMQ	DTBP *^i^*	TBPO *^j^*
MCM-22		12.8 [28]	1.0 [28,62]	6.5 [187]; 5.1 [264]; 3.0 [235]; 12.1 [156]; 17.8 [269]	29.7 *^g^* [182]; 11.7–14.6 *^h^* [226]	8.0 [245]	13.0 [67]
ITQ-2		-	4.0 [258]; 1.6 [62]	40.0 [187]; 65.0 [235]	-	-	-
MCM-36 *^a^*	Al_2_O_3_	44.9 [28]	1.4 [28]	-	-	-	-
	MgO-Al_2_O_3_	80.7 [28]	1.7 [28]	-	-	-	-
	BaO-Al_2_O_3_	75.6 [28]	1.5 [28]	-	-	-	-
	SiO_2_	29.5 [28]	1.5 [28]	18.4 [264]; 30.0 [156]; 50.0 [269]	42.9–45.7 *^h^* [226]	67.0 [245]	-
	Al_2_O_3_-SiO_2_	30.4 [28]	2.5 [28]	-	-	-	-
	MgO-Al_2_O_3_-SiO_2_	37.7 [28]	2.4 [28]	-	-	-	-
	BaO-Al_2_O_3_-SiO_2_	33.7 [28]	2.3 [28]	-	-	-	-
MCM-56		-	1.3 [62]	43.6 [269]	58.8 *^g^* [182]	-	40.6 [67]
Pillared MCM-56		-	-	55.2 [269]	-	-	-
Delaminated MCM-56		-	-	-	40.9–68.3 *^g^* [182]	-	-
IPC-3		-	-	8.1 [156]	-	-	-
Pillared IPC-3-PI		-	-	50.8 [156]	-	-	-
MIT-1		8.0 *^c^* [67]; 30.0 *^d^* [67]	-	-	-	-	63.6 [67]

*^a^* sub-column on the right shows the pillar material in MCM-36; *^b^* calculated by peak area of extra-framework Al/framework Al in ^27^Al MAS NMR spectra; *^c^* extra-framework Al before calcination; *^d^* extra-framework Al after calcination; *^e^* DTBP adsorbed normalized to that adsorbed on MCM-22; *^f^* acid sites determined from DTBP adsorption divided by Brønsted acid sites measured from pyridine adsorption; *^g^* acid sites determined by adsorbed pivalonitrile divided by Brønsted acid sites measured by pyridine adsorption; *^h^* acid sites determined by adsorbed DMQ divided by that measured by pyridine adsorption; *^i^* obtained from DTBP uptake measurement; and *^j^* calculated from acid sites from TBPO signal divided by acid sites from TMPO signal in ^31^P MAS NMR measurement.

## Abbreviations

[F, Tet-A]-AlPO-1	[**F**luoride, meso-5,7,7,12,14,14-hexamethyl-1,4,8,11-**tet**raazacyclotetradecane]- **Al**uminium **P**h**o**sphate number 1
Ada-4-16	C_10_H_15_–N^+^(CH_3_)_2_–C_4_H_8_–N^+^(CH_3_)_2_–C_16_H_33_
AFO	**A**lPO_4_-41 (**f**orty-**o**ne) (IZA code AFO)
AlPO_4_-41	Aluminophosphate number 41
APZ-1	**A**tom **P**illared **Z**eolitic material number 1
AST	**A**luminophosphate with sequence number **S**ix**T**een (IZA code AST)
ATMP	4-Amino-2,2,6,6-tetramethylpiperidine
BPT_n-6-0_	(C_6_H_2_)_3_–(O–C_n_H_2n_–N^+^(CH_3_)_2_–C_6_H_12_–N(CH_3_)_2_(Br^−^))_6_
BTMAOH	benzyltrimethylammonium hydroxide
Bza-4-16	C_16_H_33_–N^+^(CH_3_)_2_–C_4_H_8_–N^+^(CH_3_)_2_–benzylamine
C_16-2-0_	C_16_H_33_–N^+^(CH_3_)_2_–C_2_H_4_–N(CH_3_)_2_Br
C_18-6-6-18_Br_3_	C_18_H_37_–N^+^(CH_3_)_2_–C_6_H_12_–N^+^(CH_3_)_2_–C_6_H_12_–N^+^(CH_3_)_2_–C_18_H_37_(Br^−^)_3_
C_22-6-6_Br_2_	C_22_H_45_–N^+^(CH_3_)_2_–C_6_H_12_–N^+^(CH_3_)_2_–C_6_H_13_(Br^−^)_2_
C_Nh-10-6_	C_6_H_4_–C_4_H_3_–O–C_10_H_20_–N^+^(CH_3_)_2_–C_6_H_13_(Br^−^)
C_Ph–Ph-10-6_	C_6_H_5_–C_6_H_4_–O–C_10_H_20_–N^+^(CH_3_)_2_–C_6_H_13_(Br^−^)
CAS	**C**esium **A**lumino**S**ilicate (IZA code CAS)
CD_3_CN	Acetonitrile-d3
CDS-1	**C**ylindrically **D**ouble **S**aw-Edged zeolite number 1
CDO	**CD**S-1 (**o**ne) (IZA code CDO)
CIT-8P	**C**alifornia **I**nstitute of **T**echnology number 8 **P**recursor
COE-3	International Network of **C**enters **O**f **E**xcellence number 3
CTAB	cetyltrimethylammonium bromide
CTMA	hexadecyltrimethylammonium
Del-Nu-6	**Del**aminated-**Nu**-6
DEDMAOH	diethyldimethylammonium hydroxide
DLM-2	**D**elft **L**ayered **M**aterial number 2
DMDPA	dimethyldipropylammonium
DMDPAOH	dimethyldipropylammonium hydroxide
DME	dimethyl ether
DMEP	(1R,2S)-dimethylephedrinium
DMF	dimethylformamide
DS-ITQ-2	**D**irect **S**ynthesis **I**nstituto de **T**ecnología **Q**uímica number 2
DTBP	di-tert-butyl peroxide
ENCU-9	**E**ast **C**hina **N**ormal **U**niversity number 9
EMM-10P	**E**xxon**M**obil **M**aterial number 10 **P**recursor
ERB-1P	**E**ni**R**icerche-**B**oralite number 1 **P**recursor
ERS-12	**E**ni**R**icerche molecular **S**ieve number 12
ETMAOH	ethyltrimethylammonium hydroxide
EU-19	**E**dinburgh **U**niversity number 19
FAU	**Fau**jasite (IZA code FAU)
FER	**Fer**rierite (IZA code FER)
HEU	**Heu**landite (IZA code HEU)
HLS	**H**elix **L**ayered **S**ilicate
HMI	hexamethyleneimine
HPM-2	Nanostructured **H**ybrid biohybrid and **P**orous **M**aterials number 2
HUS-1	**H**iroshima **U**niversity **S**ilicate number 1
ICP-2	**I**nstituto de **C**atálisis y **P**etroleoquímica number 2
IEZ	interlayer expanded zeolite
IM-12	**I**nstitut Français du Pétrole and University of **M**ulhouse
IPC-1P	**I**nstitute of **P**hysical **C**hemistry number 1 **P**recursor
IPC-4	**I**nstitute of **P**hysical **C**hemistry number 4
ITH	**I**nstituto de Tecnologia Quimica Valencia—**th**irteen (IZA code ITH)
ITR	**I**nstituto de Tecnologia Quimica Valencia—**t**hirty-fou**r** (IZA code ITR)
ITQ-2	**I**nstituto de **T**ecnología **Q**uímica number 2
IWR	**I**nstituto de Tecnologia Quimica Valencia—t**w**enty-fou**r** (IZA code IWR)
IWW	**I**TQ-22 (t**w**enty-t**w**o) (IZA code IWW)
LDS	**L**ower **D**imensional **S**ilicate
MCM-22	**M**obil **C**omposition of **M**atter number 22
MCM-22(P)	**M**obil **C**omposition of **M**atter number 22 (**P**recusor)
MCM-22(P)-sw	**M**obil **C**omposition of **M**atter number 22 (**P**recusor) **sw**ollen
MEL	ZS**M**-11 (**el**even) (IZA code MEL)
MFI	ZS**M**-5 (**fi**ve) (IZA code MFI)
MIT-1	**M**assachusetts **I**nstitute of **T**echnology number 1
MOR	**Mor**denite (IZA code MOR)
MRE	ZS**M**-48 (fo**r**ty-**e**ight) (IZA code MRE)
MTEAOH	methyltriethylammonium hydroxide
MTF	**M**CM-35 (**t**hirty-**f**ive) (IZA code MTF)
MTS-2	**M**ultilayered **T**itanium **S**ilicalite-2
MWW	**M**CM-22 (t**w**enty-t**w**o) (IZA code MWW)
MZIN	**M**esoporous **Z**SM-5 with **I**ntercrossed **N**anosheets
NSI	**N**u-6(2) (**si**x) (IZA code NSI)
Nu-6(1)	(**New** (ICI, Imperial Chemical Industries) with sequence number Six (one)
OKO	**O**ppervlakte en **K**atalyse **O**ne (IZA code OKO)
PCR	I**PC**-4 (fou**r**) (IZA code PCR)
PKU-22	**P**e**k**ing **U**niversity number 22
PLS-3	**P**entagonal-cylinder **L**ayered **S**ilicate number 3
PREFER	**Pre**cursor of **fer**rierite
RRO	**R**UB-41 (fo**r**ty-**o**ne) (IZA code RRO)
RTH	**R**UB-13 (**th**irteen) (IZA code RTH)
RUB-55	**R**uhr **U**niversity **B**ochum number 55
RWR	**R**UB-24 (t**w**enty-fou**r**) (IZA code RWR)
SAZ-1	University of **S**t. **A**ndrews **Z**eolite—one
SCZN-1	**S**ingle-**C**rystalline mesostructured **Z**eolite **N**anosheets number 1
SFO	**S**SZ-51 (**f**ifty-**o**ne) (IZA code SFO)
SOD	**Sod**alite (IZA code SOD)
SSZ-51	**S**tandard Oil **S**ynthetic **Z**eolite number 51
STI	**Sti**lbite (IZA code STI)
TBPO	tributylphosphine oxide
TEAOH	tetraethylammonium hydroxide
TEOS	tetraethyl orthosilicate
Ti-YNU-1	**Y**okohama **N**ational **U**niversity number 1
TMAOH	tetramethylammonium hydroxide
TMMPBr	tetramethylene bis(N-methylpyrrolidinium) dibromide
TMPOH	tetramethylphosphonium hydroxide
TMPAOH	trimethyl-isopropylammonium hydroxide
TON	**T**heta-1 (**on**e)
TPAOH	tetra-n-propylammonium hydroxide
TPHAC	3-(trimethoxysilyl)propyl hexadecyl dimethyl ammonium chloride
TPP	triphenylphosphine
UCB-1	**U**niversity of **C**alifornia at **B**erkeley number 1
UJM-1P	**U**niwersytet **J**agiellonski **M**aterial #1
UOV	Institut Français du Pétrole and University of M**u**lhouse—seventeen (**o**ne se**v**en) (IZA code UOV)
UTL	IM-12, M**u**lhouse (**t**we**l**ve) (IZA code UTL)
UZM-13	**U**niversal Oil Products **Z**eolitic **M**aterial number 13
ULS-1	**U**OP **L**ayered **S**ilicate-1
ZSM-5	**Z**eolite **S**ocony **M**obil number 5

## References

[1] Weitkamp J. (2000). Zeolites and catalysis. Solid State Ion..

[2] Čejka J., Wichterlová B. (2002). Acid-catalyzed synthesis of mono- and dialkyl benzenes over zeolites: Active sites, zeolite topology, and reaction mechanisms. Catal. Rev..

[3] Eliasova P., Čejka J. (2017). Two-dimensional zeolites. Zeolites in Catalysis: Properties and Applications.

[4] Farneth W.E., Gorte R.J. (1995). Methods for characterizing zeolite acidity. Chem. Rev..

[5] Luo H.Y., Lewis J.D., Román-Leshkov Y. (2016). Lewis acid zeolites for biomass conversion: Perspectives and challenges on reactivity, synthesis, and stability. Annu. Rev. Chem. Biomol..

[6] Roth W.J., Nachtigall P., Morris R.E., Čejka J. (2014). Two-dimensional zeolites: Current status and perspectives. Chem. Rev..

[7] Čejka J., Centi G., Perez-Pariente J., Roth W.J. (2012). Zeolite-based materials for novel catalytic applications: Opportunities, perspectives and open problems. Catal. Today.

[8] Roth W.J., Gil B., Makowski W., Marszalek B., Eliasova P. (2016). Layer like porous materials with hierarchical structure. Chem. Soc. Rev..

[9] Opanasenko M.V., Roth W.J., Čejka J. (2016). Two-dimensional zeolites in catalysis: Current status and perspectives. Catal. Sci. Technol..

[10] Xu L., Sun J. (2016). Recent advances in the synthesis and application of two-dimensional zeolites. Adv. Energy Mater..

[11] Díaz U., Corma A. (2014). Layered zeolitic materials: An approach to designing versatile functional solids. Dalton Trans..

[12] Roth W.J., Gil B., Marszalek B. (2014). Comprehensive system integrating 3D and 2D zeolite structures with recent new types of layered geometries. Catal. Today.

[13] Corma A., Fornes V., Pergher S.B., Maesen T.L.M., Buglass J.G. (1998). Delaminated zeolite precursors as selective acidic catalysts. Nature.

[14] Corma A., Diaz U., Domine M.E., Fornés V. (2000). AlITQ-6 and TiITQ-6: Synthesis, characterization, and catalytic activity. Angew. Chem. Int. Ed..

[15] Corma A., Fornes V., Diaz U. (2001). ITQ-18 a new delaminated stable zeolite. Chem. Commun..

[16] Osada S., Iribe A., Kuroda K. (2013). Exfoliation of layered octosilicate by simple cation exchange with didecyldimethylammonium ions. Chem. Lett..

[17] Gorgojo P., Galve A., Uriel S., Téllez C., Coronas J. (2011). Direct exfoliation of layered zeolite Nu-6(1). Microporous Mesoporous Mater..

[18] Varoon K., Zhang X., Elyassi B., Brewer D.D., Gettel M., Kumar S., Lee J.A., Maheshwari S., Mittal A., Sung C.-Y. (2011). Dispersible exfoliated zeolite nanosheets and their application as a selective membrane. Science.

[19] Zhang H., Xiao Q., Guo X., Li N., Kumar P., Rangnekar N., Jeon Mi Y., Al-Thabaiti S., Narasimharao K., Basahel Sulaiman N. (2016). Open-pore two-dimensional MFI zeolite nanosheets for the fabrication of hydrocarbon-isomer-selective membranes on porous polymer supports. Angew. Chem. Int. Ed..

[20] Sabnis S., Tanna V.A., Li C., Zhu J., Vattipalli V., Nonnenmann S.S., Sheng G., Lai Z., Winter H.H., Fan W. (2017). Exfoliation of two-dimensional zeolites in liquid polybutadienes. Chem. Commun..

[21] Maheshwari S., Jordan E., Kumar S., Bates F.S., Penn R.L., Shantz D.F., Tsapatsis M. (2008). Layer structure preservation during swelling, pillaring, and exfoliation of a zeolite precursor. J. Am. Chem. Soc..

[22] Ogino I., Nigra M.M., Hwang S.-J., Ha J.-M., Rea T., Zones S.I., Katz A. (2011). Delamination of layered zeolite precursors under mild conditions: Synthesis of UCB-1 via fluoride/chloride anion-promoted exfoliation. J. Am. Chem. Soc..

[23] Ouyang X., Hwang S.-J., Runnebaum R.C., Xie D., Wanglee Y.-J., Rea T., Zones S.I., Katz A. (2014). Single-step delamination of a MWW borosilicate layered zeolite precursor under mild conditions without surfactant and sonication. J. Am. Chem. Soc..

[24] Takahashi N., Hata H., Kuroda K. (2011). Exfoliation of layered silicates through immobilization of imidazolium groups. Chem. Mater..

[25] Roth W.J., Kresge C.T., Vartuli J.C., Leonowicz M.E., Fung A.S., McCullen S.B., Beyer H.K., Karge H.G., Kiricsi I., Nagy J.B. (1995). MCM-36: The first pillared molecular sieve with zeolite properties. Studies in Surface Science and Catalysis.

[26] He Y.J., Nivarthy G.S., Eder F., Seshan K., Lercher J.A. (1998). Synthesis, characterization and catalytic activity of the pillared molecular sieve MCM-36. Microporous Mesoporous Mater..

[27] Barth J.O., Kornatowski J., Lercher J.A. (2002). Synthesis of new MCM-36 derivatives pillared with alumina or magnesia–alumina. J. Mater. Chem..

[28] Barth J.O., Jentys A., Kornatowski J., Lercher J.A. (2004). Control of acid-base properties of new nanocomposite derivatives of MCM-36 by mixed oxide pillaring. Chem. Mater..

[29] Corma A., Díaz U., García T., Sastre G., Velty A. (2010). Multifunctional hybrid organic–inorganic catalytic materials with a hierarchical system of well-defined micro- and mesopores. J. Am. Chem. Soc..

[30] Macedo T.R., Airoldi C. (2010). Organofunctionalized RUB-18 from the intercalated precursor cetyltrimethylammonium cation. Microporous Mesoporous Mater..

[31] Chlubná P., Roth W.J., Greer H.F., Zhou W., Shvets O., Zukal A., Čejka J., Morris R.E. (2013). 3D to 2D routes to ultrathin and expanded zeolitic materials. Chem. Mater..

[32] Roth W.J., Kresge C.T. (2011). Intercalation chemistry of NU-6(1), the layered precursor to zeolite NSI, leading to the pillared zeolite MCM-39(Si). Microporous Mesoporous Mater..

[33] Roth W.J., Gil B., Mayoral A., Grzybek J., Korzeniowska A., Kubů M., Makowski W., Čejka J., Olejniczak Z., Mazur M. (2018). Pillaring of layered zeolite precursors with ferrierite topology leading to unusual molecular sieves on the micro/mesoporous border. Dalton Trans..

[34] Lara A.C., Canós A.C., Segúi V.F., Morales U.D. (2003). Acid Oxide with Micro and Mesoporous Characteristics: ITQ-36. U.S. Patent.

[35] Na K., Choi M., Park W., Sakamoto Y., Terasaki O., Ryoo R. (2010). Pillared MFI zeolite nanosheets of a single-unit-cell thickness. J. Am. Chem. Soc..

[36] Liu B., Wattanaprayoon C., Oh S.C., Emdadi L., Liu D. (2015). Synthesis of organic pillared MFI zeolite as bifunctional acid–base catalyst. Chem. Mater..

[37] Lee H.W., Park S.H., Jeon J.-K., Ryoo R., Kim W., Suh D.J., Park Y.-K. (2014). Upgrading of bio-oil derived from biomass constituents over hierarchical unilamellar mesoporous MFI nanosheets. Catal. Today.

[38] Wu Y., Emdadi L., Wang Z., Fan W., Liu D. (2014). Textural and catalytic properties of Mo loaded hierarchical meso-/microporous lamellar MFI and MWW zeolites for direct methane conversion. Appl. Catal. A.

[39] Ren L., Guo Q., Orazov M., Xu D., Politi D., Kumar P., Alhassan S.M., Mkhoyan K.A., Sidiras D., Davis M.E. (2016). Pillared Sn-MWW prepared by a solid-state-exchange method and its use as a Lewis acid catalyst. ChemCatChem.

[40] Ren L., Guo Q., Kumar P., Orazov M., Xu D., Alhassan S.M., Mkhoyan K.A., Davis M.E., Tsapatsis M. (2015). Self-pillared, single-unit-cell Sn-MFI zeolite nanosheets and their use for glucose and lactose isomerization. Angew. Chem. Int. Ed..

[41] Lima S., Pillinger M., Valente A.A. (2008). Dehydration of d-xylose into furfural catalysed by solid acids derived from the layered zeolite Nu-6(1). Catal. Commun..

[42] Emdadi L., Tran D.T., Zhang J., Wu W., Song H., Gan Q., Liu D. (2017). Synthesis of titanosilicate pillared MFI zeolite as an efficient photocatalyst. RSC Adv..

[43] Přech J., Pizarro P., Serrano D.P., Čejka J. (2018). From 3D to 2D zeolite catalytic materials. Chem. Soc. Rev..

[44] Zhao Q., Chen W.-H., Huang S.-J., Wu Y.-C., Lee H.-K., Liu S.-B. (2002). Discernment and quantification of internal and external acid sites on zeolites. J. Phys. Chem. B.

[45] Thibault-Starzyk F., Stan I., Abelló S., Bonilla A., Thomas K., Fernandez C., Gilson J.-P., Pérez-Ramírez J. (2009). Quantification of enhanced acid site accessibility in hierarchical zeolites—The accessibility index. J. Catal..

[46] Roth W.J., Čejka J., van Bekkum H., Corma A., Schüth F. (2007). Synthesis of delaminated and pillared zeolitic materials. Studies in Surface Science and Catalysis.

[47] Iler R.K. (1964). Ion exchange properties of a crystalline hydrated silica. J. Colloid Sci..

[48] Choi M., Na K., Kim J., Sakamoto Y., Terasaki O., Ryoo R. (2009). Stable single-unit-cell nanosheets of zeolite MFI as active and long-lived catalysts. Nature.

[49] Eliášová P., Opanasenko M., Wheatley P.S., Shamzhy M., Mazur M., Nachtigall P., Roth W.J., Morris R.E., Čejka J. (2015). The ADOR mechanism for the synthesis of new zeolites. Chem. Soc. Rev..

[50] Vortmann S., Rius J., Siegmann S., Gies H. (1997). Ab initio structure solution from X-ray powder data at moderate resolution: Crystal structure of a microporous layer silicate. J. Phys. Chem. B.

[51] Marler B., Stroter N., Gies H. (2005). The structure of the new pure silica zeolite RUB-24, Si_32_O_64_, obtained by topotactic condensation of the intercalated layer silicate RUB-18. Microporous Mesoporous Mater..

[52] Akiyama Y., Mizukami F., Kiyozumi Y., Maeda K., Izutsu H., Sakaguchi K. (1999). A novel layered silicate with a helical morphology. Angew. Chem. Int. Ed..

[53] Ikeda T., Akiyama Y., Izumi F., Kiyozumi Y., Mizukami F., Kodaira T. (2001). Crystal structure of a helix layered silicate containing tetramethylammonium ions in sodalite-like cages. Chem. Mater..

[54] Asakura Y., Takayama R., Shibue T., Kuroda K. (2014). Topotactic conversion of β-Helix-Layered silicate into AST-type zeolite through successive interlayer modifications. Chem. Eur. J..

[55] Marler B., Gies H. (2012). Hydrous layer silicates as precursors for zeolites obtained through topotactic condensation: A review. Eur. J. Mineral..

[56] Whittam T.V. (1983). Zeolites Nu-6(1) and Nu-6(2). U.S. Patent.

[57] Leonowicz M.E., Lawton J.A., Lawton S.L., Rubin M.K. (1994). MCM-22: A molecular sieve with two independent multidimensional channel systems. Science.

[58] Blake A.J., Franklin K.R., Lowe B.M. (1988). Preparation and properties of piperazine silicate (EU-19) and a silica polymorph (EU-20). J. Am. Chem. Soc. Dalton Trans..

[59] Moteki T., Chaikittisilp W., Shimojima A., Okubo T. (2008). Silica sodalite without occluded organic matters by topotactic conversion of lamellar precursor. J. Am. Chem. Soc..

[60] Wang Y.X., Gies H., Lin J.H. (2007). Crystal structure of the new layer silicate RUB-39 and its topotactic condensation to a microporous zeolite with framework type RRO. Chem. Mater..

[61] Schreyeck L., Caullet P., Mougenel J.C., Guth J.L., Marler B. (1996). PREFER: A new layered (alumino) silicate precursor of FER-type zeolite. Microporous Mater..

[62] Corma A., Diaz U., Fornes V., Guil J.M., Martinez-Triguero J., Creyghton E.J. (2000). Characterization and catalytic activity of MCM-22 and MCM-56 compared with ITQ-2. J. Catal..

[63] Chica A., Diaz U., Fornes V., Corma A. (2009). Changing the hydroisomerization to hydrocracking ratio of long chain alkanes by varying the level of delamination in zeolitic (ITQ-6) materials. Catal. Today.

[64] Corma A., Diaz U., Domine M.E., Fornés V. (2000). New aluminosilicate and titanosilicate delaminated materials active for acid catalysis, and oxidation reactions using H_2_O_2_. J. Am. Chem. Soc..

[65] Chlubna-Eliasova P., Tian Y.Y., Pinar A.B., Kubů M., Čejka J., Morris R.E. (2014). The assembly-disassembly-organization-reassembly mechanism for 3D-2D-3D transformation of germanosilicate IWW zeolite. Angew. Chem. Int. Ed..

[66] Roth W.J., Nachtigall P., Morris R.E., Wheatley P.S., Seymour V.R., Ashbrook S.E., Chlubna P., Grajciar L., Polozij M., Zukal A. (2013). A family of zeolites with controlled pore size prepared using a top-down method. Nat. Chem..

[67] Luo H.Y., Michaelis V.K., Hodges S., Griffin R.G., Roman-Leshkov Y. (2015). One-pot synthesis of MWW zeolite nanosheets using a rationally designed organic structure-directing agent. Chem. Sci..

[68] Cho H.J., Ren L., Vattipalli V., Yeh Y.-H., Gould N., Xu B., Gorte R.J., Lobo R., Dauenhauer P.J., Tsapatsis M. (2017). Renewable p-xylene from 2,5-dimethylfuran and ethylene using phosphorus-containing zeolite catalysts. ChemCatChem.

[69] Chen H.L., Li S.W., Wang Y.M. (2015). Synthesis and catalytic properties of multilayered MEL-type titanosilicate nanosheets. J. Mater. Chem. A.

[70] Inayat A., Knoke I., Spiecker E., Schwieger W. (2012). Assemblies of mesoporous FAU-type zeolite nanosheets. Angew. Chem. Int. Ed..

[71] Inayat A., Schneider C., Schwieger W. (2015). Organic-free synthesis of layer-like FAU-type zeolites. Chem. Commun..

[72] Ferdov S. (2017). FAU-type zeolite nanosheets from additives-free system. Microporous Mesoporous Mater..

[73] Luo Y., Wang Z., Jin S., Sun H., Yuan X., Yang W. (2016). Synthesis and crystal growth mechanism of ZSM-22 zeolite nanosheet. CrystEngComm.

[74] Ma M., Huang X., Zhan E., Zhou Y., Xue H., Shen W. (2017). Synthesis of mordenite nanosheets with shortened channel lengths and enhanced catalytic activity. J. Mater. Chem. A.

[75] Zhang Y., Ma Y., Che S. (2018). Synthesis of lamellar mesostructured ZSM-48 nanosheets. Chem. Mater..

[76] Díaz U. (2012). Layered materials with catalytic applications: Pillared and delaminated zeolites from MWW precursors. ISRN Chem. Eng..

[77] Tsapatsis M. (2014). 2-Dimensional zeolites. AICHE J..

[78] Burton A., Accardi R.J., Lobo R.F., Falcioni M., Deem M.W. (2000). MCM-47: A highly crystalline silicate composed of hydrogen-bonded ferrierite layers. Chem. Mater..

[79] Seo Y., Cho K., Jung Y., Ryoo R. (2013). Characterization of the surface acidity of MFI zeolite nanosheets by ^31^P NMR of adsorbed phosphine oxides and catalytic cracking of decalin. ACS Catal..

[80] Ikeda T., Oumi Y., Honda K., Sano T., Momma K., Izumi F. (2011). Synthesis and crystal structure of a layered silicate HUS-1 with a halved sodalite-cage topology. Inorg. Chem..

[81] Tsunoji N., Fukuda M., Yoshida K., Sasaki Y., Ikeda T., Ide Y., Sadakane M., Sano T. (2013). Characterization of layered silicate HUS-5 and formation of novel nanoporous silica through transformation of HUS-5 ion-exchanged with alkylammonium cations. J. Mater. Chem. A.

[82] Marler B., Grünewald-Lüke A., Grabowski S., Gies H. (2012). RUB-55, a new hydrous layer silicate with silicate layers known as motives of the sodalite and octadecasil frameworks: Synthesis and crystal structure. Z. Krist. Cryst. Mater..

[83] Li Z., Marler B., Gies H. (2008). A new layered silicate with structural motives of silicate zeolites: Synthesis, crystals structure, and properties. Chem. Mater..

[84] Massüger L., Baerlocher C., McCusker L.B., Zwijnenburg M.A. (2007). Synthesis and structure analysis of the layer silicate DLM-2. Microporous Mesoporous Mater..

[85] Ide Y., Torii M., Tsunoji N., Sadakane M., Sano T. (2012). Molecular recognitive adsorption of aqueous tetramethylammonium on the organic derivative of Hiroshima University Silicate-1 with a silane coupling reagent. Chem. Commun..

[86] Oberhagemann U., Bayat P., Marler B., Gies H., Rius J. (1996). A layer silicate: Synthesis and structure of the zeolite precursor RUB-15—[N(CH_3_)_4_]_8_[Si_24_O_52_(OH)_4_]·20·H_2_O. Angew. Chem. Int. Ed..

[87] Miller M.A., Miller S.R., Broach R.W., Galey M.M., Prabhakar S., Lyons B., Nicholas C.L., Nicholas C.P. (2015). Synthesis, characterization and structure solution of ULS-1 |ETMA_8_(H_2_O)_20_|[Si_24_O_48_(OH)_8_], a layered silicate composed of half-sodalite cages. Microporous Mesoporous Mater..

[88] Marler B., Camblor M.A., Gies H. (2006). The disordered structure of silica zeolite EU-20b, obtained by topotactic condensation of the piperazinium containing layer silicate EU-19. Microporous Mesoporous Mater..

[89] Rollmann L.D., Schlenker J.L., Lawton S.L., Kennedy C.L., Kennedy G.J. (2002). MCM-69, a novel layered analogue of EU-19. Microporous Mesoporous Mater..

[90] Zanardi S., Alberti A., Cruciani G., Corma A., Fornés V., Brunelli M. (2004). Crystal structure determination of zeolite Nu-6(2) and Its layered precursor Nu-6(1). Angew. Chem. Int. Ed..

[91] Ikeda T., Kayamori S., Mizukami F. (2009). Synthesis and crystal structure of layered silicate PLS-3 and PLS-4 as a topotactic zeolite precursor. J. Mater. Chem..

[92] Ikeda T., Akiyama Y., Oumi Y., Kawai A., Mizukami F. (2004). The topotactic conversion of a novel layered silicate into a new framework zeolite. Angew. Chem. Int. Ed..

[93] Marler B., Wang Y., Song J., Gies H. (2014). Topotactic condensation of layer silicates with ferrierite-type layers forming porous tectosilicates. Dalton Trans..

[94] Dorset D.L., Kennedy G.J. (2004). Crystal structure of MCM-65: An alternative linkage of ferrierite layers. J. Phys. Chem. B.

[95] Knight L.M., Miller M.A., Koster S.C., Gatter M.G., Benin A.I., Willis R.R., Lewis G.J., Broach R.W., Xu R., Gao Z., Chen J., Yan W. (2007). UZM-13, UZM-17, UZM-19 and UZM-25: Synthesis and structure of new layered precursors and a zeolite discovered via combinatorial chemistry techniques. Studies in Surface Science and Catalysis.

[96] Tsunoji N., Ikeda T., Ide Y., Sadakane M., Sano T. (2012). Synthesis and characteristics of novel layered silicates HUS-2 and HUS-3 derived from a SiO_2_-choline hydroxide-NaOH-H_2_O system. J. Mater. Chem..

[97] Rubin M.K. (1991). Crystalline borosilicate. U.S. Patent.

[98] Roth W.J., Gil B., Makowski W., Sławek A., Grzybek J., Kubů M., Čejka J. (2016). Interconversion of the CDO layered precursor ZSM-55 between FER and CDO frameworks by controlled deswelling and reassembly. Chem. Mater..

[99] Chu P., Herbst J.A., Klocke D.J., Vartuli J. (1991). Crystalline aluminosilicate. U.S. Patent.

[100] Rojas A., Camblor M.A. (2014). HPM-2, the layered precursor to zeolite MTF. Chem. Mater..

[101] Schmidt J.E., Xie D., Davis M.E. (2015). Synthesis of the RTH-type layer: The first small-pore, two dimensional layered zeolite precursor. Chem. Sci..

[102] Brenn U., Ernst H., Freude D., Herrmann R., Jähnig R., Karge H.G., Kärger J., König T., Mädler B., Pingel U.T. (2000). Synthesis and characterization of the layered sodium silicate ilerite. Microporous Mesoporous Mater..

[103] Borowski M., Wolf I., Gies H. (2002). Investigation of proton dynamics within the hydrogen-bond network of the layer silicate Na–RUB-18. Chem. Mater..

[104] Kresge C.T., Roth W.J. (1993). Crystalline oxide material. U.S. Patent.

[105] Zubowa H.-L., Schneider M., Schreier E., Eckelt R., Richter M., Fricke R. (2008). The influence of the expanding and exfoliating conditions on the structural transformation of the layered zeolite Nu-6(1). Microporous Mesoporous Mater..

[106] De Pietre M.K., Bonk F.A., Rettori C., Garcia F.A., Pastore H.O. (2012). Delaminated vanadoaluminosilicate with [V,Al]-ITQ-18 structure. Microporous Mesoporous Mater..

[107] Xu H., Jia L., Wu H., Yang B., Wu P. (2014). Structural diversity of lamellar zeolite Nu-6(1)—Postsynthesis of delaminated analogues. Dalton Trans..

[108] Jiang J.-G., Jia L., Yang B., Xu H., Wu P. (2013). Preparation of interlayer-expanded zeolite from lamellar precursor Nu-6(1) by silylation. Chem. Mater..

[109] Borbély G., Beyer H.K., Karge H.G., Schwieger W., Brandt A., Bergk K.H. (1991). Chemical characterization, structural features, and thermal behavior of sodium and hydrogen octosilicate. Clays Clay Miner..

[110] Kosuge K., Tsunashima A. (1995). New silica-pillared material prepared from the layered silicic acid of ilerite. J. Am. Chem. Soc. Chem. Commun..

[111] Kim M., Ko Y., Kim S., Uh Y. (2001). Vapor phase Beckmann rearrangement of cyclohexanone oxime over metal pillared ilerite. Appl. Catal. A.

[112] Kosuge K., Singh P.S. (2000). Mixed-oxide pillared silicates from H-Ilerite by intercalation. Chem. Mater..

[113] Ishii R., Shinohara Y. (2005). Preparation of a microporous biphenyl-pillared layered hybrid material using organic-bridged alkoxysilane and layered silicic acid. J. Mater. Chem..

[114] Ishii R., Ikeda T., Mizukami F. (2009). Preparation of a microporous layered organic–inorganic hybrid nanocomposite using p-aminotrimethoxysilane and a crystalline layered silicate, ilerite. J. Colloid Interface Sci..

[115] Wu P., Ruan J., Wang L., Wu L., Wang Y., Liu Y., Fan W., He M., Terasaki O., Tatsumi T. (2008). Methodology for synthesizing crystalline metallosilicates with expanded pore windows through molecular alkoxysilylation of zeolitic lamellar precursors. J. Am. Chem. Soc..

[116] Martínez-Franco R., Paris C., Martínez-Triguero J., Moliner M., Corma A. (2017). Direct synthesis of the aluminosilicate form of the small pore CDO zeolite with novel OSDAs and the expanded polymorphs. Microporous Mesoporous Mater..

[117] Yilmaz B., Müller U., Feyen M., Zhang H., Xiao F.-S., De Baerdemaeker T., Tijsebaert B., Jacobs P., De Vos D., Zhang W. (2012). New zeolite Al-COE-4: Reaching highly shape-selective catalytic performance through interlayer expansion. Chem. Commun..

[118] De Baerdemaeker T., Gies H., Yilmaz B., Müller U., Feyen M., Xiao F.-S., Zhang W., Yokoi T., Bao X., De Vos D.E. (2014). A new class of solid Lewis acid catalysts based on interlayer expansion of layered silicates of the RUB-36 type with heteroatoms. J. Mater. Chem. A.

[119] Inagaki S., Yokoi T., Kubota Y., Tatsumi T. (2007). Unique adsorption properties of organic–inorganic hybrid zeolite IEZ-1 with dimethylsilylene moieties. Chem. Commun..

[120] Ikeda T., Kayamori S., Oumi Y., Mizukami F. (2010). Structure analysis of Si-atom pillared lamellar silicates having micropore structure by powder X-ray diffraction. J. Phys. Chem. C.

[121] Xu H., Yang B., Jiang J.-G., Jia L., He M., Wu P. (2013). Post-synthesis and adsorption properties of interlayer-expanded PLS-4 zeolite. Microporous Mesoporous Mater..

[122] Kresge C.T., Casmer S.G., Dhingra S. (2002). Crystalline molecular sieve composition MCM-65, its synthesis and use.

[123] Komura K., Murase T., Sugi Y., Koketsu M. (2010). Synthesis of boron-containing CDS-1 zeolite by topotactic dehydration–condensation of [B]-PLS-1 prepared from layered silicate H-LDS. Chem. Lett..

[124] Murase T., Komura K. (2015). Synthesis of germanosilicate type CDS-1 zeolite with CDO topology and its zeolitic layered precursor. J. Porous Mater..

[125] Gies H., Müller U., Yilmaz B., Feyen M., Tatsumi T., Imai H., Zhang H., Xie B., Xiao F.-S., Bao X. (2012). Interlayer expansion of the hydrous layer silicate RUB-36 to a functionalized, microporous framework silicate: Crystal structure analysis and physical and chemical characterization. Chem. Mater..

[126] Lee G.S., Zones S.I. (2002). Polymethylated [4.1.1] octanes leading to zeolite SSZ-50. J. Solid State Chem..

[127] Ikeda T., Oumi Y., Takeoka T., Yokoyama T., Sano T., Hanaoka T. (2008). Preparation and crystal structure of RUB-18 modified for synthesis of zeolite RWR by topotactic conversion. Microporous Mesoporous Mater..

[128] Ishimaru S.I., Togawa M., Ikeda R., Shimizu T., Shinohara E., Umemura Y. (2006). Complex impedance and solid-state NMR studies on a novel high proton conductor Na-RUB-18. Bull. Chem. Soc. JPN..

[129] Ide Y., Ozaki G., Ogawa M. (2009). Swelling in water of a Layered alkali silicate, octosilicate, modified with a sulfonic acid group. Langmuir.

[130] Ogawa M., Iwata D. (2010). Arrangements of interlayer quaternary ammonium ions in a layered silicate, octosilicate. Cryst. Growth Des..

[131] Kim S., Jung K.-D., Joo O.-S., Kim E., Kang T. (2004). Catalytic performance of metal oxide-loaded Ta-ilerite for vapor phase Beckmann rearrangement of cyclohexanone oxime. Appl. Catal. A.

[132] Kim S., Kim E., Kang T., Jung K.-D., Joo O.-S., Shin C.-H. (2006). Synthesis and characterization of transition metal oxide-pillared materials with mesoporosity from layered silicate ilerite. J. Porous Mater..

[133] Wheatley P.S., Love C.J., Morrison J.J., Shannon I.J., Morris R.E. (2002). Synthesis of two new aluminophosphate based layered materials using Tet-A as a structure-directing agent. J. Mater. Chem..

[134] Wheatley P.S., Morris R.E. (2006). Calcination of a layered aluminofluorophosphate precursor to form the zeolitic AFO framework. J. Mater. Chem..

[135] Guo P., Afeworki M., Cao G., Yun Y., Sun J., Su J., Wan W., Zou X. (2018). Synthesis and structure of a layered fluoroaluminophosphate and its transformation to a three-dimensional zeotype framework. Inorg. Chem..

[136] Roth W.J., Dorset D.L. (2010). The role of symmetry in building up zeolite frameworks from layered zeolite precursors having ferrierite and CAS layers. Struct. Chem..

[137] Gálvez P., Bernardo-Maestro B., Vos E., Díaz I., López-Arbeloa F., Pérez-Pariente J., Gómez-Hortigüela L. (2017). ICP-2: A new hybrid organo-inorganic ferrierite precursor with expanded layers stabilized by π–π stacking interactions. J. Phys. Chem. C.

[138] Millini R., Carluccio L.C., Carati A., Bellussi G., Perego C., Cruciani G., Zanardi S. (2004). ERS-12: A new layered tetramethylammonium silicate composed by ferrierite layers. Microporous Mesoporous Mater..

[139] Schmidt J.E., Xie D., Davis M.E. (2015). High-silica, heulandite-type zeolites prepared by direct synthesis and topotactic condensation. J. Mater. Chem. A.

[140] Tsunoji N., Ikeda T., Sadakane M., Sano T. (2014). Synthesis and characteristics of novel layered silicate HUS-7 using benzyltrimethylammonium hydroxide and its unique and selective phenol adsorption behavior. J. Mater. Chem. A.

[141] Park W., Yu D., Na K., Jelfs K.E., Slater B., Sakamoto Y., Ryoo R. (2011). Hierarchically structure-directing effect of multi-ammonium surfactants for the generation of MFI zeolite nanosheets. Chem. Mater..

[142] Jung J., Jo C., Cho K., Ryoo R. (2012). Zeolite nanosheet of a single-pore thickness generated by a zeolite-structure-directing surfactant. J. Mater. Chem..

[143] Xu D., Ma Y., Jing Z., Han L., Singh B., Feng J., Shen X., Cao F., Oleynikov P., Sun H. (2014). π–π interaction of aromatic groups in amphiphilic molecules directing for single-crystalline mesostructured zeolite nanosheets. Nat. Commun..

[144] Wu W., Tran D.T., Wu X., Oh S.C., Wang M., Chen H., Emdadi L., Zhang J., Schulman E., Liu D. (2019). Multilamellar and pillared titanium Silicalite-1 with long-range order of zeolite nanosheet layers: Synthesis and catalysis. Microporous Mesoporous Mater..

[145] Rubin M.K., Chu P. (1990). Composition of synthetic porous crystalline material, its synthesis and use. U.S. Patent.

[146] Roth W.J., Yorke T., Kerby M.C., Weston S.C. (2011). Molecular sieve composition (EMM-10-P), its method of making, and use for hydrocarbon conversions. U.S. Patent.

[147] Roth W.J., Dorset D.L., Kennedy G.J. (2011). Discovery of new MWW family zeolite EMM-10: Identification of EMM-10P as the missing MWW precursor with disordered layers. Microporous Mesoporous Mater..

[148] Millini R., Perego G., Parker W., Bellussi G., Carluccio L. (1995). Layered structure of ERB-1 microporous borosilicate precursor and its intercalation properties towards polar molecules. Microporous Mater..

[149] Wu P., Tatsumi T., Komatsu T., Yashima T. (2001). A novel titanosilicate with MWW structure. I. Hydrothermal synthesis, elimination of extraframework titanium, and characterizations. J. Phys. Chem. B.

[150] Fung A.S., Lawton S.L., Roth W.J. (1994). Synthetic layered MCM-56, its synthesis and use.

[151] Juttu G.G., Lobo R.F. (2000). Characterization and catalytic properties of MCM-56 and MCM-22 zeolites. Microporous Mesoporous Mater..

[152] Qian B., Gao H.X., Yang D.Q., Kong D.J. (2009). Preparation and structure characterization of UZM-8 zeolite templated by DEDMAOH. Acta Chim. Sin..

[153] Archer R.H., Carpenter J.R., Hwang S.-J., Burton A.W., Chen C.-Y., Zones S.I., Davis M.E. (2010). Physicochemical properties and catalytic behavior of the molecular sieve SSZ-70. Chem. Mater..

[154] Archer R.H., Zones S.I., Davis M.E. (2010). Imidazolium structure directing agents in zeolite synthesis: Exploring guest/host relationships in the synthesis of SSZ-70. Microporous Mesoporous Mater..

[155] Zones S.I., Burton A.W. (2006). Molecular sieve SSZ-70 composition of matter and synthesis thereof.

[156] Kubů M., Roth W.J., Greer H.F., Zhou W., Morris R.E., Přech J., Čejka J. (2013). A new family of two-dimensional zeolites prepared from the intermediate layered precursor IPC-3P obtained during the synthesis of TUN zeolite. Chem. Eur. J..

[157] Grzybek J., Roth W.J., Gil B., Korzeniowska A., Mazur M., Čejka J., Morris R.E. (2019). A new layered MWW zeolite synthesized with the bifunctional surfactant template and the updated classification of layered zeolite forms obtained by direct synthesis. J. Mater. Chem. A.

[158] Grünewald-Lüke A., Gies H., Müller U., Yilmaz B., Imai H., Tatsumi T., Xie B., Xiao F.-S., Bao X., Zhang W. (2012). Layered precursors for new zeolitic materials: Synthesis and characterization of B-RUB-39 and its condensation product B-RUB-41. Microporous Mesoporous Mater..

[159] Chen Y., Huang S., Wang X., Zhang L., Wu N., Liao F., Wang Y. (2017). Synthesis and characterization of a layered silicogermanate PKU-22 and its topotactic condensation to a three-dimensional STI-type zeolite. Cryst. Growth Des..

[160] Schreyeck L., Caullet P., Mougenel J.-C., Guth J.-L., Marler B. (1995). A layered microporous aluminosilicate precursor of FER-type zeolite. J. Am. Chem. Soc. Chem. Commun..

[161] Yang B., Jiang J.-G., Xu H., Liu Y., Peng H., Wu P. (2013). Selective skeletal isomerization of 1-butene over FER-type zeolites derived from PLS-3 lamellar precursors. Appl. Catal. A.

[162] Eilertsen E.A., Ogino I., Hwang S.-J., Rea T., Yeh S., Zones S.I., Katz A. (2011). Nonaqueous fluoride/chloride anion-promoted delamination of layered zeolite precursors: Synthesis and characterization of UCB-2. Chem. Mater..

[163] Yang B., Wu H., Wu P. (2014). Synthesis, characterization, and catalytic properties of interlayer expanded aluminosilicate IEZ-PLS-3. J. Phys. Chem. C.

[164] Yang B., Jiang J.-G., Xu H., Wu H., He M., Wu P. (2018). Synthesis of extra-large-pore zeolite ECNU-9 with intersecting 14*12-ring channels. Angew. Chem. Int. Ed..

[165] Yang B.T., Zou Q.Y. (2017). Post-synthesis and catalytic performance of novel interlayer expanded stanosilicate IEZ-Sn-PLS-3. Chem. Lett..

[166] Tsunoji N., Yuki S., Oumi Y., Sekikawa M., Sasaki Y., Sadakane M., Sano T. (2015). Design of microporous material HUS-10 with tunable hydrophilicity, molecular sieving, and CO_2_ adsorption ability derived from interlayer silylation of layered silicate HUS-2. ACS Appl. Mater. Interfaces.

[167] Tsunoji N., Ide Y., Yagenji Y., Sadakane M., Sano T. (2014). Design of layered silicate by grafting with metal acetylacetonate for high activity and chemoselectivity in photooxidation of cyclohexane. ACS Appl. Mater. Interfaces.

[168] Gies H., Müller U., Yilmaz B., Tatsumi T., Xie B., Xiao F.-S., Bao X., Zhang W., Vos D.D. (2011). Interlayer expansion of the layered zeolite precursor RUB-39: A universal method to synthesize functionalized microporous silicates. Chem. Mater..

[169] Tijsebaert B., Henry M., Gies H., Xiao F.-S., Zhang W., Bao X., Imai H., Tatsumi T., Müller U., Yilmaz B. (2011). Exploring the void structure and activity of RUB-39 based expanded materials using the hydroconversion of decane. J. Catal..

[170] Na K., Park W., Seo Y., Ryoo R. (2011). Disordered assembly of MFI zeolite nanosheets with a large volume of intersheet mesopores. Chem. Mater..

[171] Jeon M.Y., Kim D., Kumar P., Lee P.S., Rangnekar N., Bai P., Shete M., Elyassi B., Lee H.S., Narasimharao K. (2017). Ultra-selective high-flux membranes from directly synthesized zeolite nanosheets. Nature.

[172] Na K., Jo C., Kim J., Ahn W.-S., Ryoo R. (2011). MFI titanosilicate nanosheets with single-unit-cell thickness as an oxidation catalyst using peroxides. ACS Catal..

[173] Zhang X., Liu D., Xu D., Asahina S., Cychosz K.A., Agrawal K.V., Al Wahedi Y., Bhan A., Al Hashimi S., Terasaki O. (2012). Synthesis of self-pillared zeolite nanosheets by repetitive branching. Science.

[174] Singh B.K., Xu D., Han L., Ding J., Wang Y., Che S. (2014). Synthesis of single-crystalline mesoporous ZSM-5 with three-dimensional pores via the self-assembly of a designed triply branched cationic surfactant. Chem. Mater..

[175] Emdadi L., Wu Y., Zhu G., Chang C.-C., Fan W., Pham T., Lobo R.F., Liu D. (2014). Dual template synthesis of meso- and microporous MFI zeolite nanosheet assemblies with tailored activity in catalytic reactions. Chem. Mater..

[176] Přech J., Carretero Marta A., Čejka J. (2017). Baeyer–Villiger oxidation of cyclic ketones by using tin–silica pillared catalysts. ChemCatChem.

[177] Přech J., Eliášová P., Aldhayan D., Kubů M. (2015). Epoxidation of bulky organic molecules over pillared titanosilicates. Catal. Today.

[178] Galve A., Gorgojo P., Navascues N., Casado C., Tellez C., Coronas J. (2011). Structural study on the Al distribution in zeolites Nu-6(1) and Nu-6(2). Microporous Mesoporous Mater..

[179] Wei L., Song K., Wu W., Holdren S., Zhu G., Shulman E., Shang W., Chen H., Zachariah M.R., Liu D. (2019). Vapor-phase strategy to pillaring of two-dimensional zeolite. J. Am. Chem. Soc..

[180] Fermoso J., Hernando H., Jana P., Moreno I., Přech J., Ochoa-Hernández C., Pizarro P., Coronado J.M., Čejka J., Serrano D.P. (2016). Lamellar and pillared ZSM-5 zeolites modified with MgO and ZnO for catalytic fast-pyrolysis of eucalyptus woodchips. Catal. Today.

[181] Roth W.J. (2005). MCM-22 zeolite family and the delaminated zeolite MCM-56 obtained in one-step synthesis. Studies in Surface Science and Catalysis.

[182] Gil B., Makowski W., Marszalek B., Roth W.J., Kubů M., Čejka J., Olejniczak Z. (2014). High acidity unilamellar zeolite MCM-56 and its pillared and delaminated derivatives. Dalton Trans..

[183] Ogino I., Eilertsen E.A., Hwang S.-J., Rea T., Xie D., Ouyang X., Zones S.I., Katz A. (2013). Heteroatom-tolerant delamination of layered zeolite precursor materials. Chem. Mater..

[184] Roth W.J., Dorset D.L., Kennedy G.J., Yorke T., Helton T.E. (2010). A novel molecular sieve composition EMM-12, a method of making and a process of using the same.

[185] Runnebaum R.C., Ouyang X., Edsinga J.A., Rea T., Arslan I., Hwang S.-J., Zones S.I., Katz A. (2014). Role of delamination in zeolite-catalyzed aromatic alkylation: UCB-3 versus 3-D Al-SSZ-70. ACS Catal..

[186] Okrut A., Aigner M., Schöttle C., Grosso-Giordano N.A., Hwang S.-J., Ouyang X., Zones S., Katz A. (2018). SSZ-70 borosilicate delamination without sonication: Effect of framework topology on olefin epoxidation catalysis. Dalton Trans..

[187] Corma A., Fornés V., Guil J.M., Pergher S., Maesen T.L.M., Buglass J.G. (2000). Preparation, characterisation and catalytic activity of ITQ-2, a delaminated zeolite. Microporous Mesoporous Mater..

[188] Corma A., Diaz U., Fornes V.L., Jorda J., Domine M., Rey F. (1999). Ti/ITQ-2, a new material highly active and selective for the epoxidation of olefins with organic hydroperoxides. Chem. Commun..

[189] Chang C.-C., Lee J.-F., Cheng S. (2017). Highly catalytically active micro/meso-porous Ti-MCM-36 prepared by a grafting method. J. Mater. Chem. A.

[190] Jin F., Chang C.-C., Yang C.-W., Lee J.-F., Jang L.-Y., Cheng S. (2015). New mesoporous titanosilicate MCM-36 material synthesized by pillaring layered ERB-1 precursor. J. Mater. Chem. A.

[191] Jin F., Chen S.-Y., Jang L.-Y., Lee J.-F., Cheng S. (2014). New Ti-incorporated MCM-36 as an efficient epoxidation catalyst prepared by pillaring MCM-22 layers with titanosilicate. J. Catal..

[192] Margarit V.J., Martinez-Armero M.E., Navarro M.T., Martinez C., Corma A. (2015). Direct dual-template synthesis of MWW zeolite monolayers. Angew. Chem. Int. Ed..

[193] Roth W.J., Gil B., Makowski W., Sławek A., Korzeniowska A., Grzybek J., Siwek M., Michorczyk P. (2016). Framework-substituted cerium MCM-22 zeolite and its interlayer expanded derivative MWW-IEZ. Catal. Sci. Technol..

[194] Fan W., Wu P., Namba S., Tatsumi T. (2004). A titanosilicate that is structurally analogous to an MWW-type lamellar precursor. Angew. Chem. Int. Ed..

[195] Ruan J., Wu P., Slater B., Terasaki O. (2005). Structure elucidation of the highly active titanosilicate catalyst Ti-YNU-1. Angew. Chem. Int. Ed..

[196] Wang Y.X., Gies H., Marler B., Müller U. (2005). Synthesis and crystal structure of zeolite RUB-41 obtained as calcination product of a layered precursor: A systematic approach to a new synthesis route. Chem. Mater..

[197] Yilmaz B., Müller U., Tijsebaert B., De Vos D., Xie B., Xiao F.-S., Gies H., Zhang W., Bao X., Imai H. (2011). Al-RUB-41: A shape-selective zeolite catalyst from a layered silicate. Chem. Commun..

[198] Henkelis S.E., Mazur M., Rice C.M., Bignami G.P.M., Wheatley P.S., Ashbrook S.E., Čejka J., Morris R.E. (2019). A procedure for identifying possible products in the assembly–disassembly–organization–reassembly (ADOR) synthesis of zeolites. Nat. Protoc..

[199] Roth W.J., Shvets O.V., Shamzhy M., Chlubná P., Kubů M., Nachtigall P., Čejka J. (2011). Postsynthesis transformation of three-dimensional framework into a lamellar zeolite with modifiable architecture. J. Am. Chem. Soc..

[200] Bignami G.P.M., Dawson D.M., Seymour V.R., Wheatley P.S., Morris R.E., Ashbrook S.E. (2017). Synthesis, isotopic enrichment, and solid-state NMR characterization of zeolites derived from the assembly, disassembly, organization, reassembly process. J. Am. Chem. Soc..

[201] Wheatley P.S., Chlubná-Eliášová P., Greer H., Zhou W., Seymour V.R., Dawson D.M., Ashbrook S.E., Pinar A.B., McCusker L.B., Opanasenko M. (2014). Zeolites with continuously tuneable porosity. Angew. Chem. Int. Ed..

[202] Mazur M., Wheatley P., Navarro M., Roth W., Položij M., Mayoral A., Eliášová P., Nachtigall P., Čejka J., Morris R. (2015). Synthesis of ‘unfeasible’ zeolites. Nat. Chem..

[203] Shamzhy M., Mazur M., Opanasenko M., Roth W.J., Čejka J. (2014). Swelling and pillaring of the layered precursor IPC-1P: Tiny details determine everything. Dalton Trans..

[204] Mazur M., Chlubná-Eliášová P., Roth W.J., Čejka J. (2014). Intercalation chemistry of layered zeolite precursor IPC-1P. Catal. Today.

[205] Přech J., Čejka J. (2016). UTL titanosilicate: An extra-large pore epoxidation catalyst with tunable textural properties. Catal. Today.

[206] Navarro M., Morris S.A., Mayoral A., Čejka J., Morris R.E. (2017). Microwave heating and the fast ADOR process for preparing zeolites. J. Mater. Chem. A.

[207] Kasneryk V., Shamzhy M., Opanasenko M., Wheatley P.S., Morris S.A., Russell S.E., Mayoral A., Trachta M., Čejka J., Morris R.E. (2017). Expansion of the ADOR strategy for the synthesis of zeolites: The synthesis of IPC-12 from zeolite UOV. Angew. Chem. Int. Ed..

[208] Kasneryk V., Shamzhy M., Opanasenko M., Wheatley P.S., Morris R.E., Čejka J. (2018). Insight into the ADOR zeolite-to-zeolite transformation: The UOV case. Dalton Trans..

[209] Firth D.S., Morris S.A., Wheatley P.S., Russell S.E., Slawin A.M.Z., Dawson D.M., Mayoral A., Opanasenko M., Položij M., Čejka J. (2017). Assembly–disassembly–organization–reassembly synthesis of zeolites based on *cfi*-type layers. Chem. Mater..

[210] Shamzhy M., Opanasenko M., Tian Y., Konysheva K., Shvets O., Morris R.E., Čejka J. (2014). Germanosilicate precursors of ADORable zeolites obtained by disassembly of ITH, ITR, and IWR zeolites. Chem. Mater..

[211] Shvets O.V., Konysheva K.M., Shamzhy M.V., Opanasenko M.V., Yaremov P.S., Xiao C., Zou X., Čejka J. (2019). Mordenite nanorods and nanosheets prepared in presence of gemini type surfactants. Catal. Today.

[212] Derouane E.G., Védrine J.C., Pinto R.R., Borges P.M., Costa L., Lemos M.A.N.D.A., Lemos F., Ribeiro F.R. (2013). The acidity of zeolites: Concepts, measurements and relation to catalysis: A review on experimental and theoretical methods for the study of zeolite acidity. Catal. Rev..

[213] Sandoval-Díaz L.-E., González-Amaya J.-A., Trujillo C.-A. (2015). General aspects of zeolite acidity characterization. Microporous Mesoporous Mater..

[214] Freitas C., Barrow N.S., Zholobenko V. (2018). Accessibility and location of acid sites in zeolites as probed by fourier transform infrared spectroscopy and magic angle spinning nuclear magnetic resonance. Johnson Matthey Technol. Rev..

[215] Fyfe C.A., Gobbi G.C., Hartman J.S., Klinowski J., Thomas J.M. (1982). Solid-state magic-angle spinning. Aluminum-27 nuclear magnetic resonance studies of zeolites using a 400-MHz high-resolution spectrometer. J. Phys. Chem..

[216] Majano G., Delmotte L., Valtchev V., Mintova S. (2009). Al-Rich zeolite beta by seeding in the absence of organic template. Chem. Mater..

[217] Gilson J.-P., Edwards G.C., Peters A.W., Rajagopalan K., Wormsbecher R.F., Roberie T.G., Shatlock M.P. (1987). Penta-co-ordinated aluminium in zeolites and aluminosilicates. J. Am. Chem. Soc. Chem. Commun..

[218] Jo C., Cho K., Kim J., Ryoo R. (2014). MFI zeolite nanosponges possessing uniform mesopores generated by bulk crystal seeding in the hierarchical surfactant-directed synthesis. Chem. Commun..

[219] Lacarriere A., Luck F., Świerczyński D., Fajula F., Hulea V. (2011). Methanol to hydrocarbons over zeolites with MWW topology: Effect of zeolite texture and acidity. Appl. Catal. A.

[220] Jiang M., Karge H.G. (1996). Investigation of acid properties of dealuminated H-mordenite zeolites by low-temperature diffuse reflectance FTIR. J. Am. Chem. Soc. Faraday Trans..

[221] Busca G. (1998). Spectroscopic characterization of the acid properties of metal oxide catalysts. Catal. Today.

[222] Trombetta M., Busca G. (1999). On the characterization of the external acid sites of ferrierite and other zeolites: A reply to Pieterse et al. J. Catal..

[223] Dapsens P.Y., Mondelli C., Jagielski J., Hauert R., Pérez-Ramírez J. (2014). Hierarchical Sn-MFI zeolites prepared by facile top-down methods for sugar isomerisation. Catal. Sci. Technol..

[224] Schallmoser S., Ikuno T., Wagenhofer M.F., Kolvenbach R., Haller G.L., Sanchez-Sanchez M., Lercher J.A. (2014). Impact of the local environment of Brønsted acid sites in ZSM-5 on the catalytic activity in n-pentane cracking. J. Catal..

[225] Bai Y., Wei L., Yang M., Chen H., Holdren S., Zhu G., Tran D.T., Yao C., Sun R., Pan Y. (2018). Three-step cascade over a single catalyst: Synthesis of 5-(ethoxymethyl)furfural from glucose over a hierarchical lamellar multi-functional zeolite catalyst. J. Mater. Chem. A.

[226] Laforge S., Ayrault P., Martin D., Guisnet M. (2005). Acidic and catalytic properties of MCM-22 and MCM-36 zeolites synthesized from the same precursors. Appl. Catal. A.

[227] Arean C.O., Delgado M.R., Nachtigall P., Thang H.V., Rubeš M., Bulánek R., Chlubná-Eliášová P. (2014). Measuring the brønsted acid strength of zeolites—Does it correlate with the O–H frequency shift probed by a weak base?. Phys. Chem. Chem. Phys..

[228] Nesterenko N.S., Thibault-Starzyk F., Montouillout V., Yuschenko V.V., Fernandez C., Gilson J.P., Fajula F., Ivanova I.I. (2004). Accessibility of the acid sites in dealuminated small-port mordenites studied by FTIR of co-adsorbed alkylpyridines and CO. Microporous Mesoporous Mater..

[229] Niwa M., Katada N. (2013). New method for the temperature- programmed desorption (TPD) of ammonia experiment for characterization of zeolite acidity: A review. Chem. Rec..

[230] Rakiewicz E.F., Peters A.W., Wormsbecher R.F., Sutovich K.J., Mueller K.T. (1998). Characterization of acid sites in zeolitic and other inorganic systems using solid-state ^31^P NMR of the probe molecule trimethylphosphine oxide. J. Phys. Chem. B.

[231] Duan J., Higuchi M., Krishna R., Kiyonaga T., Tsutsumi Y., Sato Y., Kubota Y., Takata M., Kitagawa S. (2014). High CO_2_/N_2_/O_2_/CO separation in a chemically robust porous coordination polymer with low binding energy. Chem. Sci..

[232] Wu Y., Emdadi L., Qin D., Zhang J., Liu D. (2017). Quantification of external surface and pore mouth acid sites in unit-cell thick pillared MFI and pillared MWW zeolites. Microporous Mesoporous Mater..

[233] Pelmenschikov A.G., van Santen R.A., Janchen J., Meijer E. (1993). Acetonitrile-d3 as a probe of Lewis and Broensted acidity of zeolites. J. Phys. Chem..

[234] Pereira C., Gorte R.J. (1992). Method for distinguishing Brønsted-acid sites in mixtures of H-ZSM-5, H-Y and silica-alumina. Appl. Catal. A.

[235] Góra-Marek K., Tarach K., Choi M. (2014). 2,6-Di-tert-butylpyridine sorption approach to quantify the external acidity in hierarchical zeolites. J. Phys. Chem. C.

[236] Bevilacqua M., Busca G. (2002). A study of the localization and accessibility of Brønsted and Lewis acid sites of H-mordenite through the FT-IR spectroscopy of adsorbed branched nitriles. Catal. Commun..

[237] Ordomsky V.V., Murzin V.Y., Monakhova Y.V., Zubavichus Y.V., Knyazeva E.E., Nesterenko N.S., Ivanova I.I. (2007). Nature, strength and accessibility of acid sites in micro/mesoporous catalysts obtained by recrystallization of zeolite BEA. Microporous Mesoporous Mater..

[238] Ayrault P., Datka J., Laforge S., Martin D., Guisnet M. (2004). Characterization of the Internal and External Acidity of H-MCM-22 Zeolites. J. Phys. Chem. B.

[239] Hsia Chen C.S., Schramm S.E. (1996). Type and catalytic activity of surface acid sites of medium and large pore zeolites Their deactivation with bulky organophosphorus compounds. Microporous Mater..

[240] Thang H.V., Rubeš M., Bludský O., Nachtigall P. (2014). Computational investigation of the Lewis acidity in three-dimensional and corresponding two-dimensional zeolites: UTL vs. IPC-1P. J. Phys. Chem. A.

[241] Ramos F.S.O., Pastore H.O. (2017). 2D-to-disguised 3D materials with built-in acid sites: H^+^-[Al]-RUB-18. Dalton Trans..

[242] Ramli Z., Yusoff N., Hamdan H. (2007). Delaminated zeolite, ITQ-6 as heterogeneous catalyst for Friedel Crafts alkylation. Mala. J. Anal. Sci..

[243] De Pietre M.K., Bonk F.A., Rettori C., Garcia F.A., Pastore H.O. (2011). [V,Al]-ITQ-6: Novel porous material and the effect of delamination conditions on V sites and their distribution. Microporous Mesoporous Mater..

[244] Yang B., Jiang J.-G., Xu H., Ji P., Wu P. (2015). Sub-zeolite of FER topology derived from an interlayer modification of PLS-3 lamellar precursor. Microporous Mesoporous Mater..

[245] Liu D., Bhan A., Tsapatsis M., Al Hashimi S. (2011). Catalytic behavior of brønsted acid sites in MWW and MFI zeolites with dual meso- and microporosity. ACS Catal..

[246] Bleken B.-T.L., Wragg D.S., Arstad B., Gunnæs A.E., Mouzon J., Helveg S., Lundegaard L.F., Beato P., Bordiga S., Olsbye U. (2013). Unit cell thick nanosheets of zeolite H-ZSM-5: Structure and activity. Top. Catal..

[247] Jo C., Ryoo R., Žilková N., Vitvarová D., Čejka J. (2013). The effect of MFI zeolite lamellar and related mesostructures on toluene disproportionation and alkylation. Catal. Sci. Technol..

[248] Wu L.L., Magusin P.C.M.M., Degirmenci V., Li M.Q., Almutairi S.M.T., Zhu X.C., Mezari B., Hensen E.J.M. (2014). Acidic properties of nanolayered ZSM-5 zeolites. Microporous Mesoporous Mater..

[249] Meng L., Zhu X., Mezari B., Pestman R., Wannapakdee W., Hensen E.J.M. (2017). On the role of acidity in bulk and nanosheet [T]MFI (T = Al^3+^, Ga^3+^, Fe^3+^, B^3+^) zeolites in the methanol-to-hydrocarbons reaction. ChemCatChem.

[250] Bleken B.-T.L., Mino L., Giordanino F., Beato P., Svelle S., Lillerud K.P., Bordiga S. (2013). Probing the surface of nanosheet H-ZSM-5 with FTIR spectroscopy. Phys. Chem. Chem. Phys..

[251] Liu D., Zhang X., Bhan A., Tsapatsis M. (2014). Activity and selectivity differences of external Brønsted acid sites of single-unit-cell thick and conventional MFI and MWW zeolites. Microporous Mesoporous Mater..

[252] Wu Y., Lu Z., Emdadi L., Oh S.C., Wang J., Lei Y., Chen H., Tran D.T., Lee I.C., Liu D. (2016). Tuning external surface of unit-cell thick pillared MFI and MWW zeolites by atomic layer deposition and its consequences on acid-catalyzed reactions. J. Catal..

[253] Kim K., Ryoo R., Jang H.-D., Choi M. (2012). Spatial distribution, strength, and dealumination behavior of acid sites in nanocrystalline MFI zeolites and their catalytic consequences. J. Catal..

[254] Ji Y., Shi B., Yang H., Yan W. (2017). Synthesis of isomorphous MFI nanosheet zeolites for supercritical catalytic cracking of n-dodecane. Appl. Catal. A.

[255] Xu M., Mukarakate C., Iisa K., Budhi S., Menart M., Davidson M., Robichaud D.J., Nimlos M.R., Trewyn B.G., Richards R.M. (2017). Deactivation of multilayered mfi nanosheet zeolite during upgrading of biomass pyrolysis vapors. ACS Sustain. Chem. Eng..

[256] Kresge C.T., Roth W.J., Simmons K.G., Vartuli J.C. (1992). A method of preparing a pillared layered oxide material.

[257] Kresge C.T., Roth W.J. (1994). Method for preparing a pillared layered oxide material. U.S. Patent.

[258] Corma A., Fornés V., Martínez-Triguero J., Pergher S.B. (1999). Delaminated zeolites: Combining the benefits of zeolites and mesoporous materials for catalytic uses. J. Catal..

[259] Min H.-K., Park M.B., Hong S.B. (2010). Methanol-to-olefin conversion over H-MCM-22 and H-ITQ-2 zeolites. J. Catal..

[260] Maheshwari S., Martínez C., Teresa Portilla M., Llopis F.J., Corma A., Tsapatsis M. (2010). Influence of layer structure preservation on the catalytic properties of the pillared zeolite MCM-36. J. Catal..

[261] Zhang Z., Zhu W., Zai S., Jia M., Zhang W., Wang Z. (2013). Synthesis, characterization and catalytic properties of MCM-36 pillared via the MCM-56 precursor. J. Porous Mater..

[262] Schenkel R., Barth J.O., Kornatowski J., Lercher J.A., Aiello R., Giordano G., Testa F. (2002). Chemical and structural aspects of the transformation of the MCM-22 precursor into ITQ-2. Studies in Surface Science and Catalysis.

[263] Onida B., Borello L., Bonelli B., Geobaldo F., Garrone E. (2003). IR study of the acidity of ITQ-2, an “all-surface” zeolitic system. J. Catal..

[264] Chlubná P., Roth W.J., Zukal A., Kubů M., Pavlatová J. (2012). Pillared MWW zeolites MCM-36 prepared by swelling MCM-22P in concentrated surfactant solutions. Catal. Today.

[265] Purova R., Narasimharao K., Ahmed N.S.I., Al-Thabaiti S., Al-Shehri A., Mokhtar M., Schwieger W. (2015). Pillared HMCM-36 zeolite catalyst for biodiesel production by esterification of palmitic acid. J. Mol. Catal. A.

[266] Carriço C.S., Cruz F.T., dos Santos M.B., Oliveira D.S., Pastore H.O., Andrade H.M.C., Mascarenhas A.J.S. (2016). MWW-type catalysts for gas phase glycerol dehydration to acrolein. J. Catal..

[267] Käldström M., Kumar N., Heikkilä T., Murzin D.Y. (2011). Pillared H-MCM-36 mesoporous and H-MCM-22 microporous materials for conversion of levoglucosan: Influence of varying acidity. Appl. Catal. A.

[268] Rutkowska M., Díaz U., Palomares A.E., Chmielarz L. (2015). Cu and Fe modified derivatives of 2D MWW-type zeolites (MCM-22, ITQ-2 and MCM-36) as new catalysts for DeNO_x_ process. Appl. Catal. B.

[269] Roth W.J., Chlubná P., Kubů M., Vitvarová D. (2013). Swelling of MCM-56 and MCM-22P with a new medium—Surfactant–tetramethylammonium hydroxide mixtures. Catal. Today.

[270] Hu B., Gay I.D. (1999). Probing surface acidity by ^31^P nuclear magnetic resonance spectroscopy of arylphosphines. Langmuir.

[271] Wang Y., Zhuang J., Yang G., Zhou D., Ma D., Han X., Bao X. (2004). Study on the external surface acidity of MCM-22 zeolite: theoretical calculation and ^31^P MAS NMR. J. Phys. Chem. B.

[272] Žilková N., Eliášová P., Al-Khattaf S., Morris R.E., Mazur M., Čejka J. (2016). The effect of UTL layer connectivity in isoreticular zeolites on the catalytic performance in toluene alkylation. Catal. Today.

[273] Wan Y., Zhao D. (2007). On the controllable soft-templating approach to mesoporous silicates. Chem. Rev..

[274] Zhang K., Ostraat M.L. (2016). Innovations in hierarchical zSeolite synthesis. Catal. Today.

